# GIGANTEA is a co-chaperone which facilitates maturation of ZEITLUPE in the *Arabidopsis* circadian clock

**DOI:** 10.1038/s41467-016-0014-9

**Published:** 2017-02-23

**Authors:** Joon-Yung Cha, Jeongsik Kim, Tae-Sung Kim, Qingning Zeng, Lei Wang, Sang Yeol Lee, Woe-Yeon Kim, David E. Somers

**Affiliations:** 1 Division of Applied Life Science (BK21Plus), PMBBRC &IALS, Gyeongsang National University, Jinju, 52828 Republic of Korea; 2 Department of Molecular Genetics, The Ohio State University, Columbus, 43210 USA; 3 Center for Plant Aging Research, Institute for Basic Science (IBS), Daegu, 711-873 Republic of Korea; 4 Department of Agricultural Sciences, Korea National Open University, Seoul, 03087 Republic of Korea; 5 Key Laboratory of Plant Molecular Physiology, Institute of Botany, Chinese Academy of Sciences, Beijing, 100093 China

## Abstract

Circadian clock systems help establish the correct daily phasing of the behavioral, developmental, and molecular events needed for the proper coordination of physiology and metabolism. The circadian oscillator comprises transcription–translation feedback loops but also requires post-translational processes that regulate clock protein homeostasis. GIGANTEA is a unique plant protein involved in the maintenance and control of numerous facets of plant physiology and development. Through an unknown mechanism GIGANTEA stabilizes the F-box protein ZEITLUPE, a key regulator of the circadian clock. Here, we show that GIGANTEA has general protein chaperone activity and can act to specifically facilitate ZEITLUPE maturation into an active form in vitro and in planta. GIGANTEA forms a ternary complex with HSP90 and ZEITLUPE and its co-chaperone action synergistically enhances HSP90/HSP70 maturation of ZEITLUPE in vitro. These results identify a molecular mechanism for GIGANTEA activity that can explain its wide-ranging role in plant biology.

## Abstract (web summary)

The plant-specific GIGANTEA protein regulates the circadian clock by stabilizing the F-box protein ZEITLUPE via an unknown mechanism. Here Cha *et al*. show that GIGANTEA has intrinsic chaperone activity and can facilitate ZEITLUPE maturation by acting synergistically with HSP90.

## Introduction

The circadian system is a 24 h timing mechanism common to most organisms on earth. Through a combination of transcriptional, translational, and post-translational processes, the circadian clock controls the phasing of gene expression, metabolism, and physiology to help optimize an organism’s fit to its environment. In *Arabidopsis*, a mutually repressive negative feedback loop comprising the evening-expressed gene of TIMING OF CAB EXPRESION 1 (TOC1) and morning-expressed genes CIRCADIAN CLOCK ASSOCIATED 1/LATE ELONGATED HYPOCOTYL (CCA1/LHY) is one of the core components of the oscillator. Additional transcriptional repressors/co-repressors (PSEUDO-RESPONSE REGULATOR (PRR) 5, 7 and 9, TOPLESS (TPL), LUX ARRHYTHMO (LUX)) and activators/co-activators (REVEILLE (RVE) 4, RVE6, NIGHT LIGHT–INDUCIBLE AND CLOCK-REGULATED 1(LNK1), LNK2) are further necessary in establishing both proper period and robustness of the circadian clock^1^.

In all known circadian systems both post-transcriptional and post-translational processes are essential to clock function^2–4^. In *Arabidopsis* the F-box protein ZEITLUPE (ZTL) specifies an evening-phased E3 ubiquitin ligase (SCF^ZTL^) that targets TOC1 and PRR5 for proteasomal degradation^5–7^. ZTL and related family members (FKF1 and LKP2) are unique among known F-box proteins in possessing a blue-light sensing domain [LIGHT, OXYGEN, VOLTAGE (LOV)] at the N-terminus, which facilitates their stability^8^. The large (1173 aa) single-gene encoded protein, GIGANTEA (GI) interacts with the ZTL LOV domain to post-translationally stabilize ZTL in blue light^9^. Circadian oscillations of *GI* mRNA^10,11^ result in an evening-phased peak in GI protein abundance, which establishes and sustains a rhythm of ZTL abundance that is in phase with GI cycling^9,12^. These oscillations in ZTL help maintain high-amplitude oscillations of TOC1 and PRR5^6,9^.

Among the components required to sustain the plant circadian oscillator, GI is one of the few for which no molecular or biochemical function has been determined. It is highly conserved among vascular plants (Supplementary Fig. 1)^13^ and plays numerous roles in plant physiology and development, including the control of flowering time, hypocotyl elongation, circadian period, carbohydrate metabolism, salt tolerance, and other physiological processes^14–19^. Certain *gi* mutant alleles exhibit diametrically opposite phenotypes, indicating clearly separable roles for GI, and complexity and nuance in its many functions in the plant^11,19–21^. *GIGANTEA* mRNA and protein are clock-controlled and GI is found both in the cytosol and nucleus^9–11,14,20,22,23^. Under diurnal conditions GI forms unique nuclear bodies with EARLY FLOWERING 4 (ELF4) that dynamically oscillate in abundance^24^. GI protein regulation is poorly understood, but interaction with EARLY FLOWERING 3 (ELF3) and CONSTITUTIVE PHOTOMORPHOGENIC 1 (COP1) affects GI stability, and GI levels drop in the absence of ZTL^22,25^.

The fully functional state of all proteins requires correct three-dimensional folding either during or shortly after synthesis. In the highly protein-dense cellular environment misfolded proteins may form into unproductive aggregates. To prevent such non-native associations and to facilitate protein folding, cells possess a wide range of molecular chaperones that are essential to the proper maturation of a substantial number of proteins. Molecular chaperones can be defined as any protein that interacts, stabilizes, or helps a non-native protein to acquire its native conformation but is not present in the final functional structure^26,27^. These include the well-known and ubiquitous HSP70 and HSP90 chaperone systems, and the extensive chaperonin family (e.g., GroEL in bacteria; Cpn60 in chloroplasts)^28,29^. Co-chaperones are additional protein factors that pair with specific chaperones to confer specificity to the individual protein targets (clients)^30,31^.

Here we establish the molecular function of GI as a chaperone/co-chaperone that associates with HSP90 to facilitate ZTL maturation into a fully functional protein in vitro and in vivo. GI forms a ternary complex with HSP90 and ZTL and synergistically enhances the effectiveness of the HSP70/HSP90-mediated maturation of ZTL. These findings suggest that the wide-ranging role of GI in plant biology may arise from its function as a co-chaperone that helps specify particular HSP90 clients from the extremely broad spectrum of proteins subject to the HSP90 chaperone cycle.

## Results

### GI exhibits general chaperone activity in vitro

Previous work demonstrated that both HSP90 and GI are required for ZTL protein accumulation and that ZTL is a client of the chaperone HSP90^9,32^. These findings suggested that GI and HSP90 might co-regulate ZTL in the same pathway and act similarly and together. Whereas HSP90 is constitutively expressed, the circadian and diel oscillations in GI expression could consequently confer post-translational oscillations in ZTL levels by contributing to the maturation and stabilization of ZTL polypeptide.

To investigate a potential role for GI as a molecular chaperone, we first tested GI for general chaperone ability. Molecular chaperones possess a certain set of properties, some of which can be tested in vitro using generic substrates. These include the ability to recognize and bind unfolded proteins, to suppress aggregation during protein unfolding and folding, to influence the yield of folding, and to perform the second and third functions at stoichiometric levels^26^. The holdase concept tests whether the second property of a candidate chaperone—the ability to bind the substrate and inhibit aggregation—is satisfied. Generic model substrates such as malate dehydrogenase (MDH)^33–35^, citrate synthase, and others are used to routinely to test this in vitro, as peptide binding studies with different chaperones show that most exhibit a greater preference for hydrophobic peptides than for charged, hydrophilic peptides^26,35^.

Adequate amounts of full-length, soluble GI could not be purified from *Escherichia coli* (*E. coli*), but an extensive GI N-terminal polypeptide (aa 1–858, 73% of the protein; GI^N^) that includes a functional ZTL–interaction domain^18^ was successfully obtained. We first tested GI^N^ by transient expression in planta for two key aspects of GI effects on ZTL: interaction specificity and stabilization. First, HA-GI^N^ was able to recapitulate the specificity of full-length GI–ZTL protein interactions (co-immunoprecipitation) in *Nicotiana benthamiana* transient expression assays (Supplementary Fig. 2). The photochemically dead ZTL^C82A^ allele interacts poorly with full-length GI and the same was found for GI^N^ (Supplementary Fig. 2). Second, ZTL protein abundance was enhanced when GI^N^ was transiently co-expressed with ZTL in *Arabidopsis* protoplasts. This was to the same extent as obtained for full-length GI and both polypeptides more poorly stabilized ZTL^C82A^ (Supplementary Fig. 3). The non-interacting C-terminal region of GI (aa 920–1173; GI^C^) failed in both assays (Supplementary Figs. 2, 3).

We next tested soluble in vitro purified GI^N^ and GI^C^ containing an N-terminal maltose-binding protein (MBP) tag to facilitate solubility and purification (Supplementary Fig. 4). To guard against the effects of co-purifying *E. coli* proteins, we determined the composition of each of the three bands by MALDI-TOF/TOF-MS. Only the appropriate regions of GI and MBP were identified (Supplementary Table 1).

**Fig. 1:**
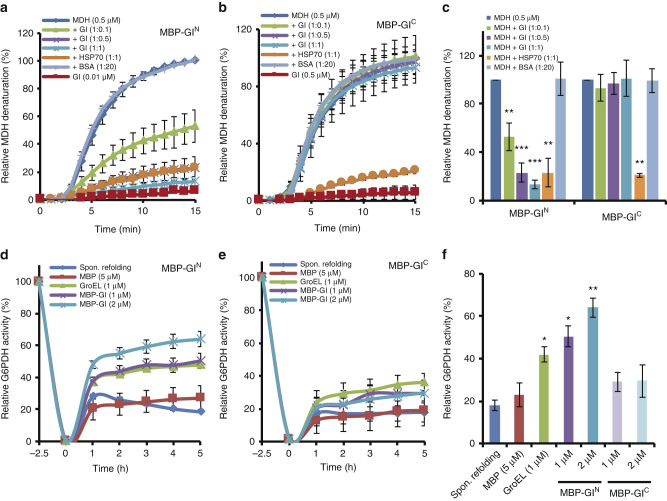
**GI** ^**N**^ **exhibits general molecular chaperone activity in vitro. a** MBP-GI^N^ decreases heat-mediated MDH aggregation with increasing stoichiometric parity. **b** MBP-GI^C^ has no effect on heat-mediated MDH aggregation. Both GI polypeptides were tested using MDH (0.5 μM) as a model substrate under thermal denaturing conditions (45 °C) in various molar ratios. HSP70 and BSA used as positive and negative controls, respectively. **c** The mean MDH denaturation state at the treatment endpoint of **a** and **b** relative to thermal-denaturation of MDH alone. The holdase assay (**a**–**c**) measures the aggregation of the model substrate MDH (0.5 μM), by measuring the turbidity (light scattering) at 340 nm under thermal denaturing conditions for 15 min at 45 °C. The turbidity of MDH alone at 15 min was set to 100%, and that from each treatment expressed relative to it. **d** MBP-GI^N^ refolds chemically denatured G6PDH. **e** MBP-GI^C^ cannot refold chemically denatured G6PDH. **f** The mean G6PDH activity at the treatment endpoint of **d** and **e** relative to the activity of undenatured G6PDH. The foldase assay determines G6PDH activity by measuring absorbance at 340 nm (Abs_340_) from NADPH formation. G6PDH was denatured in 4 M guanidine-HCl for 2.5 h (−2.5 h), and the relative G6PDH activity (compared to native G6PDH activity, set to 100%) was monitored in the absence (Spon. Refolding, spontaneous refolding) or presence of MBP, GroEL, and MBP-GI^N^ or MBP-GI^C^ for 5 h in renaturation buffer. GroEL and MBP were used as a positive and negative control, respectively. *P < 0.05; **P < 0.01; ***P < 0.001; two-tailed Student’s *t*-test. Data are means ± s.e. (*n* = 3)

GI^N^ effectively prevents heat-denatured aggregation of MDH in vitro (Fig. 1). As the ratio of GI^N^ to MDH was increased more MDH remained soluble and the proportion of heat-denatured MDH aggregates decreased, with GI^N^ similarly effective as HSP70 at the same 1:1 MDH:chaperone ratio (Fig. 1a, c). The non-interacting C-terminal region of GI (GI^C^) (Supplementary Figs. 2, 3) was ineffective at all stoichiometries (Fig. 1b, c). The MBP tag alone was not able to enhance the solubility of MDH (Supplementary Fig. 5) and BSA alone did not enhance aggregation (Supplementary Fig. 6). Similar results were obtained using His-GI^N^ and His-GI^C^ (Supplementary Fig. 7). These findings show GI^N^ possesses general holdase activity.

We next asked whether GI can effectively aid in the refolding of a denatured substrate back into an enzymatically active state. Polypeptides with such abilities are referred to as possessing foldase activity^35^. Glucose 6-phosphate dehydrogenase (G6PDH) is often used as a model substrate for this test and G6PDH in vitro refolding can be facilitated by several chaperones^36–38^. Chemically denatured G6PDH was tested for the return of enzyme activity in the presence of GI^N^, the MBP tag alone, GroEL (positive control) or buffer alone. The bacterial protein GroEL is a well-studied paradigm of the chaperonin class of molecular chaperones and has been used previously as a positive control for foldase activity^39,40^. GI^N^ equaled or exceeded the ability of GroEL to renature G6PDH to a level three times higher than that achieved by spontaneous renaturation, while GI^C^ was ineffective (Fig. 1d–f). Taken together, these results demonstrate an inherent chaperone activity for GI^N^.

### ZTL is a client of a GI/HSP90 chaperone complex in vitro

**Fig. 2:**
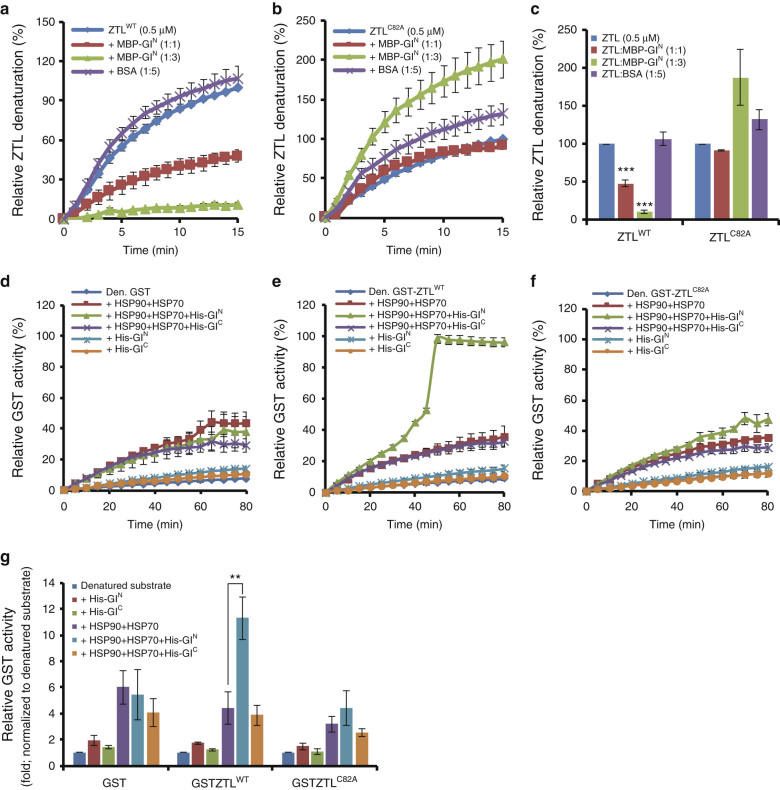
**ZTL is a specific client of GI** ^**N**^ **chaperone activity in vitro. a** MBP-GI^N^ decreases heat-mediated ZTL^WT^ aggregation with increasing levels of MBP-GI^N^. **b** MBP-GI^N^ has no effect or increases heat-mediated aggregation of the ZTL^C82A^ allele. **c** The mean denaturation state at the treatment endpoint of **a** and **b** relative to thermal-denaturation of ZTL^WT^ or ZTL^C82A^ alone. BSA used as a non-specific protein control. Holdase activity of MBP-GI^N^ was measured as the change in turbidity at 340 nm (aggregation of ZTL^WT^ or ZTL^C82A^ (0.5 μM)) under thermal denaturing conditions (45 °C) for 15 min. The value of ZTL^WT^ or ZTL^C82A^ alone at 15 min was set to 100%, and turbidity at 340 nm from each treatment expressed relative to it. BSA used as a non-specific protein control. **d**–**f** GI acts synergistically with HSP90 and HSP70 to reactivate denatured GST-ZTL. **d** GST, **e** GST-ZTL^WT^, and **f** GST-ZTL^C82A^ were heat-denatured at 45 °C and immediately mixed with His-GI^N^ (0.05 μM) or His-GI^C^ (0.05 μM) in the absence or presence of HSP90 (0.1 μM) and HSP70 (0.5 μM). Enzyme activity of undenatured GST, GST-ZTL^WT^, and GST-ZTL^C82A^ was set to 100% for **d**, **e** and **f**, respectively. **g** Mean GST activity at the treatment endpoint of **d**–**f** was normalized to the spontaneously refolding value of denatured GST or GST-fusions set to 1. The foldase assay determined GST activity by measuring the formation of a GS-DNB conjugate (GST reaction product) as determined by absorbance at 340 nm (Abs_340_). **P < 0.01; ***P < 0.001; two-tailed Student’s *t*-test. Data are means ± s.e. (*n* = 3)

We next determined whether ZTL is a specific client of GI^N^ in vitro. We performed holdase experiments using wild-type ZTL (ZTL^WT^) and the C82A variant (ZTL^C82A^) that eliminates photochemical activity and significantly reduces GI/ZTL interactability in planta^9^. At a 1:1 stoichiometry GI^N^ reduces ZTL^WT^ aggregation by 50% and at a 1:3 ZTL: GI^N^ ratio ZTL^WT^ remains fully soluble (Fig. 2a, c). In contrast, GI^N^ is unable to prevent ZTL^C82A^ denaturation even at higher GI^N^ concentrations (Fig. 2b, c). These results show that a specific interaction with ZTL is required for GI^N^ holdase function.

To determine if GI^N^ can specifically refold ZTL in vitro we used a glutathione S-transferase (GST)-ZTL fusion protein and observed the ability of GI^N^ to restore GST activity after heat denaturation. As an F-box protein that is part of a much larger SCF (Skip1/Cullin/F-box) complex, ZTL does not have an inherent enzyme activity that can be assayed to assess proper refolding. Given that a specific GI–ZTL interaction is needed for holdase activity (Fig. 2a–c), we reasoned that GI^N^-dependent restoration of GST enzyme activity to denatured GST-ZTL would reflect the restoration of ZTL to its native configuration. We used the artificial substrate 1-chloro-2,4-dinitrobenzene (CDNB) to fluorometrically assess GST activity^41,42^. In these experiments, we also included HSP90 and HSP70, based on our earlier findings that implicate HSP90 in ZTL maturation^32^ and the co-elution of HSP90, ZTL, and GI as large protein complexes in planta (Supplementary Fig. 8c, d). Additionally, HSP70 often acts together with HSP90 as an early step in an HSP70/HSP90 chaperoning cascade^43^. Using denatured GST alone as a negative control, we found that a mixture of HSP90 + HSP70 can restore ca. 40% of GST activity, while GI^N^ and GI^C^ alone are ineffective. Inclusion of GI^N^ or GI^C^ to the HSP70/HSP90 mixture does not enhance this effect (Fig. 2d, g). In contrast, the enzyme activity of denatured GST-ZTL is completely restored when GI^N^ is included in the assay mixture with HSP90 + HSP70, while GI^C^ has no such synergistic effect (Fig. 2e, g). Importantly, when GST-ZTL^C82A^ is used under the same conditions no significant increase in GST activity is observed (Fig. 2f). These results indicate that a specific interaction between ZTL^WT^ and GI^N^ is necessary for the full restoration of GST activity in GST-ZTL and that GI^N^ acts synergistically with HSP90 and HSP70. This indicates that in vitro GI can act as a co-chaperone with HSP90/HSP70 in the maturation of fully functional ZTL.

### GI is necessary for full maturation of ZTL activity in vivo

We further extended these studies in transgenic *Arabidopsis* by assessing the effectiveness of ZTL-luciferase (*35S:ZTL-LUC*) translational fusion enzyme activity in WT and *gi* mutant backgrounds. Luciferase enzyme activity has been used widely for measuring the status of proper protein folding both in vitro and in vivo^44–46^. We reasoned that a measure of properly folded/matured ZTL protein could come from observing the ratio of ZTL-LUC luciferase activity (by luminometry) to the level of ZTL-LUC protein levels (by immunoblotting), which we term ZTL-LUC-specific activity.

We first validated that the ZTL-LUC fusion protein recapitulates features of endogenous ZTL in planta in three ways. Endogenous ZTL oscillates with peak expression at ZT13 and lowest levels at ZT1 in light/dark cycles^9^. We determined that ZTL-LUC protein similarly oscillates by measuring protein levels at both time points, validating that this fusion protein is post-transcriptionally regulated as endogenous ZTL (Supplementary Fig. 9a, lanes 1 and 2). Importantly, ZTL-LUC level is constitutively diminished in *gi-201* relative to WT, consistent with a low level of endogenous ZTL protein in *gi* mutants^9^ (Supplementary Fig. 9a, lanes 3 and 4). These tests showed that ZTL-LUC protein is diurnally regulated and requires GI for accumulation. We next tested the ability of ZTL-LUC to interact with GI in planta by transient co-expression in *N. benthamiana*. ZTL^WT^-LUC successfully co-immunoprecipitated with GI-HA (Supplementary Fig. 9b, lane 3), while a poorly interacting allele of ZTL (ZTL^G46E^;^9^ failed (ZTL^G46E^-LUC; Supplementary Fig. 9b, lane 4), demonstrating interaction specificity between GI and ZTL–LUC. Third, we tested the ability of ZTL-LUC to reduce levels of the known SCF^ZTL^ substrates, TOC1, and PRR5^5–7^. Co-expression of ZTL with TOC1 or PRR5 significantly reduces the levels of the two substrate proteins when compared to their co-expression with LUC (Supplementary Fig. 9c; compare GFP-PRRn panels, lanes 1 and 3; 4 and 6), but not that of the non-SCF^ZTL^ target, PRR7^7^ (Supplementary Fig. 9c; compare GFP-PRRn panel, lanes 7 and 9). Co-expression of ZTL^WT^-LUC with TOC1 or PRR5 is very similar to authentic ZTL in reducing their levels, when the expression level of the two ZTL proteins is taken into account (Supplementary Fig. 9c; compare GFP-PRRn panels, lanes 2 and 3; 5 and 6). In contrast neither ZTL-LUC nor authentic ZTL has an effect on PRR7 (Supplementary Fig. 9c; compare GFP-PRRn panel, lanes 8 and 9). Taken together these results indicate that the post-translational regulation of ZTL-LUC recapitulates that of authentic ZTL and possesses a similar ability to mediate TOC1 and PRR5 turnover.

**Fig. 3:**
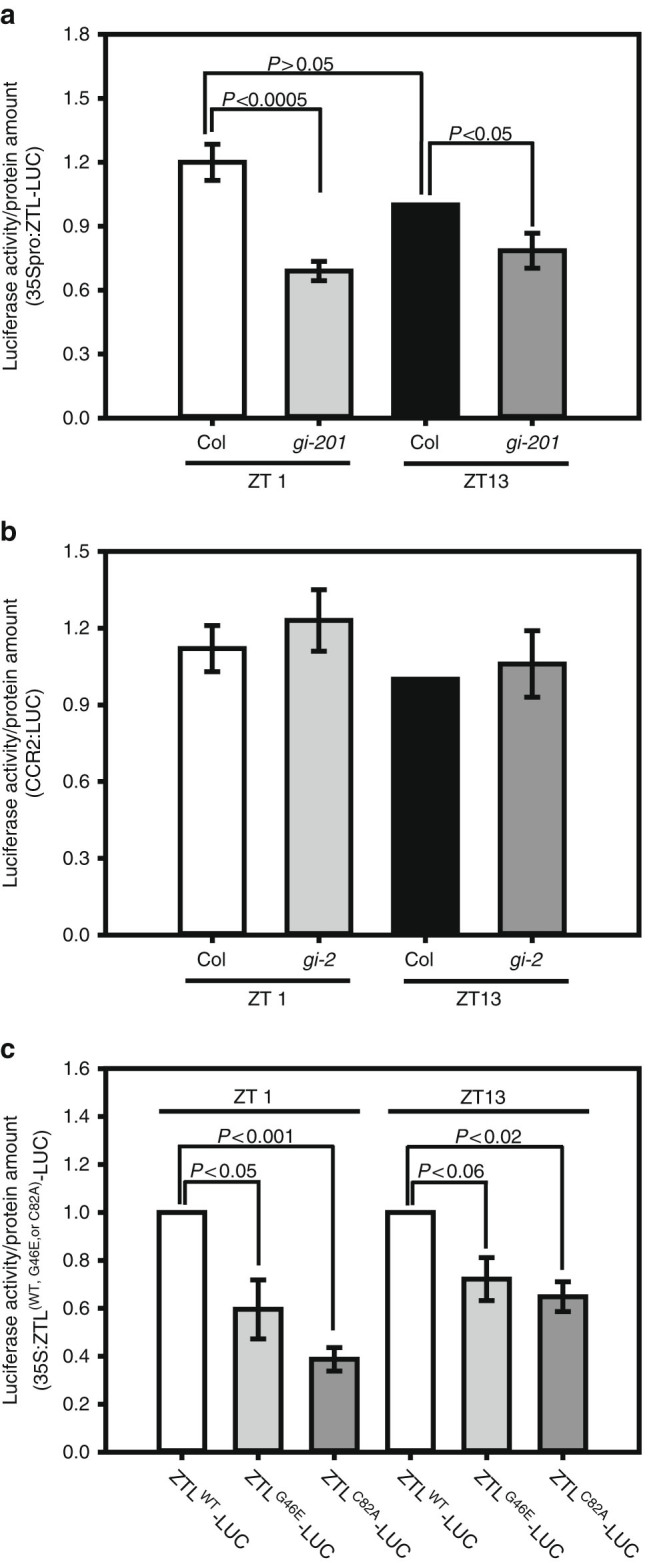
**GI is required in vivo for complete ZTL activity. a** Relative-specific activity of ZTL-LUC at ZT1 and ZT13 in the Col and *gi-201* background. **b** Relative-specific activity of LUC from the *CCR2:LUC* transgene in the Col and *gi-2* backgrounds. **c** Relative-specific activity of ZTL^WT^-, ZTL^G46E^-, and ZTL^C82A^-LUC at ZT1 and ZT13 in the Col background. **a**–**c** Specific activity was determined by the ratio of luminescence (enzyme activity) to LUC protein levels derived from ZTL^WT^-, ZTL^G46E^-, ZTL^C82A^-LUC or LUC protein alone. See “Methods” for details. Data are means ± s.e. of eight (**a**) or four (**b**, **c**) independent samples

We next compared the ratio of ZTL-LUC luciferase activity per ZTL-LUC protein amount in WT and *gi-201* and observed that the specific activity of ZTL-LUC is significantly lower in *gi-201*, relative to WT, at both the minimum (ZT1) and maximum (ZT13) levels of ZTL expression (Fig. 3a; Supplementary Fig. 10a–d). In the WT, ZTL-LUC specific activity was similar at both ZT1 and ZT13. In *gi-201*, ZTL-LUC-specific activity, while consistently lower than WT, was also similar at both time points, indicating that GI is required throughout the circadian cycle to effect fully functional ZTL. Luciferase alone (*CCR2pro-LUC*) expressed in WT and *gi* backgrounds with similar evening phasing as ZTL showed no difference in the level of LUC-specific activity at either time point tested (Fig. 3b).

Additionally, we tested ZTL-LUC-specific activity for ZTL^C82A^-LUC and ZTL^G46E^-LUC variants expressing in the WT GI background (Col). As these variants interact poorly with GI^9^ we reasoned that the respective ZTL^mut^-LUC specific activity would be diminished if GI is needed for maturation. For both ZTL variants at both time points, the variant-specific activity was significantly reduced relative to WT (Fig. 3c; Supplementary Fig. 11). Taken together, results from both in vivo approaches indicate that GI is required for the maturation of fully active ZTL, consistent with the in vitro results (Fig. 2).

### GI forms a ternary complex with HSP90 and ZTL in planta

GI can form tetramers in vitro^47^ so we further tested whether GI occurs in large in vivo complexes in *Arabidopsis*. In non-reducing gels, large GI-containing complexes are detectable and are more enriched in blue (B) and constant white light (LL) than in darkness (D) and red light (R) (Supplementary Fig. 13), consistent with our previous findings^9^. This light-dependent formation of multimeric forms of GI suggests it might function in the context of large complexes in vivo.

The synergistic effect of GI and HSP90 + HSP70 in vitro (Fig. 2e, g) and the co-elution of GI, HSP90, and ZTL during gel filtration (Supplementary Fig. 8) led us to further investigate the composition of the in vivo complexes. HSP90 is a homodimer comprising three well-defined functionally distinct domains that are evolutionarily highly conserved^48^. The N-terminal nucleotide-binding domain (NBD) contains the ATP-binding and hydrolysis region, the middle domain (MD) also participates in ATP hydrolysis, and the C-terminal domain contains the dimerization domain (DD) region^48,49^. Client protein and co-chaperone binding to HSP90 may occur via any of these domains^43,50,51^.

**Fig. 4:**
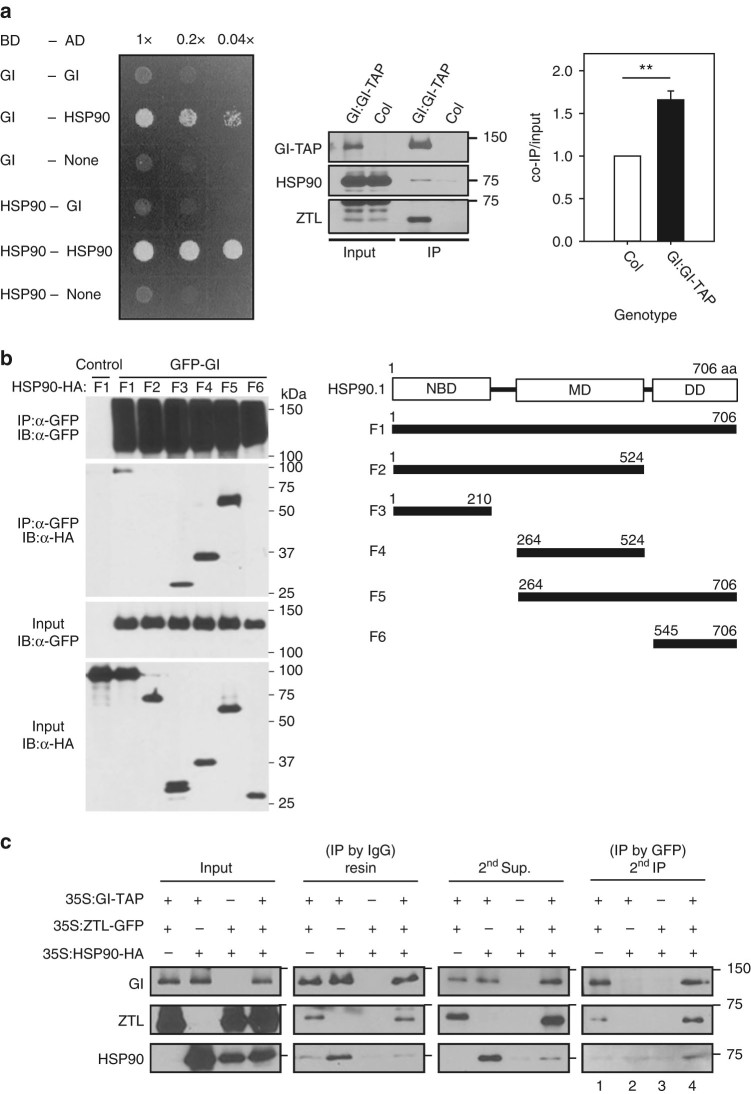
**GI complex formation with ZTL and HSP90 in vivo. a** GI and HSP90 interact directly in yeast (*left panel*) and in planta (*right panel*). Full-length GI and HSP90 protein interaction via yeast two-hybrid was determined by growth on leucine deficient media. Transgenic *Arabidopsis* expressing *GI:GI-TAP* immunoprecipitates with endogenous HSP90 and ZTL (*right panels*). Untransformed Col controlled for non-specific HSP90 interaction. Quantification of HSP90 in GI-TAP immunoprecipitations (IPs) (*far right panel*; mean ± s.e.m.; **P < 0.01) (*n* = 4). **b** HSP90 deletion interactions with GI. Agrobacteria harboring GI-GFP or HSP90 and its respective deletions tagged with 3 × HA were co-infiltrated into *N. benthamiana* leaves. Anti-GFP (IP) were followed by detection of co-immunoprecipitated HSP90-HA and respective deletions. Representative of three trials with similar results. *Right panel*: HSP90 domain structure and respective deletion scheme. *IP* immunoprecipitating antibody, *IB* immunoblot antibody, *NBD* nucleotide-binding domain, *MD* middle domain, *DD* dimerization domain. **c** GI, ZTL, and HSP90 form a tripartite protein complex in planta. Sequential co-immunopreciptations used GI-TAP in the primary IP followed by IP of ZTL-GFP (anti-GFP ab) from the protease-released supernatant (2nd Sup.). The final detection of HSP90-HA (anti HA ab) in lane 4 indicates HSP90-HA associated with ZTL-GFP from the first IP. *N. benthamiana* leaves were triply co-infiltrated with *35S:GI-TAP/35S:HSP90-HA/35S:ZTL-GFP* simultaneously or in all pairwise combinations. Representative of three independent trials

Yeast two-hybrid tests show that full-length GI and HSP90 interact (Fig. 4a). We then tested discrete domain deletions of HSP90 and full-length GI in transient expression assays (Fig. 4b). We detected especially strong co-immunoprecipitation (co-IP) of HSP90 polypeptides when the MD was present either alone or with the HSP90 DD, except when the NBD was also present (Fig. 4b). Since the NBD alone also bound GI it appears that the conformation of an NBD + MD polypeptide blocks GI access. (Fig. 4b). Similarly, an N-terminal portion of GI^NT^ (aa 1–391) also selectively and strongly co-immunoprecipitated with middle-domain containing regions of HSP90, but much more weakly with polypeptides where the NBD is present (Supplementary Fig. 14). Since HSP90 conformation changes with the N-terminal binding of ATP^51,52^, it is possible that a GI–HSP90 interaction is modulated by ATP binding/hydrolysis.

**Fig. 5:**
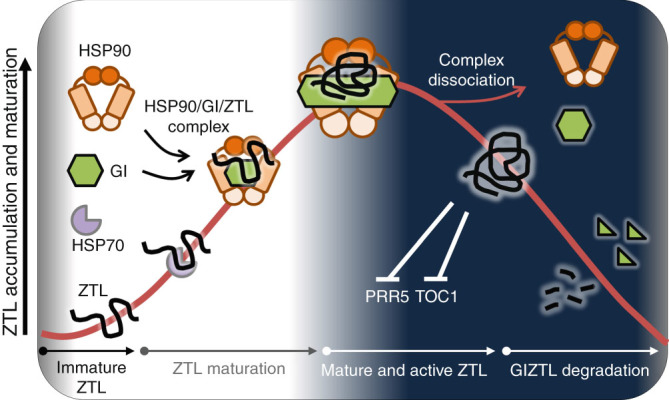
**Post-transcriptional oscillation of ZTL is regulated by GI and HSP90.** Nascent ZTL may be first captured by HSP70 in an early complex, and then transferred to a second complex comprising HSP90 and the co-chaperone GI. Order of ZTL interaction with GI and HSP90 is unknown. GI oligomerization and ZTL interaction is enhanced in the light, which may increase the binding capacity with ZTL and HSP90. Matured/active ZTL dissociates from the complex, to form SCF^ZTL^ which ubiquitylates PRR5 and TOC1. During the dark period ZTL is degraded together with GI

In plants expressing *GI:GI-TAP* both ZTL and HSP90 can be co-immunoprecipitated (Fig. 4a). To establish that all three proteins can exist within one complex we performed sequential co-immunoprecipitations using GI-TAP in the primary IP followed by an IP of ZTL-GFP and detection of HSP90-HA in the final (2^nd^) IP. The presence of enriched levels of HSP90 in the 2nd IP (Fig. 4c, lane 4; Supplementary Fig. 12) is only possible if HSP90 is associated with the ZTL-GFP that was initially complexed with GI-TAP. These results show the presence of a ternary complex in planta, supporting the gel filtration (Supplementary Fig. 8) and synergistic in vitro folding results (Fig. 2g). Taken together, these findings indicate that GI acts as a co-chaperone with HSP90 to mature ZTL into its fully functional form (Fig. 5).

## Discussion

Proteostasis is the sum total of processes involving the synthesis, folding, and maturation, and turnover of polypeptides in the cell. Molecular chaperones are essential in cellular proteostasis, primarily in promoting and regulating the correct folding and maturation of their client proteins^27^. HSP90 is one of the most ubiquitous chaperones in eukaryotes, playing a central role in all aspects of cell regulation^49,53,54^. The extremely wide-range of HSP90 clients requires mechanisms to determine client specificity and this is achieved, in part, through the complexing of particular co-chaperones and other adaptors with HSP90^31,50^.

Our previous study established ZTL as a client of HSP90 but the question of specificity was unresolved^32^. Here we have identified GI as a co-chaperone that interacts with HSP90 to specify ZTL as a client of the larger complex. HSP90 and HSP70 together can restore 35% of the enzyme activity of denatured ZTL-GST but the inclusion of GI^N^, representing more than 70% of the full-length polypeptide, synergistically restores ZTL-GST to 100% activity (Fig. 2e). Mutant ZTL-GST (ZTL^C82A^), which greatly diminishes the ZTL–GI interaction, also renatures to only ca. 35% activity in the presence of HSP70/HSP90 alone, but addition of GI^N^ has little effect (Fig. 2f).

Additionally, the inability of GI alone to substantially restore ZTL-GST activity without HSP90/HSP70 suggests it may act primarily as a holdase to position ZTL correctly in the presence of HSP90. The existence of a ZTL-GI-HSP90 complex in vivo (Fig. 4c) supports this notion. We suggest that GI facilitates steps after the HSP70-to-HSP90 shuttling of the client. HSP70 typically acts early in the folding process, by binding to short, five-residue long hydrophobic stretches of amino acids of the client^43^. This complex then hands off the nascently folded client substrate to HSP90 for final conformational maturation and stabilization (Fig. 5).

The strong, synergistic promotion of ZTL-GST to full activity through the addition of GI^N^ to an HSP70/HSP90 complex is similar to previous reports of the enhancement of chaperone activity (e.g., HSP90) when co-chaperones are included in the reaction^55–57^. This may occur through a change in the inherent ATPase activity of HSP90, by helping direct HSP90 to specific client proteins or through unknown mechanisms. In vivo, ZTL-LUC-specific activity was reduced either in the absence of GI (Fig. 3a) or when interaction with GI was diminished by ZTL mutation (Fig. 3c). This, too, is consistent with increased recruitment of clients to HSP90 by co-chaperones. Because ZTL protein abundance tracks GI levels so closely^9^ it is likely that the oscillation in GI protein accumulation is the determinant in ZTL maturation. As well, the specific activity of ZTL-LUC is very similar in the WT at both ZT1 and ZT13 (Fig. 3a), when the ZTL and GI levels are both near their lowest (ZT1) and highest (ZT13) levels. This also supports the notion that GI levels control the extent of ZTL maturation, such that in the absence of GI ZTL maturation still occurs but at a slower, less efficient rate.

The entry point of known co-chaperones to the HSP90 chaperone cycle can vary greatly. For example, the mammalian co-chaperone Hop connects HSP90 and HSP70 in a multi-chaperone complex, where it facilitates the transfer of client proteins from an early HSP70 complex (HSP70-HSP40) to an intermediate complex (HSP70-HSP90)^48^. Like Hop, other co-chaperones such as CDC37, and SGT1 and RAR1 in plants, also act to deliver the client to HSP90, often slowing the rate of ATPase activity inherent in HSP90 action^50,52,58,59^. Other co-chaperones (e.g., Sba1/p23; Aha1) act later in the maturation process and are not explicitly involved in client delivery to the HSP90 complex^50,52^. Further work is necessary to determine the precise point of GI entry to the HSP90 chaperone cycle, but our findings support participation in an early step where GI may either first bind ZTL for proper HSP90 access or co-bind ZTL with HSP90.

Different co-chaperones bind to different portions of HSP90 and no conserved co-chaperone-binding domain has been identified^51^. This is consistent with the different interaction regions of GI and HSP90 identified in the co-immunoprecipitation interaction tests (Fig. 4b; Supplementary Fig. 14). The MD domain of HSP90 is needed to interact with full-length GI and the GI N-terminus, but the interaction is greatly weakened when the N-terminal NBD is present. Since HSP90 conformation changes with the N-terminal binding of ATP^51,52^, it is possible that a GI–HSP90 interaction is modulated by ATP binding/hydrolysis.

We have now identified a mechanism that can explain the far-reaching effects of GI on plant physiology, metabolism, and development. Given the diversity of phenotypes observed in *gi* mutants^15^, GI is likely to effect the maturation of a wide range of client proteins. This role could be similar to that of yeast co-chaperone p23, which acts together with, and separately from, HSP90 in a wide-ranging global network of chaperone activity^60,61^. It is also likely that additional factors complex with GI, separately or together with HSP90, to confer specificity to the roles GI has in plant biology. Additionally, the strong diel and circadian oscillations in GI levels^9,18,23^ now implicate the circadian clock in a chaperone surveillance system that helps to globally regulate proteostasis in vascular plants.

## Methods

### Plasmid construction and plant materials

The constructs of full-length ZTL(WT), ZTL(G46E), and ZTL(C82A) fused with LUC were prepared using the Gateway system (Invitrogen). Entry clones for ZTL, ZTL(G46E), ZTL(C82A) fused with LUC were generated by transferring the ZTL-LUC fragment, from the plasmids generated by recombination reaction of ZTL(WT), ZTL(G46E), and ZTL(C82A) entry clones with the Gateway version of pOmegaLUC_SK+, into the pCR-CCD-F vector^22,62^. The final constructs for the generation of *Arabidopsis* transgenic plants were established by the LR recombinase reaction using each entry clone and pMDC32^63^. A *35S:ZTL(WT)-LUC* transgenic line expressing stable ZTL-LUC transcript levels when crossed with *gi-201* was selected. *35S:ZTL(G46E)-LUC* and *35S:ZTL(G46E)-LUC* transgenic lines were chosen, based on their comparable expression of ZTL-LUC to that in *35S:ZTL(WT)-LUC*. *GI:GI-TAP* and *GI:GI-HA* ^23^, *CCR2-LUC*+ and *gi-2CCR2-LUC*+^64^ have been described previously. The GFP-GI and GFP-GI^NT^ constructs were generated by LR recombination with pENTR2B-GI, pENTR2B-GI^NT^, and pMDC45-GFP binary vector, respectively. Supplementary Table 2 lists primers used in construction of these plasmids and those in the following sections.

### Recombinant protein expression

cDNA of GI^N^ (1–858 aa) and GI^C^ (920–1173 aa) were cloned into the donor vector (pDONR-zero) and subsequently moved into the gMAL c2B and gRSETA vector to produce recombinant protein with an N-terminal maltose-binding protein (MBP-) and 6xHis tag (His-), respectively, using the recombination-based Gateway cloning system (Invitrogen), according to the manufacturer’s instructions. Site-directed mutagenesis for MBP-ZTL^C82A^ and GST-ZTL^C82A^ were generated using *pMalc2X::ZTL* (for MBP-ZTL^WT^)^32^ and *pGEX-KG::ZTL* (GST-ZTL^WT^)^65^ as a template for Pfu Turbo DNA polymerase (QuickChange Site-Dircted Mutagenesis Kit; Stratagene), according to the manufacturer’s instructions. The *pET28a::HSP90.2* (His-HSP90), *pMalc2X::ZTL* (MBP-ZTL), and *pET41a::HSP70* (GST-HSP70) were prepared as described previously^32,66^. The plasmids without *pMALc2X* (for produce MBP fusion protein only), *pGEX5X-1* (for GST only), *pET28a::HSP90.2* (At5G56030), and *pMalc2X::ZTL* were transformed into *E. coli* BL21 (DE3) pLysS for recombinant protein expression. The *E. coli* transformants were grown at 37 °C (OD600 = 0.8) and His-HSP90^32^ and GST-HSP70 (EU541356)^66^ were induced by 0.5 mM isoprophyl-1-thio-β-_D_-galactopyranoside (IPTG) and MBP, MBP-GI^N^, MBP-GI^C^, His-GI^N^, His-GI^C^, MBP-ZTL^WT^, MBP-ZTL^C82A^, GST, GST-ZTL^WT^, and GST-ZTL^C82A^ by 1 mM IPTG. After 3 h at 30 °C cultures were harvested, resuspended in 1× PBS and incubated for 20 min in the presence of 1% (v/v) Triton X-100 followed by disruption by sonication. For GST-ZTL^WT^ and GST-ZTL^C82A^, cells were incubated in 1% (v/v) of sodium lauroyl sarcosinate (sarkosyl) followed by incubation in 1% (v/v) Triton X-100 before disruption by sonication^42^. After centrifugation, supernatants of His-tagged proteins were loaded onto a Ni-NTA Sepharose CL-6B affinity column (Peptron), those of GST-fusion proteins onto a Glutathione Sepharose 4B Fast Flow (GE healthcare), and MBP-fusion proteins onto an Amylose resin (New England Biolabs). This was followed by washing using cold 1× PBS for GST-fused and MBP-fused proteins and 50 mM imidazole for His-tagged proteins, GST-fused proteins were eluted by 10 mM reduced glutathione, MBP-fused proteins by 10 mM maltose, and His-tagged proteins by 200 mM imidazole. HSP70 was eluted by thrombin digestion to cleave GST fusion protein from the resin. All recombinant proteins were dialyzed against 50 mM HEPES (pH 7.5).

### Holdase chaperone assay

The holdase chaperone activity of MBP-GI^N^/GI^C^ was assayed by measuring its capacity to suppress heat-induced aggregation of *Arabidopsis* MDH, as a model substrate or MBP-ZTL^WT^ and MBP-ZTL^C82A^, as an authentic GI substrate, prepared as described previously^32,66^. Aggregation of the substrate was monitored in the absence or presence of MBP-GI^N^/GI^C^ with various molar ratios under heat denaturation at 45 °C for 15 min by measuring the turbidity at 340 nm using a Beckman DU-800 spectrophotometer (Beckman Coulter) attached to a thermostatic cell holder assembly. The light scattering values of each substrate alone at the endpoint (15 min) of incubation was set to 100%, and the absorbance value of each treatment expressed relative to it. All holdase assays were performed in 40 mM HEPES (pH 7.5) with HSP70 and BSA used as positive and negative controls, respectively.

### Foldase chaperone assay

Foldase chaperone activity was assayed by measuring the capacity to refold chemically denatured G6PDH as a model substrate (Sigma)^66^. G6PDH (1 μM) was denatured in 4 M guanidine-HCL (Gn-HCl) for 2.5 h at room temperature and refolded in renaturation buffer (50 mM Tris-HCl, pH 7.5, 10 mM ATP, 10 mM KCl, and 2.5 mM MgCl_2_) in the absence (spontaneous refolding) or presence of GroEL^40^ (as a positive control, Takara), MBP (as a negative control), MBP-GI^N^, or MBP-GI^C^. Refolded G6PDH was monitored by measuring the formation of NADPH at Abs_340_ in assay buffer (50 mM Tris-HCl, pH 7.5, 1 mM NADP) containing 2 mM glucose-6-phosphate (Sigma) as a substrate. The activity was calculated relative to native G6PDH activity (set to 100%). To examine ZTL refolding activity, we developed a foldase chaperone assay protocol using a substrate (ZTL) translationally fused to glutathione-S-transferase (GST) based on CDNB^42^. Recombinant GST, GST-ZTL^WT^, and GST-ZTL^C82A^ (2 μM) were denatured in 100 mM potassium phosphate (pH 6.5) at 45 °C for 3 h. Denatured proteins (33.3 nM) were immediately incubated in the absence (spontaneous refolding) or presence of various combinations of candidate chaperones (His-GI^N^ or His-GI^C^ in the absence or presence of HSP90/HSP70) using a combined renaturation and GST assay buffer (100 mM potassium phosphate, pH 6.5, 10 mM ATP, 10 mM KCl, 2.5 mM MgCl_2_, 2 mM CDNB and 2 mM GSH). GST activity was followed by measuring the formation of GS-DNB conjugate (a reaction product of GST) at Abs_340_, and was expressed relative to the activity of undenatured GST, GST-ZTL^WT^, or GST-ZTL^C82A^ (set to 100%). Both foldase assays were conducted at 25 °C with a Beckman DU-800 spectrophotometer attached to a thermostatic cell holder assembly.

### GI oligomerization

Ten-day-old *GI:GI-HA* plants grown in 12L/12D conditions were harvested at ZT6 and ZT18 for sampling in white light (L) and darkness (D), respectively. The plants were also harvested at ZT18 under blue (B), red (R), and constant white light (LL). Total proteins were homogenized in extraction buffer containing 100 mM Tris-HCl (pH 7.5), 150 mM NaCl, 0.5% (v/v) NP-40, 1 mM EDTA and protease inhibitors (1 mM PMSF, 5 μg ml^−1^ leupeptin, 1 μg ml^−1^ aprotinin, 1 μg ml^−1^ pepstatin, 5 μg ml^−1^ antipain, 5 μg ml^−1^ chymostatin, 2 mM Na_2_VO_3_, 2 mM NaF, and 50 μM MG132) in the absence (for non-reducing SDS–PAGE) or presence of DTT (3 mM; for reducing SDS–PAGE). Supernatant was recovered after centrifugation at 10,000*g* for 10 min at 4 °C. The proteins were separated on 10% non-reducing SDS–PAGE (without β-mercaptoethanol in loading buffer) and reducing SDS–PAGE (with β-mercaptoethanol), and then analyzed by immunoblot analysis to detect GI-HA^23^.

### Gel filtration

Ten-day-old *Arabidopsis GI:GI-HA*, *35S:GI-HA*, and *gi-2 ztl103* seedlings were harvested as indicated and homogenized in extraction buffer without DTT as above. Supernatant was recovered after centrifugation at 10,000*g* for 10 min at 4 °C and filtered through a 0.45 μm disk-filter (Advantec). The resulting supernatant (1 mg protein) was eluted through a Superdex 200 HR 10/30 column pre-equilibrated with elution buffer (50 mM Tris-HCl (pH 7.5), 100 mM NaCl, and 0.02% sodium azide) at a flow-rate of 0.5 ml min^−1^. The eluted proteins were immediately precipitated with 10% (v/v) trichloroacetic acid for 10 min on ice, washed by 100% acetone twice and dissolved in urea/SDS buffer. The fractions were separated on SDS–PAGE and analyzed by immunoblot analysis to detect GI-HA, ZTL, and HSP90^23,32^.

### Mass spectrometry

Recombinant MBP-GI variants (MBP-GI^N^ and MBP-GI^C^) were identified by Matrix Assisted Laser Desorption/Ionization Time of Flight/Time of Flight (MALDI-TOF/TOF) Mass Spectroscopy (MS)^67^. Purified recombinant MBP-GI^N^ and MBP-GI^C^ proteins from *E. coli* were separated on 10% SDS–PAGE and stained with Coomassie-brilliant blue. The bands were excised and digested by trypsin (50 ng ml^−1^). Peptides extraction was done twice with one volume of acetonitrile (ACN)/water/CF_3_COOH (66:33:0.1, v/v/v) solution, sonicated, centrifuged, and dried using speed vacuum. Dried peptides were dissolved in 50% (v/v) ACN/0.1% (v/v) trifluoroacetic acid solution. The solution was carefully spotted onto the MALDI-TOF/TOF-MS target plate and analyzed on an ABI 4800 Plus TOF-TOF Mass Spectrometer (AB Sciex). Spectral data were searched using Mascot (version 2.3.0; Matrix Science) and search criteria were—single missing pick, oxidation of methionines, and carbamidomethylation of cysteines. A statistically significant value is *p* = 0.05 to search individual peptide ions score.

### Yeast two-hybrid

Yeast two-hybrid assay was performed using LexA-based assay system (DupLEX-A; OriGene Technology). The final bait and prey constructs were established by the LR recombinase reaction using each entry clone (HSP90^(32)^ and GI^(9)^) and Gateway versions of pGilda and pOST4-5^(9)^. Sets of constructs were co-transformed into EGY48 (ura3, his3, trp1, leu:6 LexAop-LEU2) containing lacZ reporter plasmid pSH18-34. Yeast transformants were selected on glucose-based synthetic minimal medium (SD; 0.67% yeast nitrogen base, 2% glucose (wt/vol), and amino acid dropout solution) deficient in histidine, tryptophan, and uracil (-HIS-TRP-URA) and protein interaction tests were performed after protein induction on galactose and raffinose-based SD-HIS-TRP-URA media.

### In vivo maturation assay for LUC and ZTL-LUC

*Arabidopsis* seedlings were grown for 10 d in a 12-h L/12-h D photoperiod and then harvested at ZT1 (1 h after lights on) and ZT13 (1 h after lights off). Protein extracts were prepared using extraction buffer (50 mM Tris-Cl, pH 7.5, 1 mM EDTA, 1 mM dithiothreitol, 1 mM phenylmethylsulfonyl fluoride, 5 μg ml^−1^ leupeptin, 1 μg ml^−1^ aprotinin, 1 μg ml^−1^ pepstatin, 5 μg ml^−1^ antipain, 5 μg ml^−1^ chymostatin, 50 μM MG132, 50 μM MG115, and 50 μM ALLN). Protein extracts from *35S:ZTL-LUC* (Col) harvested at ZT13 were diluted with untransformed Col protein extracts by a factor of 1–3 to obtain similar LUC activity levels for *35S:ZTL-LUC* in the Col and *gi-201* backgrounds. Luciferase activity of ZTL-LUC from protein extracts was measured using Luciferase assay system (Promega) and 96-well dual-injection luminometer (Centro LB960; Berthold Technologies), according to the manufacturer’s instructions and normalized with adenosine kinase (ADK) protein levels, which was determined by SDS–PAGE and subsequent immunodetection using anti-ADK antibody (1:40,000)^9^. Linear dynamic ranges for luciferase activity was confirmed by using serial diluted samples with the *35S:ZTL-LUC* (Col) harvested at ZT13.

Protein levels of ZTL-LUC were determined by immunoblot analysis from TCA concentrated proteins from the same protein extracts using purified anti-LUC antibody, and further normalized to ADK protein levels. Specific activity of LUC was determined by the ratio of luciferase activity (normalized to ADK protein levels) to the level of LUC protein levels (normalized to ADK protein levels). For each biological trial the value for each non-ZT13 Col WT sample (Fig. 3a, b) were determined as a relative value calculated by normalization to the reference samples (Col_ZT13, set to 1 for each trial). The same was done for Fig. 3c except normalization was to ZT1 or ZT13 among the different genotypes. Purified anti-LUC antibody was generated from anti-LUC antibody (Sigma, L0159) by immunoaffinity purification against bacterially expressed LUC immobilized to poly(vinylidene difluoride) membrane by standard techniques.

### Immunoprecipitation analyses

10-d-old *Arabidopsis* seedling were grown in 12-h L/12-h D photoperiod and harvested at ZT13 on MS plates was subjected to in vivo protein cross-linking^22^. Immunoprecipitation was performed using anti-PAP antibody (Sigma, P1291) and protein visualization was performed by SDS–PAGE separation, followed by immunodetection using anti-HSP90 (1:50,000), anti-ZTL (1:500), and anti-PAP antibody (1:1000)^9^. Uncropped versions of these and other gels and blots shown in the figures and Supplementary Information are found in Supplementary Fig. 15.

### Sequential immunoprecipitation

GI-TAP, HSP90-HA, and ZTL-GFP were constitutively co-expressed in *N. benthamiana* in all pairwise and triple combinations by *Agrobacterium* infiltration. Proteins were extracted and incubated with IgG-agarose (Sigma) for 1 h at 4 °C^9^. The harvested immune complexes were washed three times with buffer and GI-TAP complexes were released from IgG beads using 3C protease (2 units, Precision Protease, GE Healthcare Life Science) for 3 h at 4 °C. Supernatants were incubated for 2 h at 4 °C with protein A agarose (Invitrogen), which had been pre-incubated with anti-GFP mouse monoclonal antibody (Molecular Probes) at 4 °C. The resulting immune complexes (ZTL-GFP) were washed four times, resuspended in SDS–PAGE sample buffer, briefly heated (93 °C, 5 min) and subjected to SDS/PAGE and immunoblotting.

### Co-immunoprecipitation assay

Agrobacteria containing HA-tagged GI full length (FL), GI^N^, or GI^C^ were co-infiltrated with GFP-tagged wild-type (WT) ZTL or ZTL^C82A^ into 3–4 week old *N. benthamiana* leaves. Samples were collected 3 days after infiltration, tissue ground in liquid nitrogen and protein extraction performed^68^. Immunoprecipitation was performed with HA antibody (Roche; 3F10) and immunoblots were probed with anti-HA (1:2000) and anti-GFP antibodies (Abcam; ab6556, 1:5000).

### In planta protein stabilization assay

Approximately 4.5 × 10^5^ protoplasts from 4-week-old *Arabidopsis thaliana gi-201* were used to transform with appropriate vectors^62^. After transfection protoplasts were incubated under dim white light for 26 h at 22 °C and then collected by centrifugation at 845 g for 5 s. Supernatants were removed gently and the remaining protoplasts were frozen in liquid nitrogen followed by protein extraction using protein extraction buffer (100 mM Tris-Cl, pH 7.5, 150 mM NaCl, 1 mM EDTA, 0.5% NP-40, 1 mM DTT, 1 mM PMSF, 5 μg ml^−1^ leupeptin, 1 μg ml^−1^ aprotinin, 1 μg ml^−1^ pepstatin, 5 μg ml^−1^ antipain, 5 μg ml^−1^ chymostatin, 2 mM NaVO_3_, 2 mM NaF, 50 µM MG132, 50 µM MG115, 50 µM ALLN). Proteins were separated on 8% of SDS–PAGE gel and detected by HA and GFP antibodies as described above.

### Data availability

The authors declare that all data supporting the findings of this study are available within the manuscript and its Supplementary Information files are available from the corresponding author upon request.

## Electronic Supplementary Material

Supplementary Information Supplementary Figures, Supplementary Tables and Supplementary References
