# Supplementary material for: GIGANTEA is a co-chaperone which facilitates maturation of ZEITLUPE in the Arabidopsis circadian clock
Source: Nat Commun. 2017 Feb 23;8:3. doi: 10.1038/s41467-016-0014-9 (PMC5431898; doi:10.1038/s41467-016-0014-9)
Supplement: Supplementary file 1 — Supplementary Figures, Supplementary Tables and Supplementary References [file 41467_2016_14_MOESM1_ESM.pdf]

a

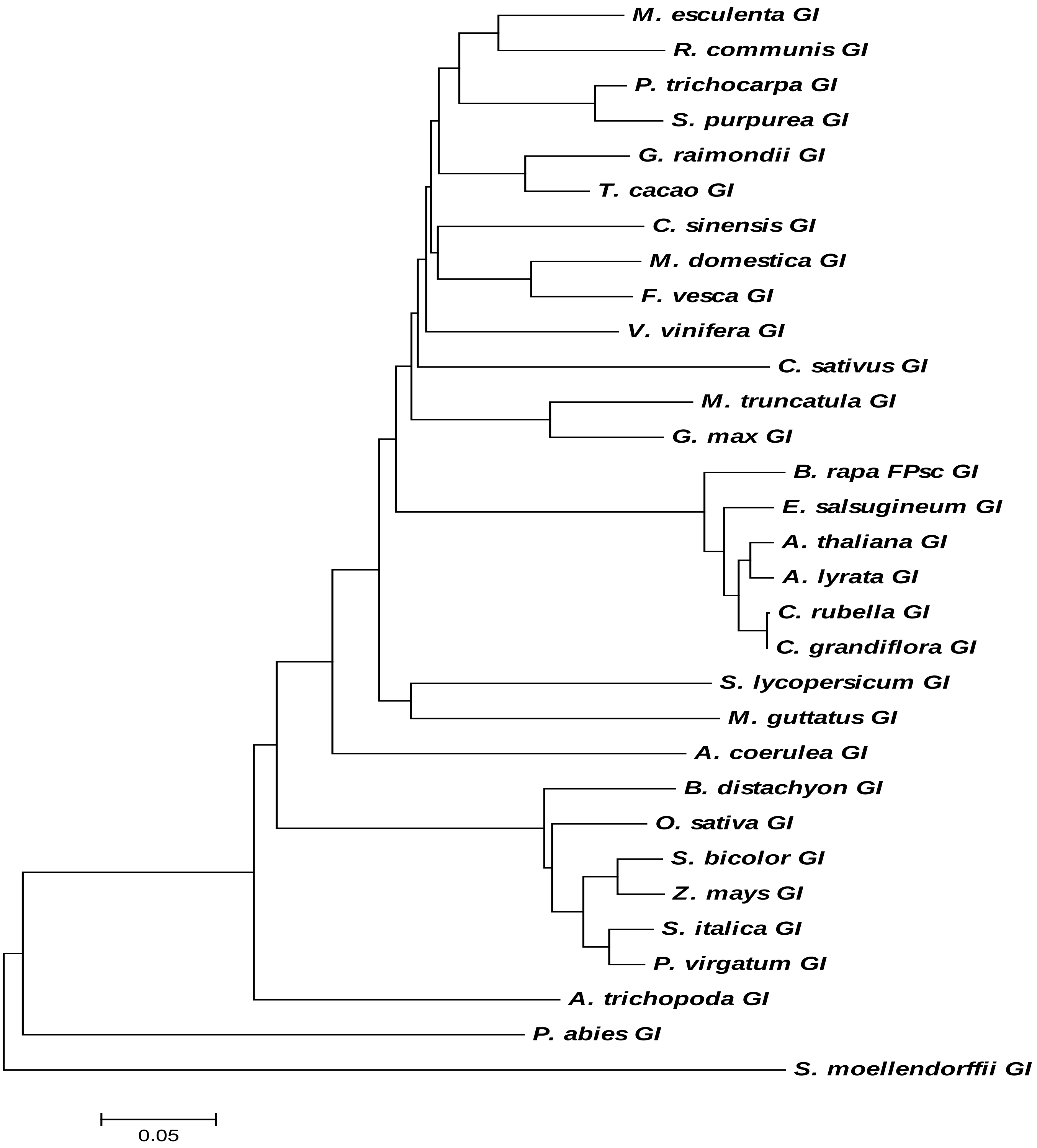
M. esculenta GI
R. communis GI
P. trichocarpa GI
S. purpurea GI
G. raimondii GI
T. cacao GI
C. sinensis GI
M. domestica GI
F. vesca GI
V. vinifera GI
C. sativus GI
M. truncatula GI
G. max GI
B. rapa FPsc GI
E. salsugineum GI
A. thaliana GI
A. lyrata GI
C. rubella GI
C. grandiflora GI
S. lycopersicum GI
M. guttatus GI
A. coerulea GI
B. distachyon GI
O. sativa GI
S. bicolor GI
Z. mays GI
S. italica GI
P. virgatum GI
A. trichopoda GI
P. abies GI
S. moellendorffii GI
0.05

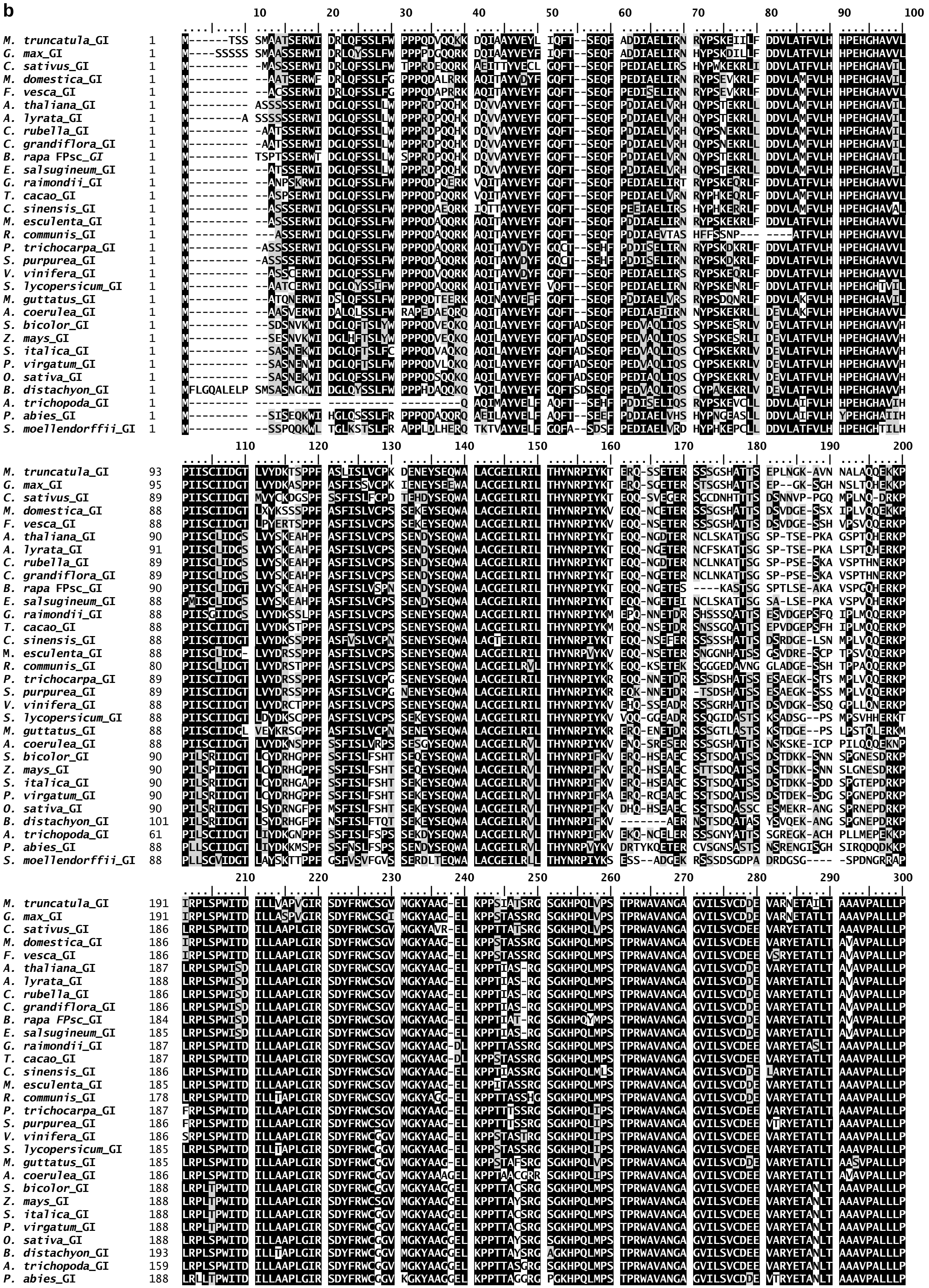
b
10 20 30 40 50 60 70 80 90 100
M. truncatula_GI 1 M------TSS SMAATSERWI DRLQFSSLFW PPPQDVQQKK DQIAAYVEYL IQFT--SEQF ADDIAELIRN RYPSKEIILF DDVLATFVLH HPEHGHAVVL
G. max_GI 1 M----SSSSS SMAASSERWI DRLQYSSLFW PPPPDGQQRK DQIAAYVEYF IQFT--SEQF ADDIAELIRN HYPSKDILLF DDVLATFVLH HPEHGHAVVL
C. sativus_GI 1 M--------- -MASSSERWI DRLQFSSLFW TPPRDEQQRK AEITTYVECL GQFT--SEQF PEDIAELIRS HYPWKEKRLI DDVLATFVLH HPEHGHAVIL
M. domestica_GI 1 M--------- --AATSERWF DRLQFSSLFG PPPQDALRRK AQITAYVDYF GQFT--SEQF PEDIAELIRN RYPSEVKRLF DDVLAMFVLH HPEHGHAVIL
F. vesca_GI 1 M--------- --ACSSERWI DRLQFSSLFG PPPQDAPRRK AQITAYVEYF GQFT--SEQF PEDISELIRN RYPSEVKRLF DDVLAMFVLH HPEHGHAVIL
A. thaliana_GI 1 M--------- ASSSSSERWI DGLQFSSLLW PPPRDPQQHK DQVVAYVEYF GQFT--SEQF PDDIAELVRH QYPSTEKRLL DDVLAMFVLH HPEHGHAVIL
A. lyrata_GI 1 M--------A SSSSSSERWI DGLQFSSLLW PPPRDPQQHK DQVVAYVEYF GQFT--SEQF PDDIAELVRH QYPSTEKRLL DDVLAMFVLH HPEHGHAVIL
C. rubella_GI 1 M--------- -AATSSERWI DGLQFSSLLW PPPRDPQQHK DQVVAYVEYF GQFT--SEQF PDDIAELVRS QYPSNEKRLL DDVLAMFVLH HPEHGHAVIL
C. grandiflora_GI 1 M--------- -AATSSERWI DGLQFSSLLW PPPRDPQQHK DQVVAYVEYF GQFT--SEQF PDDIAELVRH QYPSNEKRLL DDVLAMFVLH HPEHGHAVIL
B. rapa FPsc_GI 1 M--------- TSPTSSERWT DGLQFSSLLW SPPRDPQQHK DQVVAYVEYF GQFT--SEQF PDDIAELVRN QYPSTEKRLL DDVLAMFVLH HPEHGHAVIL
E. salsugineum_GI 1 M--------- --ATSSERWI DGLQFSSLLW PPPRDPQQHK DQVVAYVEYF GQFT--SEQF PDDIAELVRH QYPSTEKRLL DDVLAMFVLH HPEHGHAVIL
G. raimondii_GI 1 M--------- --ANPSKRWI DGLQFSSLFW PPPQDPQERK VQITAYVEYF GQFT--SEQF PEDIAELIRT RYPSKEQRLF DDVLATFVLH HPEHGHAVVL
T. cacao_GI 1 M--------- --ASPSERWI DGLQFSSLFW PPPQDPQQRK VQITAYVEYF GQFT--SEQF PEDIAELVRN RYPHKEQRLF DDVLAMFVLH HPEHGHAVVL
C. sinensis_GI 1 M--------- --ASSSERWI DGLQFSSLFW PPPQDAEQRK IQTTAYVEYF GQFT--SEQF PEEIAELIRS HYPHKERRLF DDVLAMFVLH HPEHGHAVAL
M. esculenta_GI 1 M--------- --ASSSERWI DGLQFSSLFW PPPQDAQQRK AQITAYVEYF GQFT--SEQF PDDIAELIRN RYPSKEKRLF DDVLATFVLH HPEHGHAVVL
R. communis_GI 1 M--------- ---ASSERWI DGLQFSSLFW PPPQDAQQRK AQITAYVEYF GQFT--SEQF PDDIAEVTAS HFFSSNP--- ----ATFVLH HPEHGHAVVL
P. trichocarpa_GI 1 M--------- -ASSSSERWI DGLQFSSLFW PPPQDAQQRK AQITAYVDYF GQCT--SEHF PDDISELIRN RYPSKDKRLF DDVLATFVLH HPEHGHAVVL
S. purpurea_GI 1 M--------- -ASSSSERWI DGLQFSSLFW PPPQDAQQRK AQITAYVDYF GQCT--SEHF PDDISELIRN RYPSKDKRLF DDVLATFVLH HPEHGHAVVL
V. vinifera_GI 1 M--------- --ASSCERWI DGLQFSSLFW PPPQDVQQRK AQITAYVEYF GQFT--SEQF PEDIAELIRS RYPSKEQRLF DDVLATFVLH HPEHGHAVVL
S. lycopersicum_GI 1 M--------- --AATCERWI DGLQYSSIFW PPPQDAQQRK AQITAYVEYF VQFT--SEQF PEDIAELIRN RYPSKENRLF DDVLATFVLH HPEHGHTVIL
M. guttatus_GI 1 M--------- --ATQNERWI DSLQFSSLFW PPPQDTEERK AQINAYVEFF GQFT--SEQF PDDIAELVRS RYPSDQNRLF DDVLAKFVLH HPEHGHAVIL
A. coerulea_GI 1 M--------- --AASVERWI DALQLSSLFW RAPEDAEQRQ AQITAYVEYF GQFT--SEQF PEDIAEIIRN NYPSKEKRLL DEVLAKFVLH HPEHGHAVVL
S. bicolor_GI 1 M--------- --SDSNVKWI DGLQFTSLYW PPPQDVEQKQ AQILAYVEYF GQFTADSEQF PEDVAQLIQS SYPSKESRLV DEVLATFVLH HPEHGHAVVH
Z. mays_GI 1 M--------- --SESNVKWI DGLHFTSLYW PPPQDVEQKQ AQILAYVEYF GQFTADSEQF PEDVAQLIQS SYPSKESRLI DEVLATFVLH HPEHGHAVVH
S. italica_GI 1 M--------- --SASNEKWI DGLQFTSLFC PPPQDVAQKQ AQILAYVEYF GQFTADSEQF PEDVAQLIQS CYPSKEKRLV DEVLATFVLH HPEHGHAVVH
P. virgatum_GI 1 M--------- --SASNENWI DGLQFTSLFW PPPQDVLQKQ AQILAYVEYF GQFTADSEQF PEDVAQLIQS CYPSKEKRLV DEVLATFVLH HPEHGHAVVH
O. sativa_GI 1 M--------- --SASNEKWI DGLQFSSLFW PPPQDSQQKQ AQILAYVEYF GQFTADSEQF PEDIAQLIQS CYPSKEKRLV DEVLATFVLH HPEHGHAVVH
B. distachyon_GI 1 MFLGQALELP SMSASNGKWI DGLQYSSLFW PPPHDAQQKQ VQILAYVEYF GQFTSDSEQF PEDVAQLIQS CYPAKEKRLV DEVLATFVLH HPEHGHAVVH
A. trichopoda_GI 1 M--------- ---------- ---------- ---------Q AQIMAYVELF AQFT--SEHF PDDISELIQS RYPSKEVCLL DDVLAIFVLH HPEHGHAVIH
P. abies_GI 1 M--------- --SISEQKWI HGLQSSSLFR PPPQDAQQRQ AEILAYVELF AQFT--SEEF PDDIAELVHS HYPNGEASLL DDVLAIFVLH YPEHGHAIIH
S. moellendorffii_GI 1 M--------- --SSPQQKWL TGLKSTSLFR APPLDLHERQ TKTVAYVELF GQFA--SDSF PEDIAELVRD HYPHKEPCLL DDVLATFVLH HPEHGHTILH
110 120 130 140 150 160 170 180 190 200
M. truncatula_GI 93 PIISCIIDGT LVYDKTSPPF ASLISLVCPK DENEYSEQWA LACGEILRIL THYNRPIYKT ERQ-SSETER SSSGSHATTS EPLNGK-AVN NALAQQEKKP
G. max_GI 95 PIISCIIDGT LVYDKASPPF ASFISSVCPK IENEYSEEWA LACGEILRIL THYNRPIYKT ERQ-SGETER STSGSHATTS EP--GK-SGH NSLTQHEKKP
C. sativus_GI 89 PIISCIIDGT MVYCKDGSPF SSFISLFCPD TEHDYSEQWA LACGEILRIL THYNRPIYKT EQQ-SVEGER SGCDNHTTTS DSNNVP-PGQ MPLNQ-DRKP
M. domestica_GI 88 PIISCIIDGT LXYKSSSPPF ASFISLVCPS SEKEYSEQWA LACGEILRIL THYNRPIYKV EQQ-NCETER SSGGSHATTS DSVDGE-SSX IPLVQQEKKP
F. vesca_GI 88 PIISCIIDGT LPYERTSPPF ASFISLVCPS SEKEYSEQWA LACGEILRIL THYNRPIYKV EQQ-NSETER SSSGSHATTS DSVDGE-SSH VPSVQQERKP
A. thaliana_GI 90 PIISCLIDGS LVYSKEAHPF ASFISLVCPS SENDYSEQWA LACGEILRIL THYNRPIYKT EQQ-NGDTER NCLSKATTSG SP-TSE-PKA GSPTQHERKP
A. lyrata_GI 91 PIISCLIDGS LVYSKEAHPF ASFISLVCPS SENDYSEQWA LACGEILRIL THYNRPIYKT EQQ-NGETER NCFSKATTSG SP-TSE-PKA LSPTQHERKP
C. rubella_GI 89 PIISCLIDGS LVYSKEAHPF ASFISLVCPS SENDYSEQWA LACGEILRIL THYNRPIYKT EQQ-NGDTER NCLNKATTSG SP-PSE-SKA VSPTHNERKP
C. grandiflora_GI 89 PIISCLIDGS LVYSKEAHPF ASFISLVCPS SENDYSEQWA LACGEILRIL THYNRPIYKT EQQ-NGETER NCLNKATTSG SP-PSE-SKA VSPTHNERKP
B. rapa FPsc_GI 90 PIISCLIDGT LVYSKEAHPF ASFISLVSPN SENDYSEQWA LACGEILRIL THYNRPIYKT EQQ-NGETES ----KASTSG SPTLSE-AKA VSPGQHERKP
E. salsugineum_GI 88 PMISCLIDGS LVYSKEAHPF ASFISLVCPS SENDYSEQWA LACGEILRIL THYNRPIYKT EQQ-NGETEI NCLSKATTSG SA-LSE-PKA VSPVQHERKP
G. raimondii_GI 88 PIISGIIDGS LVYDKSSLPF ASFISLVCPS SENEYSEQWA LACGEILRIL THYNRPIYKM EPQ-NNETDR SHSSSQATTS ESVDGEPSFQ IPLMQQERKP
T. cacao_GI 88 PIISCIIDGT LVYDKSTPPF ASFISLVCPS SENEYSEQWA LACGEILRIL THYNRPIYKM EQQ-NSETDR SNSSGQATTS EPVDGEPSFH IPLMQQERKP
C. sinensis_GI 88 PIISCIIDGT LVYDKSSPPF ASFVSLVCPN SENEYSEQWA LACTEILRIL THYNRPIYKT EQQ-NSEFER SSSSSHATTS DSRDGE-LSN MPLVQQERKP
M. esculenta_GI 88 PIISCLIDG- LVYDRSSPPF ASFISLVCPS SENEYSEQWA LACGEILRIL THYNRPVYKV EQQ-NSETER SNGGNHATSS GSVDRE-SCP TPSVQQERKP
R. communis_GI 80 PIISCLIDGT LVYDRSTPPF ASFISLVCPS SENEYSEQWA LACGEILRVL THYNRPIYKK EQQ-KSETEK SGGGEDAVNG GLADGE-SSH TPPAQQERKP
P. trichocarpa_GI 89 PIISCIIDGT LVYDRSSPPF ASFISLVCPG SENEYSEQWA LACGEILRIL THYNRPIYKR EQQ-NNETDR SSSDSHATSS ESAEGK-STS MPLVQQERKP
S. purpurea_GI 89 PIISCIIDGT LVYDRSSPPF ASFISLVCPG NENEYSEQWA LACGEILRIL THYNRPIYKR EQK-NNETDR -TSDSHATTS ESAEGK-SSS MPLVQQERKP
V. vinifera_GI 88 PIISCIIDGT LVYDRCTPPF ASFISLVCPS SENEYSEQWA LACGEILRIL THYNRPIYKV EHQ-SSEADR SSSGRHATTS DSVDGK-SSQ GPLLQNERKP
S. lycopersicum_GI 88 PIISCIIDGT LDYDKSCPPF ASFISLVCPS SEKEYSEQWA LACGEILRIL THYNRPIYKV VQQ-GGEADR SSQGIDASTS KSADSG--PS MPSVHHERKT
M. guttatus_GI 88 PIISCIIDGL VEYKRSGPPF ASFISLVCPN SENEYSEQWA LACGEILRIL THYNRPIYKR ERQ-ENETDR SSSGTLASTS KSTDGE--PS LPSTQLERKM
A. coerulea_GI 88 PIISCIIDGT LVYDKNSPPF SSFISLVRPS SENEYSEQWA LACGEILRVL THYNRPIYKV ENQ-SRESER SSSGSHATTS NSKSKE-ICP PILQQQEKNP
S. bicolor_GI 90 PILSRIIDGT LCYDRHGPPF SSFISLFSHT SEQEYSEQWA LACGEILRVL THYNRPIFKV ERQ-HSEAEC SSTSDQATSS DSTDKK-SNN SPGNESDRKP
Z. mays_GI 90 PILSPIIDGT LCYDRHGPPF SSFISLFSHT SEQEYSEQWA LACGEILRVL THYNRPIFKV ERQ-HSEAEC STTSDQATSS DSTDKK-SNN SLGNESDRKP
S. italica_GI 90 PILSRIIDGT LCYDRHGAPF SSFISLFSHT SEKEYSEQWA LACGEILRVL THYNRPIFKV ERQ-HSEAEC SSTSDQATSS DSTDKK-SDD SPGTEPDRKP
P. virgatum_GI 90 PILSRIIDGT LCYDRHGPPF SSFISLFSHT SEKEYSEQWA LACGEILRVL THYNRPIFKA ERQ-RCEAEC SSTSDQATSS DSTDEK-SDG SPGNEPDRKP
O. sativa_GI 90 PILSRIIDGT LSYDRNGFPF MSFISLFSHT SEKEYSEQWA LACGEILRVL THYNRPIFKV DHQ-HSEAEC SSTSDQASSC ESMEKR-ANG SPRNEPDRKP
B. distachyon_GI 101 PILSRIIDGT LSYDRHGFPF NSFISLFTQT SEKEYSEQWA LACGEILRVL THYNRPIFKV -------AER NSTSDQATAS YSVQEK-ANG SPGNEPDRKP
A. trichopoda_GI 61 PILSCIIDGT LIYDKGNPPF SSFISLFSPS SEKDYSEQWA LACGEILRVL THYNRPIFKV EKQ-NCELER SSSGNYATTS SGREGK-ACH PLLMEPEKKP
P. abies_GI 88 PLLSCIIDGT LIYDKKMSPF SSFNSLFSPS SENDYSEQWA LACGEILRVL THYNRPVYKV DRTYKQETER CVSGNSASTS NSRENGISGH SIRQDQDKKP
S. moellendorffii_GI 88 PLLSCVIDGT LAYSKTTPPF GSFVSVFGVS SERDLTEQWA LACGEILRLL THYNRPIYKS ESS--ADGEK RSSSDSGDPA DRDGSG---- -SPDNGRRAP
210 220 230 240 250 260 270 280 290 300
M. truncatula_GI 191 IRPLSPWITD ILLVAPVGIR SDYFRWCSGV MGKYAAG-EL KPPSIATSRG SGKHPQLVPS TPRWAVANGA GVILSVCDDE VARNETAILT AAAVPALLLP
G. max_GI 191 IRPLSPWITD ILLASPVGIR SDYFRWCSGI MGKYAAG-EL KPPSTASSRG SGKHPQLVPS TPRWAVANGA GVILSVCDDE VARNETATLT AAAVPALLLP
C. sativus_GI 186 LRPLSPWITD ILLAAPLGIR SDYFRWCSGV MGKYAVR-EL KPPTTATSRG SGKHPQLMPS TPRWAVANGA GVILSVCDEE VARYETATLT AAAVPALLLP
M. domestica_GI 186 IRPLSPWITD ILLAAPLGIR SDYFRWCSGV MGKYAAG-EL KPPSTASSRG SGKHPQLMPS TPRWAVANGA GVILSVCDEE VARYETATLT AVAVPALLLP
F. vesca_GI 186 IRPLSPWITD ILLAAPLGIR SDYFRWCSGV MGKYAAG-EL KPPSTASSRG SGKHPQLMPS TPRWAVANGA GVILSVCDEE VSRYETATLT AVAVPALLLP
A. thaliana_GI 187 LRPLSPWISD ILLAAPLGIR SDYFRWCSGV MGKYAAG-EL KPPTIAS-RG SGKHPQLMPS TPRWAVANGA GVILSVCDDE VARYETATLT AVAVPALLLP
A. lyrata_GI 188 LRPLSPWISD ILLAAPLGIR SDYFRWCSGV MGKYAAG-EL KPPTIAS-RG SGKHPQLMPS TPRWAVANGA GVILSVCDDE VARYETATLT AVAVPALLLP
C. rubella_GI 186 LRPLSPWISD ILLAAPLGIR SDYFRWCSGV MGKYAAG-EL KPPTIAS-RG SGKHPQLMPS TPRWAVANGA GVILSVCDDE VARYETATLT AVAVPALLLP
C. grandiflora_GI 186 LRPLSPWISD ILLAAPLGIR SDYFRWCSGV MGKYAAG-EL KPPTIAS-RG SGKHPQLMPS TPRWAVANGA GVILSVCDDE VARYETATLT AVAVPALLLP
B. rapa FPsc_GI 184 LRPLSPWISD ILLAAPLGIR SDYFRWCSGV MGKYAAG-EL KPPTIAT-RG SGKHPQYMPS TPRWAVANGA GVILSVCDDE VARYETATLT AVAVPALLLP
E. salsugineum_GI 185 LRPLSPWISD ILLAAPLGIR SDYFRWCSGV MGKYAAG-EL KPPTIAS-RG SGKHPQLMPS TPRWAVANGA GVILSVCDDE VARYETATLT AVAVPALLLP
G. raimondii_GI 187 LRPLSPWITD ILLAAPLGIR SDYFRWCSGV MGKYAAG-DL KPPTTASSRG SGKHPQLMPS TPRWAVANGA GVILSVCDEE VARYETASLT AAAVPALLLP
T. cacao_GI 187 LRPLSPWITD ILLAAPLGIR SDYFRWCSGV MGKYAAG-DL KPPSTASSRG SGKHPQLMPS TPRWAVANGA GVILSVCDEE VARYETATLT AAAVPALLLP
C. sinensis_GI 186 LRPLSPWITD ILLAAPLGIR SDYFRWCSGV MGKYAAG-EL KPPTIASSRG SGKHPQLMLS TPRWAVANGA GVILSVCDDE LARYETATLT AAAVPALLLP
M. esculenta_GI 185 LRPLSPWITD ILLAAPLGIR SDYFRWCSGV MGKYAAG-EL KPPITASSRG SGKHPQLMPS TPRWAVANGA GVILSVCDEE VARYETATLT AAAVPALLLP
R. communis_GI 178 LRPLSPWITD ILLTAPLGIR SDYFRWCSGV MGKYAGG-EL KPPTTASSHG SGKHPQLMPS TPRWAVANGA GVILSVCDEE VARYETATLT AAAVPALLLP
P. trichocarpa_GI 187 FRPLSPWITD ILLAAPLGIR SDYFRWCSGV MGKYAAG-EL KPPTTTSSRG SGKHPQLIPS TPRWAVANGA GVILSVCDEE VARYETATLT AAAVPALLLP
S. purpurea_GI 186 FRPLSPWITD ILLAAPLGIR SDYFRWCSGV MGKYAAG-EL KPPTTTSSRG SGKHPQLIPS TPRWAVANGA GVILSVCDEE VTRYETATLT AAAVPALLLP
V. vinifera_GI 186 SRPLSPWITD ILLAAPLGIR SDYFRWCSGV MGKYAAG-EL KPPSTASTRG SGKHPQLIPS TPRWAVANGA GVILSVCDEE VARYETATLT AAAVPALLLP
S. lycopersicum_GI 185 LRPLSPWITD ILLTAPLGIR SDYFRWCGGV MGKYAAG-EL KPPSTASSRG SGKHPQLIPS TPRWAVANGA GVILSVCDEE VARYETATLT AAAVPALLLP
M. guttatus_GI 185 LRPLSPWITD ILLAAPLGIR SDYFRWCGGV MGKYAAG-EL KPPSTAFSRG SGKHPQLVPS TPRWAVANGA GVILSVCDDE VARYETATLT AASVPALLLP
A. coerulea_GI 186 LRPLSPWITD ILLAAPLGIR SDYFRWCGGV MGKYAAAGEL KPPTAACGRR SGKHPQLIPS TPRWAVANGA GVILSVCDEE VARYETATLT AVAVPALLLP
S. bicolor_GI 188 LRPLTPWITD ILLAAPLGIR SDYFRWCSGV MGKYAAGGEL KPPTTACSRG SGKHPQLMPS TPRWAVANGA GVILSVCDEE VARYETANLT AAAVPALLLP
Z. mays_GI 188 LRPLTPWITD ILLAAPLGIR SDYFRWCSGV MGKYAAGGEL KPPTTAYSRG SGKHPQLMPS TPRWAVANGA GVILSVCDEE VARYETANLT AAAVPALLLP
S. italica_GI 188 LRPLTPWITD ILLAAPLGIR SDYFRWCGGV MGKYAAGGEL KPPTTACSRG SGKHPQLMPS TPRWAVANGA GVILSVCDEE VARYETANLT AAAVPALLLP
P. virgatum_GI 188 LRPLTPWITD ILLAAPLGIR SDYFRWCGGV MGKYAAGGEL KPPTTACSRG SGKHPQLMPS TPRWAVANGA GVILSVCDEE VARYETANLT AAAVPALLLP
O. sativa_GI 188 LRPLSPWITD ILLAAPLGIR SDYFRWCGGV MGKYAAGGEL KPPTTAYSRG SGKHPQLMPS TPRWAVANGA GVILSVCDEE VARYETANLT AAAVPALLLP
B. distachyon_GI 193 LRPLSPWITD ILLTAPLGIR SDYFRWCGGV MGKYAAGGEL KPPTTAYSRG AGKHPQLMPS TPRWAVANGA GVILSVCDEE VARYETANLT AAAVPALLLP
A. trichopoda_GI 159 LRPLSPWITD ILLAAPLGIR SDYFRWCGGV MGKYAAGGEL KPPTTASGRG SGKHPQLMPS TPRWAVANGA GVILSVCDEE VARYETANLT AAAVPALLLP
P. abies_GI 188 LRLLTPWITD ILLAAPLGIR SDYFRWCGGV KGKYAAGGEL KPPTTAGGRG PGKHPQLMPS TPRWAVANGA GVILSVCDDE VTRYETANLT AAAVPALLLP

```
S. moellendorffii_GI   181  KRLLTPWITD SLLAAPLGTK SDYFRWCGGV LGKYACGGDL RPPTTGDGKG HGKHPQLLSS TPRWAVANGA AVISSVCDDE VLRYETADLT AAAVPALLLP
```

```
                                   310        320        330        340        350        360        370        380        390        400
                            ....|....| ....|....| ....|....| ....|....| ....|....| ....|....| ....|....| ....|....| ....|....| ....|....|
M. truncatula_GI       290  PPTTALDEHL VAGLPALEPY ARLFHRYYAI ATPSATQRLL LGLLEAPPSW APDALDAAVQ LVELLRAAEE YASG-IRLPR NWMHLHFLRA IGTAMSMRAG
G. max_GI              290  PPTTALDEHL VAGLPALEPY ARLFHRYYAI ATPSATQRLL LGLLEAPPSW APDALDAAVQ LVELLRAAED YASG-IRLPR NWMHLHFLRA IGTAMSMRAG
C. sativus_GI          285  PPTTALDEHL VAGLPALEPY ARLFHRYYAI ATPSATQRLL LGLLEAPPSW APDALDAAVQ LVELLRAAED YASG-IRLPR NWMHLHFLRA IGTAMSMRVG
M. domestica_GI        285  PPTTALDEHL VAGLPALEPY ARLFHRYYAI ATPSATQRLL LGLLEAPPSW APDALDAAVQ LVELLRAAED YXSG-IRLPR NWMHXHFLRA IGTAMSMXAG
F. vesca_GI            285  PPTTALDEHL VAGLPALEPY ARLFHRYYAI ATPSATQRLL LGLLEAPPSW APDALDAAVQ LVELLRAAED YASG-IRLPR NWMHLHFLRA IGTAMSMRAG
A. thaliana_GI         285  PPTTSLDEHL VAGLPALEPY ARLFHRYYAI ATPSATQRLL LGLLEAPPSW APDALDAAVQ LVELLRAAED YASG-VRLPR NWMHLHFLRA IGIAMSMRAG
A. lyrata_GI           286  PPTTSLDEHL VAGLPALEPY ARLFHRYYAI ATPSATQRLL LGLLEAPPSW APDALDAAVQ LVELLRAAED YASG-VRLPR NWMHLHFLRA IGIAMSMRAG
C. rubella_GI          284  PPTTSLDEHL VAGLPALEPY ARLFHRYYAI ATPSATQRLL LGLLEAPPSW APDALDAAVQ LVELLRAAED YASG-VRLPR NWMHLHFLRA IGIAMSMRAG
C. grandiflora_GI      284  PPTTSLDEHL VAGLPALEPY ARLFHRYYAI ATPSATQRLL LGLLEAPPSW APDALDAAVQ LVELLRAAED YASG-VRLPR NWMHLHFLRA IGIAMSMRAG
B. rapa FPsc_GI        282  PPTTSLDEHL VAGLPALEPY ARLFHRYYAI ATPSATQRLL LGLLEAPPSW APDALDAAVQ LVELLRAAED YASG-VRLPR NWMHLHFLRA IGIAMSMRAG
E. salsugineum_GI      283  PPTTSLDEHL VAGLPALEPY ARLFHRYYAI ATPSATQRLL LGLLEAPPSW APDALDAAVQ LVELLRAAED YASG-VRLPR NWMHLHFLRA IGIAMSMRAG
G. raimondii_GI        286  PPTTALDEHL VAGLPALEPY ARLFHRYYAI ATPSATQRLL LGLLEAPPSW APDALDAAVQ LVELLRAAED YATG-IRLPR NWMHLHFLRA IGTAMSMRAG
T. cacao_GI            286  PPTTALDEHL VAGLPALEPY ARLFHRYYAI ATPSATQRLL LGLLEAPPSW APDALDAAVQ LVELLRAAED YATG-IRLPR NWMHLHFLRA IGTAMSMRAG
C. sinensis_GI         285  PATTALDEHL VAGLPALEPY ARLFHRYYAF ATPSATQRLL LGLLEAPPSW APDALDAAVQ LVELLRAAEE YATG-IKLPR NWMHLHFLRA IGVAMSMRAG
M. esculenta_GI        284  PPTTALDEHL VAGLPALEPY ARLFHRYYAI ATPSATQRLL LGLLEAPPSW APDALDAAVQ LVELLRAAED YASG-IRLPR NWMHLHFLRA IGIAMSMRAG
R. communis_GI         277  PPTTALDEHL VAGLPALEPY ARLFHRYYAF ATPSATQRLL LGLLEAPPSW APDALDAAVQ LVELLRAAED YASG-IRLPR NWMHLHFLRA IGIAMSMRAG
P. trichocarpa_GI      286  PPTTALDEHL VAGLPALEPY ARLFHRYYAI ATPSATQRLL LGLLEAPPSW APDALDAAVQ LVELLRAAED YASG-IRLPR NWMHLHFLRA IGTAMSMRAG
S. purpurea_GI         285  PPTTALDEHL VAGLPALEPY ARLFHRYYAI ATPSATQRLL LGLLEAPPSW APDALDAAVQ LVELLRAAED YASG-IRLPR NWMHLHFLRA IGTAMSMRAG
V. vinifera_GI         285  PPTTALDEHL VAGLPALEPY ARLFHRYYAI ATPSATQRLL LGLLEAPPSW APDALDAAVQ LVELLRAAED YASG-MRLPR NWMHLHFLRA IGTAMSMRAG
S. lycopersicum_GI     284  PPTTPMDEHL VAGLPALEPY ARLFHRYYAI ATPSATQRLL LGLLEAPPSW APDALDAAVQ LVELLRAAED YATN-LRLPR NWMHLHFLRA IGIAMSMRAG
M. guttatus_GI         284  PPTTPMDEHL VAGLPALEPY ARLFHRYYAI ASPSATQRLL LGLLEAPPSW APDALDAAVQ LVELLRAAED YASG-MRLPR NWMHLHFLRA IGIAMSMRAG
A. coerulea_GI         286  PPTTAMDEHL VAGLPALEPY ARLFHRYYAI ATPSATQRLL LGLLEAPPSW APDALDAAVQ LVELLRAAED YTTG-MRLPR NWMHLHFLRA IGIAMSMRAG
S. bicolor_GI          288  PPTTPLDEHL VAGLPPLEPY ARLFHRYYAI ATPSATQRLL FGLLEAPPSW APDALDAAVQ LVELLRAAED YASG-MRLPK NWMHLHFLRA IGTAMSMRAG
Z. mays_GI             288  PPTTPLDEHL VAGLPPLEPY ARLFHRYYAI ATPSATQRLL FGLLEAPPSW APDALDAAVQ LVELLRAAED YASG-MRLPK NWMHLHFLRA IGTAMSMRAG
S. italica_GI          288  PPTPALDEHL VAGLPPLEPF ARLFHRYYAI ATPSATQRLL FGLLEAPPSW APDALDAAVQ LVELLRAAED YATG-MRLPK NWMHLHFLRA IGTAMSMRAG
P. virgatum_GI         288  PPTTPLDEHL VAGLPPLEPY ARLFHRYYAI ATPSATQRLL FGLLEAPPSW APDALDAAVQ LVELLRAAED YATG-MRLPK NWMHLHFLRA IGTAMSMRAG
O. sativa_GI           288  PPTTPLDEHL VAGLPPLEPY ARLFHRYYAI ATPSATQRLL FGLLEAPPSW APDALDAAVQ LVELLRAAED YDSG-MRLPK NWMHLHFLRA IGTAMSMRAG
B. distachyon_GI       293  PPTTPLDEHL VAGLPPLEPY ARLFHRYYAI ATPSATQRLL FGLLEAPPSW APDALDAAVQ LVELLRAAED YATG-MRLPK NWLHLHFLRA IGTAMSMRAG
A. trichopoda_GI       259  PP-TAADEHL VAGLPALEPY ARLFHRYYAI ATPSATQRLL LGLLEAPPSW APDALDAAVQ LVELLRAAED YATG-MRLPR NWMHLHFLRA IGIAMSMRAG
P. abies_GI            288  PPTTALDEHL VAGLPPLEPY ACLFHRYYAI ATPSATQRLL LGLLEAPPSW APDALDAAVQ LVELLRAAED YAST-MRLPK NWLHLHFLRA IGTALSMRVG
S. moellendorffii_GI   281  PPSTSLDEHL VAGLPPLEPF ARLFHRYYAI ATPCATQRLL LGLLEAPASW APDALDAAVQ LVELLRAAED YSSSSFRLPE NWFRLHFLRP MGAAMTMKQG
```

```
                                   410        420        430        440        450        460        470        480        490        500
                            ....|....| ....|....| ....|....| ....|....| ....|....| ....|....| ....|....| ....|....| ....|....| ....|....|
M. truncatula_GI       389  IAADAAAALL FRILSQPALL FPPLRQVDGV EVQHEPLGG- -YISSYSKQ- IEVPSAEASI DATAQGIASM LCAHGPEVEW RICTIWEAAY GLIPASSSAV
G. max_GI              389  IAADAAAALL FRILSQPALL FPPLRQVDGV EVQHEPLGG- -YISSYKKQ- IEVPAAEASI EATAQGIASM LCAHGPEVEW RICTIWEAAY GLIPTSSSAV
C. sativus_GI          384  IAADAAAALL FRILSQPALL FPPLRQVEGA EIQHEPLGD- -YISSYQRQ- IEVPAAEATI EATAQGIASM LCAHGLEVEW RICTIWEAAY GLIPLSSSAV
M. domestica_GI        384  IAADAAAALL FRILSQPALL FPPLRQVDGV EVQHEPLGG- -YISSYKKQ- IELPAAEATI EATAQGIASM LCAHGPXVEW RICTIWEAAY GLIPLSSSAV
F. vesca_GI            384  IAADAAAALL FRILSQPALL FPPLRQVEGV EVQHEPMGS- -RVSSYRKQ- IEVPAAEATI EATAQGIASM LCAHGPEVEW RICTIWEAAY GLIPLSSSAV
A. thaliana_GI         384  VAADAAAALL FRILSQPALL FPPLSQVEGV EIQHAPIGG- -YSSNYRKQ- IEVPAAEATI EATAQGIASM LCAHGPEVEW RICTIWEAAY GLIPLNSSAV
A. lyrata_GI           385  VAADAAAALL FRILSQPALL FPPLSQVEGV EIQHAPIGG- -YSSNYRKQ- IEVPAAEATI EATAQGIASM LCAHGPEVEW RICTIWEAAY GLIPLNSSAV
C. rubella_GI          383  VAADAAAALL FRILSQPALL FPPLSQVEGV EIQHAPIGG- -YSSNYRKQ- IEVPAAEATI EATAQGIASM LCAHGPEVEW RICTIWEAAY GLIPLNSSAV
C. grandiflora_GI      383  VAADAAAALL FRILSQPALL FPPLSQVEGV EIQHAPIGG- -YSSNYRKQ- IEVPAAEATI EATAQGIASM LCAHGPEVEW RICTIWEAAY GLIPLNSSAV
B. rapa FPsc_GI        381  VAADAAAALL FRILSQPALL FPPLSQAEGV EIKHAPIGG- -YGSNYRKQ- IEVPAAEATI EATAQGIASM LCAHGPEVEW RICTIWEAAY GLIPLNSSAV
E. salsugineum_GI      382  VAADAAAALL FRILSQPALL FPPLCQVEGV EIQHAPIGG- -YSSNYRKQ- IEVPAAEATI EATAQGIASM LCAHGPEVEW RICTIWEAAY GLIPLNSSAV
G. raimondii_GI        385  IAADAAAALL FRILSQPALL FPPIRQVEGV EVQHEPSGG- -YISCYRKQ- IEVPAAEATI EATAQGIASM LCAHGPEVEW RICTIWEAAY GLIPLSSSAV
T. cacao_GI            385  IAADAAAALL FRILSQPALL FPPLRQVEGV EVQHEPSGG- -YISCYRKQ- IEVPAAEATI EATAQGIASM LCAHGPEVEW RICTIWEAAY GLIPLSSSAV
C. sinensis_GI         384  IAADAAAALL FRILSQPALL FPPLRQVDGV EVQHEPLGG- -YISCYRKQ- IEVPAAEATI EATAQGIASV LCAHGPEVEW RICTIWEAAY GLIPLSSSAV
M. esculenta_GI        383  IAADAAAALL FRILSQPALL FPPLRQVEGV EVQHEPLGG- -YFSSYRKQ- IEVPAAEATI EATAQGIASM LCAHGPEVEW RICTIWEAAY GLIPLCSSAV
R. communis_GI         376  IAADAAAALL FRILSQPALL FPPLRQVEGM EVHHEPLGA- -YSSSYRKQ- IEVPAAEATI EATAQGIASM LCAHGPEVEW RICTIWEAAY GLLPLCSSAV
P. trichocarpa_GI      385  IAADAAAALL FRILSQPALL FPPLRQVEGV EVQHEPLGG- -YISCYRKQ- IEVPAAEATI EATAQGIASM LCAHGPEVEW RICTIWEAAY GLIPLSSSAV
S. purpurea_GI         384  IAADAAAALL FRILSQPALL FPPIRQVEGV EAQHEPLGG- -YISCYRKQ- IEVPAAQATI EATAQGIASM LCAHGPEVEW RICTIWEAAY GLIPLSSSAV
V. vinifera_GI         384  IAADAAAALL FRVLSQPALL FPPLRQVEGF EVQHEPLDG- -YISSYKKQ- IEVPATEATI EATAQGIASM LCAHGPEVEW RICTIWEAAY GLIPLSSSAV
S. lycopersicum_GI     383  IAADAAAALL FRVLSQPALL FPPLRQVEGI EVQHEPLGG- -YISCNKKQ- RQVPLAEATV EATAQGIASM LCAHGPEVEW RICTIWEAAY GLIPLTSSAV
M. guttatus_GI         383  IAADAAAALL FRILSQPALL FPPLRQVEGI EAQHEPLGG- -CVSSERKQQ RELPAAEATV EATAQGIASM LCAHGPEVEW RICTIWEAAY GLIPLSSSSV
A. coerulea_GI         385  VAADAAAALL FRILSQPALL FPPLCQAEGV EVQHEPLSG- -YISSYSKL- KEEPAAEATI EATAQGIASM FCAHGPEVEW RICIIWEAAY GLIPLNSSAV
S. bicolor_GI          387  IAADTAAALL FRILSQPTLL FPPLRHAEGV EVHHEPLGG- -YVSSYKKQ- LEVPASEATI DATAQGIASL LCAHGPDVEW RICTIWEAAY GLLPLSSSAV
Z. mays_GI             387  IAADTAAALL FRILSQPTLL FPPLRHAEGV DVHHEPLGG- -YVSSYKKQ- LEVPASEATI DATAQGIASL LCAHGPDVEW RICTIWEAAY GLLPLSSSAV
S. italica_GI          387  IAADTAAALL FRILSQPTLL FPPLGHAEGV EVHHEPLGG- -YVSSYKRQ- LEVPASEATI DATAQGIASL LCAHGPDVEW RICTIWEAAY GLLPLSSSAV
P. virgatum_GI         387  IAADTAAALL FRILSQPTLL FPPLRHAEGV EVHHEPLGG- -YVSSYKRQ- LEVPASEATI DATAQGIASL LCAHGPDVEW RICTIWEAAY GLLPLSSSAV
O. sativa_GI           387  IAADTSAALL FRILSQPTLL FPPLRHAEGV ELHHEPLGG- -YVSSYKRQ- LEVPASEATI DATAQGIASM LCAHGPDVEW RICTIWEAAY GLLPLSSSAV
B. distachyon_GI       392  MAADTAAALL FRILSQPTLL FPPLRHAEGL EVHHEPLGG- -YVSSYKRQ- LEVPASETTI DATAQGIASL LCAHGPDVEW RICTIWEAAY GLLPLNSSAV
A. trichopoda_GI       357  IAADAAAALL FRILSQPTLL FPPPRHTEGI EVQNEPQGG- -SSSAYRKQ- VEVTAAEATI EATAQGVASL LCAHGPEVEW RICTIWEAAY GLLPLSSSTV
P. abies_GI            387  IAADTAAALL FRTLSQPALL FPPPRLAQGV DVQCDVYGA- -FGPSSSGEE IEMETYQASI EATAQGVASL MCLHGPEVEW RICTLWEAAY GLLPLSSSTV
S. moellendorffii_GI   381  IASDAAAALL FRLFSQPALL FPPRGHAQGA QVVQPLYGPP IRIDVLFHAQ MEALATQVNE EATAKGVASL MRDHGRDVEW RICVLWEAAY GLIPLDKSVV
```

```
                                   510        520        530        540        550        560        570        580        590        600
                            ....|....| ....|....| ....|....| ....|....| ....|....| ....|....| ....|....| ....|....| ....|....| ....|....|
M. truncatula_GI       486  DLPEIIVAAP LQPPILSWNL YIPLLKVLEY LPRGSPSEAC LMKIFAATVE AILQRTFPPE SSREQNRKAN YLF-GLG--S ASKNLAVAEL RTMVHSLFLE
G. max_GI              486  DLPEIIVATP LQPPVLSWNL YIPLLKVLEY LPRGSPSEAC LMKIFAATVE AILQRTFPPE STREQNRKSK YLA-GIGFGS ASKNLAVAEL RTMVHSLFLE
C. sativus_GI          481  DLPEIVVATP LQPPILSWNL YIPLLKVLEY LPHGSPSEAC LMKIFVATVE AILQRAFPSE SSTERTRKLK YLS-GIG--Y VSKNISVSEL RMMVHSLFLE
M. domestica_GI        481  DLPEIIVATP LQPPILSWNL YIPLLKVLEY LPRGSPSEAC LMKIFVATVE AILQRTFPSE SSREQNRKTR YLF-GLG--S ASKNLAVAEL RTMVHSLFLE
F. vesca_GI            481  DLPEIIVATP LQPPILSWNL YIPLLKVLEY LPRGSPSEAC LMKIFVATVE AILQRTFPPE SSREQNRKTR YLF-GIG--S ASKNLAVAEL RTMVHSLFLE
A. thaliana_GI         481  DLPEIIVATP LQPPILSWNL YIPLLKVLEY LPRGSPSEAC LMKIFVATVE TILSRTFPPE SSRELTRKAR SSF-TTR--S ATKNLAMSEL RAMVHALFLE
A. lyrata_GI           482  DLPEIIVATP LQPPILSWNL YIPLLKVLEY LPRGSPSEAC LMKIFVATVE TILSRTFPPE SSREHTRKAR SSF-TTR--S ATKNLVMAEL RAMVHALFLE
C. rubella_GI          480  DLPEIIVATP LQPPILSWNL YIPLLKVLEY LPRGSPSEAC LMKIFVATVE TILSRTFPPE SSRDHTRKAR SSF-TTR--S ATKNLAMAEL RAMVHALFLE
C. grandiflora_GI      480  DLPEIIVATP LQPPILSWNL YIPLLKVLEY LPRGSPSEAC LMKIFVATVE TILSRTFPPE SSRDHTRKAR SSF-TTR--S ATKNLAMAEL RAMVHALFLE
B. rapa FPsc_GI        478  DLPEIIVATP LQPPILSWNL YIPLLKVLEY LPRGSPSEAC LMKIFVATVE TILSRTFPPE TS---TRKAR ASL-ATRS-S ATKNLAMAEL RAMVHALFLE
E. salsugineum_GI      479  DLPEIIVATP LQPPILSWNL YIPLLKVLEY LPRGSPSEAC LMKIFVATVE TILSRTFPPE SSREHVRKAR SSF-TTR--S ATKNLAMAEL RAMVHALFLE
G. raimondii_GI        482  DLPEIIVSTP LQPPILSWNL YIPLLKVLEY LPRGSPSEAC LMKIFVATVE AILQRTFPPE SPREQTRKTR YS---IG--S ASKNLAVAEL RTMVHSLFLE
T. cacao_GI            482  DLPEIIVATP LQPAILSWNL YIPLLKVLEY LPRGSPSEAC LMKIFVATVE AILQRTFPPE SSRVQTRKTR YS---IG--S ASKNLAVAEL RTMVHSLFLE
C. sinensis_GI         481  DLPEIIVATP LQPPILSWNL YIPLLKVLEY LPRGSPSEAC LMKIFVATVE AIIKRTFPPE SSPENTRRAR HLS-GIG--S ASKNLAVAEL RTMVHSLFLE
M. esculenta_GI        480  DLPEIVVATP LQPPILSWNL YIPLLKVLEY LPRGSPSEAC LMKIFVATVE AILTRTFPPQ SSREQTRKAR YFS-GLG--S ASKNLAVAEL RTMVHSLFLE
R. communis_GI         473  DLPEIIVAAP LQPPILSWNL YIPLLKVLEY LPRGSPSEAC LIKIFVATVE AILQRTFPPE SSREQTRKAK YLF-GLG--S ASKNLAVAEL RTMVHSLFLK
P. trichocarpa_GI      482  DLPEIIVATP LQPPLLSWNL YIPLLKVLEY LPRGSPSEAC LMKIFVATVE AILQRTFPPE SSREQTRRTR YFS-SLG--P ASKNLAVAEL RTMVHSLFLE
S. purpurea_GI         481  DLPEIIVATP LQPPILSWNL YIPLLKVLEY LPRGSPSEAC LMKIFVATVE AILQRTFPPE SSREQTRKTR YLS-SLG--P ASKNLAVAEL RTMIHSLFLE
V. vinifera_GI         481  DLPEIIVATP LQPPILSWNL YIPLLKVLEY LPRGSPSEAC LMKIFVATVE SILQRTFPAE SSRENIRKTR YLF-GIG--S ASKNLAVAEL RTMVHALFLE
S. lycopersicum_GI     480  DLPEIIVATP LLPPILSWNL YMPLLKVLEY LPRGSPSETC LMKIFVATVE AILQRTFPSE SSREEIRRNR YN--MFG--P ASKNLAVAEL RTMVHSLFLE
M. guttatus_GI         481  DLPEIIVATP LQPPILSWNL YIPLLKVLEY LPRGSPSETC LMKIFVATVE AILQRTFPPE STREKNRKTR Y---VFG--S AFKNLAVAEL RTMVHSLFLE
A. coerulea_GI         482  DLPEIIVATP MQPPVLSWNL YFPLLKVLDY LPRGSPSEAC LMRIFVATVE AILRRTFPSQ SSREQIRKSR YLY-GLG--S ASKNLAVAEL RTMIHSLFVE
S. bicolor_GI          484  DLPEIVVAAP LQPPTLSWSL YLPLLKVFEY LPRGSPSEAC LMRIFVATVE AILRRAFPSE TS-EQSRKPR SQ-------- -SKNLAVAEL HTMIHSLFVE
Z. mays_GI             484  DLPEIVVAAP LQPPTLSWNL YLPLLKVFEY LPRGSPSEAC LMRIFVATVE AILRRAFPSE TP-EQSRKPR SQ-------- -SKNLAVAEL HTMIHSLFVE
S. italica_GI          484  DLPEIVVAAP LQPPTLSWSL YLPLLKVFEY LPRGSPSEAC LMRIFVATVE AILRRTFPSE TL-EQSRKPR SQ-------- -SKNLAVAEL RTMIHSLFVE
P. virgatum_GI         484  DLPEIVVAAP LQPPTLSWSL YLPLLKVFEY LPRGSPSEAC LMRIFVATVE AILRRTFPSE TS-EQSRKPR SQ-------- -SKNLAVAEL RTMIHSLFVE
O. sativa_GI           484  DLPEIVVAAP LQPPTLSWSL YLPLLKVFEY LPRGSPSEAC LMRIFVATVE AILRRTFPSE TS-EQSRKPR SQ-------- -SKNLAVAEL RTMIHSLFVE
B. distachyon_GI       489  DLPEIVVAAP LQPPTLSWSL YLPLLKVFEY LPRGSPSEAC LMRIFVATVE AILRRTFPSE TS-EPSRKPR SQ-------- -SKNLAVAEL RTMIHSLFVE
A. trichopoda_GI       454  DLPEIVVATP LQPPVLSWNL YLPLLKVLEY LPRGSPSEAC LMRIFVATVE AVLRRTFPPE SSKECTRRPR HPHGGIG--S ASKNLAVAEL RTMVHSLFIE
```

```
P. abies_GI              485  DLPEIVVATP LQPPVLSWNL FQPLLRVLEY LPRGSPSEAS LMRIFTATVQ AILQRTFPAE QSTEQTRTTR NAHAGVG--P TSKNLAVAEL RTMVHSLFTE
S. moellendorffii_GI     481  DLPEMVIATP LQPPLLSWTL FRPFLRVLEH VPKGCQSQTC LRRIFSATVD AILRRTFPLD DWKEQKNGNF RSASGSG--- -VDPAGMAEL RALVHCLFTE

                                         610        620        630        640        650        660        670        680        690        700
                              ....|....| ....|....| ....|....| ....|....| ....|....| ....|....| ....|....| ....|....| ....|....| ....|....|
M. truncatula_GI         583  SCASVELSSR LLFVVLTVCV SHEAQF--SG SKKPRGEDNY SVEEIIEDLQ AISEIRKER- KNRKVKKQGP VAAFDSYVMA AVCALACELQ LFPLMSRGNN
G. max_GI                585  SCASVELASR LLFVVLTVCV SHEAQF--SG SKRPRGEDNY SSEDIIEDLQ TS-ENQKES- KNRKLKKQGP VAAFDSYVLA AVCALACELQ LFPLISRGNN
C. sativus_GI            578  SCASEELASR LLFIVLSVCV SHEAQS--NG RKKRRSGSSN FQDEKIEPSQ DISRESRET- KGWGNK-QGP VSAFDSYVLA AVCALACELQ LFPLMSRGRK
M. domestica_GI          578  SCXSVELASR LLFVVLTVCV SHEAQS--NG SKKARVDESF PPDESIEESE KMSDK-QRD- RTKKTKKQGP VAAFDSYVLA AVCALACELQ LFPLISKGTN
F. vesca_GI              578  SCASVELASR LLFVVLTVCV SHEAQS--NG SKKARVEESY PLEECVEESR EMSGK-QGD- R-KKTKKQGP VAAFDSYVLA AVCALACELQ LFPLVSRGSN
A. thaliana_GI           578  SCAGVELASR LLFVVLTVCV SHEAQS--SG SKRPRSEYAS TTENIEANQP VSNNQTANR- KSRNVKGQGP VAAFDSYVLA AVCALACEVQ LYPMISGGGN
A. lyrata_GI             579  SCAGVELASR LLFVVLTVCV SHEAQS--SG SKRPRSEYAI TTENVEANQP VSDNQTANR- KSRNVKGQGP VAAFDSYVLA AVCALACEVQ LYPMISGGGS
C. rubella_GI            577  SCAGVELASR LLFVVLTVCV SHEAQS--SG SKRPRSEYAS TAENIEAN-P VSDNQTSNR- KSRNVKGQGP VAAFDSYVLA AVCALACEVQ LYPMISGGGK
C. grandiflora_GI        577  SCAGVELASR LLFVVLTVCV SHEAQS--SG SKRPRSEYAS TAENIEAN-P VSDNQTSNR- KSRNVKGQGP VAAFDSYVLA AVCALACEVQ LYPMISGGGK
B. rapa FPsc_GI          573  SCAGVEIASR LLFVVLTVCV SHEAQS--SG SKRRRSEEVA TAE------E NQDNQTSNR- KSRNVKGQGP VAAFDSYVLA AVCALACEVQ LYPMISGGGN
E. salsugineum_GI        576  SCAGVELASR LLFVVLTVCV SHEAQS--CG SKRPRSEYAS TAE---ENQS VSDNQTTNR- KSRNVKGQGP VAAFDSYVLA AVCALACEVQ LYPMISGGGN
G. raimondii_GI          577  SCASIELASR LLFVVLTVCV SHEAQF--SG SKRPRGEESF PPDEGVEESQ AQSEKLKDI- KPRKAKKQGP VAAFDSYVLA AVCALACELQ LFPLVTRGNT
T. cacao_GI              577  SCASVELASR LLFVVLTVCV SHEAQF--SG SKRPRCEESY PPDEGIEESQ SPSERPRDI- KPRKTKKQGP VAAFDSYVLA AVCALACELQ LFPLVTRGSN
C. sinensis_GI           578  SCASVELASR LLFIVLTVCV SHEAQS--NG SKKPRGEENY FPDESTEDLQ ------KDL- RTRKVKRQGP VAAFDSYVLA AVCALACELQ LVPLVSRCGN
M. esculenta_GI          577  SCASVELASR LLFIVLTVCV SHEAQS--KG SKRPRDEESL PTDDSNEPSQ LTSEVQKNM- KYRKLKKQGP VAAFDSYVLA AVCALACELQ LFPFISRGSN
R. communis_GI           570  SCASVELASR LLFVVLTVCV SHEAQS--NG TKRPRGEENF QPDDGNEDWQ LTSEAHSKM- KPRKIKKQGP VAAFDSYVLA AVCALACELQ LFPFVSSGNN
P. trichocarpa_GI        579  SCASVELASR LLFVVLTVCV SHEAHS--RG SKRPRGEEND LPEDGTEDSQ STSEMRRNM- KSRNMKKQGP VAAFDSYVLA AVCALACELQ IFPFVSRGSN
S. purpurea_GI           578  SCASVELASR LLFVVLTVCV SHEAHT--RG SKRPRGEEND LPEDGTEDSQ STSETRRNM- KSGRMKKQGP IAAFDSYVLA AVCALACELQ IFPFVSRGSN
V. vinifera_GI           578  SCASVELASR LLFVVLTVCV SHEAAQQ-NG SKRPRGEDSH LSEEITEDLS DASGNQRDT- KTRKMKKQGP VAAFDSYVLA AVCALACELQ LFPLIARGTN
S. lycopersicum_GI       576  SCASVELASR LLFVVLTVCV THEAKT--NG SRRPVCKDPH HVSAMGSESL EVGGKQKEK- IPKKLKKQGP VSAFDSYVLA AVCALSCELQ LFPLLSRGSN
M. guttatus_GI           576  SCASVELSSR LLFVVLTVCV SHEAQP--NG SKRPKGED-- SCAVEGEDLQ RANGKHRDQ- GSKQCKKQGP IAAFDSFVIA AVCALSCELQ IFPLIAKQCS
A. coerulea_GI           579  TCASVDLASR LLFVILTVCV SHEARN--CL RKRPRSDDSY TPTDFAEDWH EVNEKQIDA- RTRKLKKQGP VAAFDSYVLA AVCALSCELQ LFPLLSRNGR
S. bicolor_GI            574  SCASMDLASR LLFVVLTVCV SHQALP--GG SKRPTGSDNH SHEEATEHSR LTNG------ RSRCKKRQGP VATFDSYVLA AVVALSCELD LFPCISKNGS
Z. mays_GI               574  SCASMDLASR LLFVVLTVCV SHQALP--GG SKRPTGSDNH SLEEATEHSR LTNG------ RSRCKKRQGP VATFDSYVLA AVCALSCELQ LFPCISKNGS
S. italica_GI            574  SCASMDLASR LLFVVLTVCV SHQALP--GG SKRPTGSDNH SSEEVTEDSR LTNG------ RNRCKKRQGP VATFDSYVLA AVCALSCELQ LFPFISKNGS
P. virgatum_GI           574  SCASMDLASR LLFVVLTVCV SHQALP--GG SKRPTGSDNH SSEEVAENSR LTNG------ RNRCKKRQGP VATFDSYVLA AVCALSCELQ LFPLISKNGS
O. sativa_GI             574  SCASMDLASR LLFVVLTVCV SHQALP--GG SKRPTGSDNH SSEEVTNDSR LTNG------ RNRCKKRQGP VATFDSYVLA AVCALSCELQ LFPFISKNGN
B. distachyon_GI         579  SCASMNLASR LLFVVLTVCV SHQALP--GG SKRPTGSDNH SSEEATEGSR LTNG------ RNRVKKRQGP VGTFDSYVLA AVCALSCELQ LFPILCKNGT
A. trichopoda_GI         552  SCASMDLASR LLFIVITVCV SHEALP--EG SKKPTGGEIG PSDEGPEEKQ IALNAKRTIG RNKTIKKQGP VAAFDSYVLA AVCALACELQ LFPLISQTIK
P. abies_GI              583  SGVPIDLASR LLFMVITVCL SHDAVQ--KG SRKTTNDRGA LSNSSMRDQQ AINGSQKVN- GHRAKKERGA VATFGSYILA AVCAQACEVQ LFSFTSPMVN
S. moellendorffii_GI     577  AFLGPALASQ LLSDALTVCL SHDTLRQGNG SDSSKKRSTH SSN------- ---------- -----KDRGA VASFDSYLIA AVCALACEVQ LCTFSAADG-

                                         710        720        730        740        750        760        770        780        790        800
                              ....|....| ....|....| ....|....| ....|....| ....|....| ....|....| ....|....| ....|....| ....|....| ....|....|
M. truncatula_GI         680  HSVSNNVQDI AKPVTLH--- -------GSS QDLQNGLDSA VRHTHRILAI LEALFSLKPS SVG-TPWSYS SNEIVAAAMV AAHVSELFRR SKACMHALSV
G. max_GI                681  HLASNKVQDI AKPVRLN--- -------GSS HELRNGLDSA VRHTHRILAI LEALFSLKPS SVG-TPWSYS SNEIVAAAMV AAHVSELFRR SKACMHALSV
C. sativus_GI            674  RLSFKSSQDI AKLIKIN--- -------GSS LELQSSIDSA IRRTHRILSI LEALFSLKPS SLG-TSWSYS SNEIVAAAMV AAHVSELFRR SRACMHALSV
M. domestica_GI          674  HAHSKNGKNV AKPAK----- -----VNVCT NEFQSSVDSA VCHTRRILAI LEALFSLKPS TIG-TSWSYS SNEIVAAAMV AAHVSELFRW XKACMHALSV
F. vesca_GI              673  QSHSKDAKNI AKPAKPIGSA NSYKQINGSS NEFQSSVDSA ICHTRRILVI LEALFLLKPS SVG-TSWSYS SNEIVAAAMV AAHVSELFRW SKACMHALCV
A. thaliana_GI           675  FSNSAVAGTI TKPVKIN--- -------GSS KEYGAGIDSA ISHTRRILAI LEALFSLKPS SVG-TPWSYS SSEIVAAAMV AAHISELFRR SKALTHALSG
A. lyrata_GI             676  FSNSAVARTI TKPVKIN--- -------GSS NEYGAGVDSA INHTRRILAI LEALFSLKPS SVG-TPWSYS SSEIVAAAMV AAHISELFRR SKALTHALSG
C. rubella_GI            673  FSNSAMAGTI TKPVKLN--- -------GST NEYGAGIDSA INHTRRILAI LEALFSLKPS SVG-TPWSYS SSEIVAAAMV AAHISELFRR SKALTHALSG
C. grandiflora_GI        673  FSNSAMAGTI TKPVKLN--- -------GST NEYGAGIDSA INHTRRILAI LEALFSLKPS SVG-TPWSYS SSEIVAAAMV AAHISELFRR SKALTHALSG
B. rapa FPsc_GI          664  FSNSAVAATI TKSVKIN--- -------GSS NEYGAGIDSA IKHTRRILAI LEALFSLKPS SVG-TPWSYS SSEIVAAAMV AAHISELFRR SKALTHALSG
E. salsugineum_GI        670  FSNSAVAGTI TKPVKIN--- -------GSS NQYGAGIDSA VNHTRRILAI LEALFSLKPS SVG-TPWSYS SSEIVAAAMV AAHISELFRR SKALTHALSG
G. raimondii_GI          674  HSTAKDVQAM ANPAKVN--- -------GSS IEYGHGIDSA IHHTHRILAI LEALFSLKPS SVG-TSWGYS SNEIVAAAMV AAHISELFRR SKACMYALSV
T. cacao_GI              674  HSTAKDVQAI AKPAKLN--- -------GSS IEYGHSIDSA IHHTHRILAI LEALFSLKPS SVG-TSWSYS SNEIVAAAMV AAHVSELFRR SKACMHALSV
C. sinensis_GI           669  HSKSKDAQIL AKPAK----- -----INGNS NECKSSIESA IHHTHRILTI LEALFSLKPS SIG-TSWGYS SNEIVAAAMV AAHVSELFRR SKACMHALSV
M. esculenta_GI          674  HSSSKNARTL AKPVKLNG-- -----CNGSS SEFQSSFDSA VHHTHRILAI LEALFSLKPS SVG-TSWSYS SNEIVAAAMV AAHVSELFRR SKACMHALSV
R. communis_GI           667  HSSSNDLDTL AKSMKMN--- -------GSI REFQNSIDSA VHHTHRILAI LEALFSLKPS TVG-TSWSYS SNEIVAAAMV AAHVSELFRR SKACMHALSV
P. trichocarpa_GI        676  HSTSKHSETV AKPAKLN--- -------GAV SEFQTSLNSA IHHTHRILTI LEALFSLKPS TIG-TSWSYS SNEIVAAAMV AAHVSELFRR SKACMHALSV
S. purpurea_GI           675  HSTSKHAETV AKPAKLN--- -------GVV SEFQTSLNSA IHHTHRILTI LEALFSLKPS SIG-TSWSYS SNEIIAAAMV AAHVSELFRR SKACMHALSV
V. vinifera_GI           676  HSASKDVQIR AKPAKLN--- -------GSS SEFRNSIDSA IRHTHRILAI LEALFSLKPS SVG-TSWSYS SNEIVAAAMV AAHVSELFRR SKACMHALSV
S. lycopersicum_GI       673  YSDP--KSIL VAAKHAN--- -------DSS MELKNGIHSA VCHTRRILTI LEALFSLKPS SIG-TSWSYS SNEIVAAAMV AAHISDLFRH SKACMHALSV
M. guttatus_GI           671  QLEAN-ISGV LKPVKGN--- -------DPP SEFQNSIDSA VYHTRRILTI LEALFSLKPS SIG-TSWSYS SNEIVAAAMV AAHVSDLFKR SKACMRALLI
A. coerulea_GI           676  HSVSKGASDI KLGQLN---- -------VPS NEFENSIFFA ISHTRRILGI LEALFSLKPS SIG-TSWSHS SNEIIAAAMV AAHISELFRQ SKPCMNALSI
S. bicolor_GI            666  HSNLKDSLKI IIPGKNN--- -------GIN NEPRSSISSA ILHTRRILGI LEALFSLKPS SVG-TSWSYS SNEIVAAAMV AAHVSELFRR SRPCLTSLSA
Z. mays_GI               666  HSNLKDSMKI IIPGKNN--- -------GIN NELHSSISSA ITHTRRILGI LEALFSLKPS SVG-TSWSYS SNEIVAAAMV AAHVSELFRR SRPCLNSLSA
S. italica_GI            666  HSNLKDSMKI IVPGKTN--- -------GIN KELHNSISSA ILHTRRILGI LEALFSLKPS SVG-TSWSYS SNEIVAAAMV AAHVSELFRR SRPCLNALSS
P. virgatum_GI           666  HSSIKDSMKI IVPGKTN--- -------GIN TELHNSISSA ILHTRRILGI LEALFSLKPS SVG-TSWGYS SNEIVAAAMV AAHVSELFRR SRPCLNALSA
O. sativa_GI             666  HSNLKDSIKI IVPGKTT--- -------GIS NELHNSISSA ILHTRRILGI LEALFSLKPS SVG-TSWSYS SNEIVAAAMV AAHVSELFRR SRPCLNALSA
B. distachyon_GI         671  HSNIKDSIKI IMPGKTN--- -------GIS NELQNSISSA VIHTRRILGI LEALFSLKPS SVG-TSWSYS SNEIVAAAMV AAHVSELFRR SRPCLNALSS
A. trichopoda_GI         650  GSDSKNSLTS APAANIN--- -------GGP NQLQNGICSA ISHTHRLLGI LEALFSLKPS SVG-TSWHYG SNEIVAAAMV AAHISELFGR SKACTHALSV
P. abies_GI              680  CVQPPNPVRA AIRIS----- -------GHV EGPPNGFYSA VNHTRRLLGI LEALLSLKPS AAGCTPGNYS SSEIIAAAMV AAHVSDLLGR SKACMHALSV
S. moellendorffii_GI     653  ---------- ---------- ---------- -TAFNGVTNS AYQARRLMSV LEGLLVVEPF SPGVGPNTNS PNDLVEAAIV AAHISRLLGR SRACTHALTA

                                         810        820        830        840        850        860        870        880        890        900
                              ....|....| ....|....| ....|....| ....|....| ....|....| ....|....| ....|....| ....|....| ....|....| ....|....|
M. truncatula_GI         769  LIRCKWNKEI HSRASSLYNL IDIHSKVVAS IVNKAEPLEA TLIHAPIYKD ALVCHDGKRK NRSENGSCSD PGQTSIVPSA DSTPSKHIHK SGRTP-CSNE
G. max_GI                770  LIRCKWDNEI HSRASSLYNL IDIHSKVVAS IVNKAEPLEA TLIHAPIRKD SLVCVGVKRQ NQCESSSCFD AGRTSVVPSE DSFPSKLDHN SNKTP-CPKG
C. sativus_GI            763  LMRCKWDEEI YTRALSLYNL IDIHSKAVAS IVNRAEPLGV HIVSAPISEY SRVSSACRKL TQHEDHVYFE NGQQSIPKCE ESCHVRAKLS FERA--SDST
M. domestica_GI          763  LMRCKWDSEI SSRAASLYNL IDFHSKAVAS IVNKAEPLEA HLMQVPIWRD SFVCFECRKL SQGGNSRCSN VGQPSALQCE DSSHSESKRK SDIA--SCSN
F. vesca_GI              772  LMRCKWDNEI SSRASSLYNL IDIHSKAVAS IVNKAEPLEA HLMQVPIWRD SLVCSECRKL SRCEKSKCIN VGQSSVSQYE GSAYSETRVK SVTP--SHSN
A. thaliana_GI           764  LMRCKWDKEI HKRASSLYNL IDVHSKVVAS IVDKAEPLEA YLKNTPVQKD SVTCLNWKQE NTCASTTCFD T-AVTSASRT EMNPRG-NHK YARH--SDEG
A. lyrata_GI             765  LMRCKWDKEI HKRASSLYNL IDVHSKVVAS IVDKAEPLEA YLKNTPVQKD SLTCLNWKQQ NTCASTTRFD T-AVTSASRT EMNPRG-NHK YARH--SGEG
C. rubella_GI            762  LMRCKWDKEI HKRASSLYNL IDVHSKVVAS IVDKAEPLEA YLKNTPVQKD SLTCLNWKQQ NTSASTSCFD T-AVTSASRT EMNPRG-NHK YARH--SDEG
C. grandiflora_GI        762  LMRCKWDKEI HKRASSLYNL IDVHSKVVAS IVDKAEPLEA YLKNTPVQKD SLTCLNWKQQ NTSASTSCFD T-AVTSASRT EMNPRG-NHK YARH--SDEG
B. rapa FPsc_GI          753  LMRCKWDKEI HKRASSLYNL IDVHSKVVAS IVDKAEPLEA YLKNAPVQKD SLACVNWKQQ NNTSSAAGFG TAAVTSTSRN EMAPRGGNHK YARH--SDEG
E. salsugineum_GI        759  LMRCKWDKEI HRRASSLYTL IDVHSKVVAS IVDKAEPLEA YLKNAPVQKD SLACLNWKQQ NTCASTTGFG T-AVTSTSRS EMTPRG-NHK YARH--SDEG
G. raimondii_GI          763  LMRCKWDNEI YTRASSLYNL IDIHSKAVAS IVNKAEPLEA QLIYAPVRKY SP-CLDDRKQ NKCSSATCFD PGQSSASECE DSTCSDNNLR SEKL--LASD
T. cacao_GI              763  LMRCKWDNEI YTRASSLYNL IDIHSKAVAS IVNKAEPLEA QLIHAPVWKD SPVCLDGRKQ NKRTNTTCFD PGQSSASECE DSTHSDKNLR CERV--LASD
C. sinensis_GI           758  LMRCKWDNEI YSRATSLYNL IDIHRKAVAS IVNKAEPLKA HLMHAPIWRD SIACSDCQKL HKCAKGGYFD PENASSSHCE ASDQPEIHLK SEGA--SCSD
M. esculenta_GI          766  LMRCKWDNEI YTRASSLYNL IDIHSKAVAS IVTKAEPLEA H-LQVPVWKD SLVCFDGKRQ NKKLSIRCFD SGQSSASQCV ESTHSDATII TERS--SCSK
R. communis_GI           756  LMRCKWDNEI YTRASSLYNL IDIHSKAVAS IVTKAEPLEA Y-LHVPVWRD SLVHFDCKKR NRSSSASCFD SGQSSASQRE ESAHSDSKIG TERL--QSG-
P. trichocarpa_GI        765  LMRCKWDNEI YTRASSLYNL IDVHSKAVAS IVNKAEPLGA H-LHAPVWKD SLVCSDGNKQ NRSATGCFN SGQSSALQST ELVHSETKLK CGRA--SHSE
S. purpurea_GI           764  LMRCKWDNEI YTRASSLYNL IDVHRKAVAS IVNQAEPLGA H-LHAPVWKD SPVCFDGNKQ NRSASTVCFN SGQSSVPQST KLVHSETKLK CERA--THTE
V. vinifera_GI           765  LMRCKWDEEI YTRASSLYNL IDIHSKAVAS IVNKAEPLEA HLIHATVWKD SPGHKDCSKE DDCASTSCFK SVNPLLLHSE DSAYSKSLPQ FEKA--PHLN
S. lycopersicum_GI       760  LIRCKWDNEI LSRASSLYNL IDIHSKVVAS IVDKAEPLEA HLIPVPVLKK RSSGLNGKKH NKYSNCTCLT AEQSSLLECK HSTDCKTLTM SEKVL-HSSE
M. guttatus_GI           759  LIKCKWDKEI HSRASSLFNL IDIHSKVVAS IVNKAEPLEA HLLNVPLS-- RANCFHGEKT DTCASCCRLE SGQPSSSSCE KLSGSEALVT REKS--QADG
A. coerulea_GI           764  LMRCKWDGEI YNRASSLYNL IDVHSKTVAS IVDKAEPLEA HLVHAPVWRD AASFSNGSKR INCSN-ASFT PDYSSTLEDA DDNASAHPAT SLKCK-KAID
S. bicolor_GI            755  MMRCKRDAEI STRASSLYHL IDLHGKTVSS IVNKAEPLEA HLTLTPVKKD NQHRCEENNT S-SSDSVKLE NKNGSTSHKK NGFSRPLLKC AEEVL-LNGD
Z. mays_GI               755  LMRCKRDAEI STRASSLYHL IDLHGKTVSS IVNKAEPLEA HLTLTPVKKD NQHRCEENNT N-SSDSAKLE NKNGST-HKK NGFSKPHLKC AEEVL-N-GN
S. italica_GI            755  LMRCKWDTGI STRASSLYHL IDLHGKTVSS IVNKAEPLEA HLTLTPVKKD NQQDSEENST S-SSDSGKLE NKNGSTSHKK NGFSRPLLKC AEEVR--NGN
P. virgatum_GI           755  LMRCKWDTEI STRASSLYHL IDLHGKTVSS IVNKAEPLEA HLTLTPVKRD NLQHSEGNNI S-SSDSGKME NKNGSTSHKK NGFSRPLLKC AEEVL-LNGD
O. sativa_GI             755  LKQCKWDAEI STRASSLYHL IDLHGKTVTS IVNKAEPLEA HLTLTPVKKD -EPPIEEKNI N-SSDGGALE KKDASRSHRK NGFARPLLKC AEDVI-LNGD
B. distachyon_GI         760  LKRCKWDAEI STRASSLYHL IDLHGKTVSS IVNKAEPLEA HLTLTSVRKD DGQHIEENNV S-SSDSDDLG KKNVLASHKK NGFSRPLLNC GEEAI-RNGG
```

```
A. trichopoda_GI      739  LMKCKWDNEI HTRASSLYHL IDIHGKAVAS IVDKAEPLEA NIVHASDRND APISFKGRKH IKSSS---FE EDNIPHEKTE STKESEEFLK SEKAN-FLTD
P. abies_GI           768  MMRCKWDSEL CARASTVLAL IDVNRKAVAS IIDKSESSVS HVQYRAENNY STDRAAYSGV GIHTEVAIDQ NHEGGNSLSL ENCHGQVEVK SHNGQEKKFM
S. moellendorffii_GI  723  IVRCKWDPGV SSKAASILAL VDGNDKAVEA VFNYADKLSG DEKRTLSSKQ AQKIISGFTK D--------- ---------- ---------- ----------

                                  910       920       930       940       950       960       970       980       990      1000
                           ....|....| ....|....| ....|....| ....|....| ....|....| ....|....| ....|....| ....|....| ....|....| ....|....|
M. truncatula_GI      868  EASGYNLGKG VTSFSLEASD LANFLTMDRH IGLNCNTQIF LISMLSEKQE LCFSVVSLLW HKLIASPETQ PCSESTSAQQ GWRQVVDALC NVVSASPAKA
G. max_GI             868  -ASDYTLGKG VTGFSLDASD LANFLTMDRH IGLNCNGQIF LRSTLAEKQE LCFSVVSLLW HKLIASPETQ PCAESTSAQQ GWRQVVDALC NVVSASPTKA
C. sativus_GI         861  VDLENTLGKR IASFQLDASE LANFLTIDRH IGFNGCAQIL LRSVLAEKQE LCFSVVSLLW HKLIATPEIQ PSAEGTSAQQ GWRQVVDALC NVVSASPAKA
M. domestica_GI       861  EGSGNTXGKG VASFPLDASD LANFLXKDRH IGFSCSAQVL LRSVLTEKQE LCFSVVSLLW YKLIAAPETQ PSAEXTSAQQ GWRQVVDALC NVVSATPKKA
F. vesca_GI           870  GGSG-TFGKG LANLPLDASE LANFLTMDRH IGFSCSAQVL LRTVLTEKQE LCFSVVSLLW HKLIASPETQ PTAESTSAQQ GWRQVVDALC NVVSATPTKA
A. thaliana_GI        860  SG--RPSEKG IKDFLLDASD LANFLTADRL AGFYCGTQKL LRSVLAEKPE LSFSVVSLLW HKLIAAPEIQ PTAESTSAQQ GWRQVVDALC NVVSATPAKA
A. lyrata_GI          861  SG--RPSEKG IKDFLLDASD LANFLTADRL AGFYCGTQKL LRSVLAEKPE LSFSVVSLLW HKLIAAPEIQ PTAESTSAQQ GWRQVVDALC NVVSATPAKA
C. rubella_GI         858  SG--RPSEKG IKEFLLDASD LANFLTADRL AGFYCGTQKL LRSVLAEKPE LSFSVVSLLW HKLIAAPEIQ PTAESTSAQQ GWRQVVDALC NVVSATPAKA
C. grandiflora_GI     858  SG--RPSEKG IKEFLLDASD LANFLTADRL AGFYCGTQKL LRSVLAEKPE LSFSVVSLLW HKLIAAPEIQ PTAESTSAQQ GWRQVVDALC NVVSATPAKA
B. rapa FPsc_GI       851  SGSRSSSEKG IKDLLLDASD LANFLTADRL AGFYRGTQVL LRSILAEKPE LSFSVVSLLW HKLIASPEIQ PTAESTSAQQ GWRQVVDALC NVVSATPAKA
E. salsugineum_GI     855  SGT-RHSEKG IKDFLLDASD LANFLTADRL AGFYCGTQVL LRSVLAEKPE LSFSVVSLLW HKLIAAPEIQ PTAESTSAQQ GWRQVVDALC NVVSATPAKA
G. raimondii_GI       860  EGLGNSLGKG IAGFPLDASD LANFLTRDRH IGFNCSAQIL LRSVLVEKQE LCFSVVSLLW HKLIAAPETQ PSAESTSAQQ GWRQVVDALC NVVSASPTKA
T. cacao_GI           861  EGSGNSLGKG IASFPLDASD LANFLTMDRH IGFNCSAQIL LRSVLVEKQE LCFSVVSLLW HKLIAAPETQ PSAESTSAQQ GWRQVVDALC NVVSASPTKA
C. sinensis_GI        856  ESSGNGLGKG IASFLVDASD LANFLTMDRH IGFNCSAQFL LRSVLAEKQE LCFSVVSLLW NKLIAAPETQ PSAESTSAQQ GWRQVVDALC NVVSASPTKA
M. esculenta_GI       863  EGSGSTLGKD IAAFPLDASD LANFLTMDRH IGFNFSAQVL LRTLLVEKQE LCFSVVSLLW HKMIAAPETQ PSAVSTSAQQ GWRQVVDALC NVVSASPTKA
R. communis_GI        852  EGSGSTLGNS IAGFPLDASD LANFLTMDRH IGFNCSAQVF LRSVLAKKQE LCFSVVSLLW HKLISAPETQ PSAESTSAQQ GWRQVVDALC NVVSATPTKA
P. trichocarpa_GI     862  EGSGSTSGKG IAGLPLDASD LANFLTMHRH IGFNCSAQVL LRSVLPEKQE LCFSVVSLLW QKLIASPETQ PSAESTSAQQ GWRQVVDALC NVVSASPTIA
S. purpurea_GI        861  EGSGSTSGKG IAGFPLEASD LANFLTMHRH IGFNCSAQVL LRSVLPEKQE LCFSVVSLLW QKLIASPETQ PSAESTSAQQ GWRQVADALC NVVSASPTIA
V. vinifera_GI        863  EGTGNSLGKG IASFPLDASE LANFLTMDRH IGFSCSAQVL LRSVLAEKQE LCFSVVSLLW HKLIAAPETK PSAESTSAQQ GWRQVVDALC NVVSASPAKA
S. lycopersicum_GI    859  AAQ-CTSGKG IASFPLDASD LANFLTMDRL IGFNCNAEDL IKSVLTENEE LCFSVVSLLW HKLIASPEIQ PSAESTSAQQ GWRQVIDALC NVVSALPAKA
M. guttatus_GI        855  VER-CSTGKE ISSFPIDASD LANFLTMDRH IGFNCSAQVL LKSVLSEKQE LCFSVVSLLW HKLIVSPEIQ LSGESTSAQQ GWRQVVDALV NVVSASPAKA
A. coerulea_GI        862  ANARNTHGKG TASLPTDASD LANLLTMDRH TGLNRSAQVL LKSVLPEKHE LCFSVVSLLW HKLIAAPETQ LSEEGTSAQQ GWRQVVDALC NVVSASPAKA
S. bicolor_GI         853  VAS--TSGKS IASLQVEASD LANFLTMDRN GGYRG-SQTL LRSVLSEKQE LCFSVVSLLW QKLIASPEMQ MSAESTSAHQ GWRKVVDALC DVVSASPTKA
Z. mays_GI            851  VAS--TSGKS IASLQVEASD LANFLTMDRN GGYRG-SQTL LRSVLSEKQE LCFSVVSLLW QKLIASPEMQ MSAESTSAHQ GWRKVVDALC DVVSASPTKA
S. italica_GI         852  VAS--TSGKA IASLQVEASD LANFLTMDRN GGYRG-SQTL LRSVLSEKQE LCFSVVSLLW QKLIASPEMQ MSAESTSAHQ GWRKVVDALC DVVSASPTKA
P. virgatum_GI        853  VAS--TSGKA IASLQVEASD LANFLTMDRN GGYRG-SQTL LRSVLSEKQE LCFSVVSLLW QKLIASPEMQ MSAESTSAHQ GWRKVVDALC DVVSASPTKA
O. sativa_GI          852  VAS--TSGKA IASLQVEASD LANFLTMDRN GGYRG-SQTL LRSVLSEKQE LCFSVVSLLW QKLIASPEMQ MSAESTSAHQ GWRKVVDALC DIVSASPTKA
B. distachyon_GI      858  VAS--TSGKV IATLQVEASD LANFLTMDRN GGYGG-SQTL LRSVMSEKQE LCFSVVSLLW QKLIASPEMQ MSAESTSAHQ GWRKVVDALC DVVSASPAKA
A. trichopoda_GI      835  VMLS-GSGKS IAGVTVDASD LANFLTMDRY LGINCNAQSL LRSFLAEKQE LCFAVVSLLW HKLIATPETQ MIIESTSAQQ GWRQVADALC NVVVASPAKA
P. abies_GI           868  DVVIAGSGKV IANLSMDGSD VVSLLTVDRH KWVNHNVGNF VTAVLEEKQD LCVASVPLLW QRLITAPEMQ MSVESTSAKQ GWRQVVDALC NIVLASPAKA
S. moellendorffii_GI  783  -------GVK DASLSLNASD VTNLLCG--C NGVSTTVSDL LKAVLKQKRD LAVAIVPLLW QRLMSAEELP TSKEGTSAKQ GWRQVVDAVC NVVLTYPEKA

                                 1010      1020      1030      1040      1050      1060      1070      1080      1090      1100
                           ....|....| ....|....| ....|....| ....|....| ....|....| ....|....| ....|....| ....|....| ....|....| ....|....|
M. truncatula_GI      968  ATAVVLQAEK ELQPWIAKDD DLGQKMWRIN QRIVKLIVEL MRNHDSAESL VILASASDLL LRATDGMLVD GEACTLPQLE LLEATARAIQ PVLEFGEPGL
G. max_GI             968  ATAVVLQAER ELQPWIAKDD DSGQKMWRIN QRIVKLIVEL MRNHETAESL VIVASSSDLL LRATDGMLVD GEACTLPQLE LLEATARAVQ PVLEFGESGL
C. sativus_GI         961  AAAVVLQADR EFQPWIAKDD NQGQKMWRIN QRIIKLIVEL MRNHDKPESL VTLASASDLL LRATDGMLVD GEACTLPQLE LLEATARAVR PVLEWGESGL
M. domestica_GI       961  ATAXVLQAER ELQPWIAKDD DQGQKMWRIN QRIVKLIVEL MRIHDSPESL VILSSASDLL LRATDGMLVD GEACTLPQLE LLEATARAIQ PMLEWGESGL
F. vesca_GI           969  ATAVVLQAER ELQPWIAKDD DQGQKMWRIN QRIVKLIVEL MRIHDSPESL VILSSASDLL LRATDGMLVD GEACTLPQLE LLEATARAVK PVLEWGESGL
A. thaliana_GI        958  AAAVVLQAER ELQPWIAKDD EEGQKMWKIN QRIVKVLVEL MRNHDRPESL VILASASDLL LRATDGMLVD GEACTLPQLE LLEATARAIQ PVLAWGPSGL
A. lyrata_GI          959  AAAVVLQAER ELQPWIAKDD EEGQKMWKIN QRIVKVLVEL MRNHDRPESL VILASASDLL LRATDGMLVD GEACTLPQLE LLEATARAIQ PVLAWGPSGL
C. rubella_GI         956  AAAVVLQAER ELQPWIAKDD EEGQKMWKIN QRIVKVLVEL MRNHDRPESL VILASASDLL LRATDGMLVD GEACTLPQLE LLEATARAIQ PVLAWGPSGL
C. grandiflora_GI     956  AAAVVLQAER ELQPWIAKDD EEGQKMWKIN QRIVKVLVEL MRNHDRPESL VILASASDLL LRATDGMLVD GEACTLPQLE LLEATARAIQ PVLAWGPSGL
B. rapa FPsc_GI       951  AAAVVLQAER ELQPWIAKDD EEGQKMWKIN QRIVKVMVEL MRNHDRPESL VILASASDLL LRATDGMLVD GEACTLPQLE LLEATARAIQ PVLAWGPSGL
E. salsugineum_GI     954  AAAVVLQAER ELQPWIAKDD EEGQKMWKIN QRIVKVMVEL MRNHDRPESL VILASASDLL LRATDGMLVD GEACTLPQLE LLEATARAIQ PVLAWGPSGL
G. raimondii_GI       960  TTAVVLQADR ELQPWIAKDD DQGQKMWRIN QRIVKLIVEL MRNHDSAESL VIVASASDLL LRATDGMLVD GEACTLPQLE LLEATARAVQ PVLEWGESGL
T. cacao_GI           961  ATAVVLQAER EFQPWITKDD DQGQKMWRIN QRIVKLIVEL MRNHDSPESL VIVASASDLL LRATDGMLVD GEACTLPQLE LLEATARAVQ PVLEWGESGL
C. sinensis_GI        956  ATAVVLQAER ELQPWIAKDD DQGQKMWRIN QRIVKLIVEL MRIYDSPESL VILASASDLL LRATDGMLVD GEACTLPQLE LLEATARAIQ PILRWGKSGL
M. esculenta_GI       963  ATAIVLQAER ELQPWIAKDD DQGQKLWRIN QRIVRLMVEL MRNHDTPESL VILASASDLL LRATDGMLVD GEACTLPQLE LLEATARAVQ PVLEWGESGF
R. communis_GI        952  AAAVVLQAEK ELQPWIAKDD DQGQKMWRIN QRIVRLIVEL MRNHDTPESL VILASASDLL LRATDGMLVD GEACTLPQLE LLEATARAVQ PVLEWGESGF
P. trichocarpa_GI     962  ATAVVLQAER ELQPWIAKDD DSGQIMWRIN QRIVKLIVEL MRNHDTPESL VILASASDLL LRATDGMLVD GEACTLPQLE LLEATARAVQ PVLQWGESGF
S. purpurea_GI        961  ATAVVLQAER ELQPWISKDD DSGQKMWRIN QRIVKLIVEL MRNHDNPESL VILASASDLL LRATDGMLVD GEACTLPQLE LLEATARAVK PVLQWGESGF
V. vinifera_GI        963  ATAVVLQAER ELQPWIAKDD DLGQKMWRIN QRIVKLIVEL MRNHDRPESL VILSSASDLL LRATDGMLVD GEACTLPQLE LLEATARAVQ LVLEWGESGL
S. lycopersicum_GI    958  ATAIVLQADK ELQPWIAKDD DLGQKMWRIN QRIVKLIAEV MRNHDTPESL VILASAPDLL LRATDGMLVD GEACTLPQLE LLEVTARAVQ PVLEWGESGS
M. guttatus_GI        954  ATAVVLQADR ELKPWITKDD DLGQKMWKVN QRIVKVIVEL MRNHDAPESL VILSSASDLL LRATDGMLVD GEACTLPQLE LLEVTARAVQ TVLKWGESGL
A. coerulea_GI        962  SAAIVLQAEK DLQPWIARDD EQGQRMWKIN QRIVQLIVEL MRNHDTPESL VILASASDLL LRATDGMLVD GEACTLPQLE LLEATAKAVQ LVVKWGDSSS
S. bicolor_GI         950  STAIVLQADK DLQPWIARDD EQGQKMWRVN QRIVKLIAEL MRNHDSPEAL VILASASDLL LRATDGMLVD GEACTLPQLE LLEVTARAVH LIIEWGDSGL
Z. mays_GI            948  SAAIVLQAEK DLQPWIARDD EQGQKMWRVN QRIVKLIAEL MRNHDSPETL VILASASDLL LRATDGILVD GEACTLPQLE LLEVTARAVH LIIEWGDPGL
S. italica_GI         949  SAAIVLQADK DLQPWIARDD EQGQKMWRVN QRIVKLIAEL MRNHDSPEAL VILASASDLL LRATDGMLVD GEACTLPQLE LLEVTARAVH LIIEWGDSGL
P. virgatum_GI        950  STAIVLQAEK DLQPWIARDD EQGQKMWRVN QRIVKLIAEV MRNHDSPEAL VILASASDLL LRATDGMLVD GEACTLPQLE LLEVTARAVH LIIEWGDSGL
O. sativa_GI          949  SAAIVLQAEK DLQPWIARDD EQGQKMWRVN QRIVKLIAEL MRNHDSPEAL VILASASDLL LRATDGMLVD GEACTLPQLE LLEVTARAVH LIVEWGDSGV
B. distachyon_GI      955  STAIVLQAEK DLQPWIARDD EQGQKMWRVN QRIVKLIAEL MRNHDSPEAL VILASASDLL LRATDGMLVD GEACTLPQLE LLEVTARAIH LIVEWGDPGV
A. trichopoda_GI      934  STAVVLQAER DLQPWIARDD GQGHEMWRIN QRIVHLVAEL LRNHDAPEAL MILASASDLL LRATDGMLVD GEACTLPQLE LLEATARAVQ LFLSWGESGS
P. abies_GI           968  ATAIVVQAER DLQPWVARHD SQGQQIWRLN QRIVSLLAEL LRYHNAPEAL MVLANASDLL MRATDGMLVD GEACTTPQLE LLEAMAVTAQ LSLGWGVPGK
S. moellendorffii_GI  875  TSVVLLQAER GIQPWIIGDG --GEEKWRMN TRIVFLLSEL LRLND-PQVL GLIANAGTLL YQATDGMSVD GEPCTLPQLE LLEAIAMAIK SLCAWKVS--

                                 1110      1120      1130      1140      1150      1160      1170      1180      1190      1200
                           ....|....| ....|....| ....|....| ....|....| ....|....| ....|....| ....|....| ....|....| ....|....| ....|....|
M. truncatula_GI     1068  AVADGLSNLL KCRLAATIRC LCHPSAHVRA LSVSVLRDIL HTGSIR---C SPKP-----L RINGSHNPSY QYFKLDVVDW QADIEKCMAW EAHSRISAGL
G. max_GI            1068  AVADGLSNLL KCRLSATIRC LSHPSAHVRA LSISVLRDIL HTGSIR---C SPKP-----R RLNGTHNPSY QYFNLDAVDW QADIEKCLTW EAHSRLSNGL
C. sativus_GI        1061  SIADGLANLL KCRLPATVRC LSHPSAHVRA LSTSVLRDIL QTGSYV---H RSTP-----N NINGVHSPSF QYFNREAINW KVDLEKCLTW EAHSRLVTGM
M. domestica_GI      1061  AVADGLSNLL KCRLPATIRC LSHPSAHVRA LSTSVLRDIL QASSIR---P NPNP-----V EIDGIHGPXY KYFNLDVIDW QGXIEKCLTW XAHSRLATGM
F. vesca_GI          1069  AVADGLSNLL KCRLSATIRC LSHPSAHVRA LSVSVLRDIL QTSSVR---P NPNP-----V QINGIHGPSY KYFNLDVIDW QADIEKCLTW EAHSRLATGM
A. thaliana_GI       1058  AVVDGLSNLL KCRLPATIRC LSHPSAHVRA LSTSVLRDIM NQSSIP-IKV TPK---LPTT EKNGMNSPSY RFFNAASIDW KADIQNCLNW EAHSLLSTTM
A. lyrata_GI         1059  AVVDGLSNLL KCRLPATVRC LSHPSAHVRA LSISVLRDIM NQSSIP-IKV TPK---LPTT EKNGMNSPSY RFFNAATIDW KADIQKCLNW EAHSLLSTTM
C. rubella_GI        1056  AVVDGLSNLL KCRLPATIRC LSHPSAHVRA LSTSVLRDIM NQSSIP-IKV TPK---LPTT EKNGMHSPSY RFFNAATIDW KADIQKCLNW EAHSLLSTTM
C. grandiflora_GI    1056  AVVDGLSNLL KCRLPATIRC LSHPSAHVRA LSTSVLRDIM NQSSIP-IKV TPK---LPTT EKNGMHSPSY RFFNAATIDW KADIQKCLNW EAHSLLSTTM
B. rapa FPsc_GI      1051  AVVDGLSNLL KCRLPATIRC LSHPSAHVRA LSTSVLRDIM NQSGITTTKA TPKPPPTITT EKNGTDSPSY RFFNAAAIDW KADIQKCLNW EAHSLLSTTM
E. salsugineum_GI    1054  AVVDGLSNLL KCRLPATIRC LSHPSAHVRA LSTSVLRDIM NQSSVT-ITV TPKQ--RPAT EKNVMHSPSY RFFNAATIDW KADIQKCLNW EAHSLLSTTM
G. raimondii_GI      1060  AVADGLSNLL KCRLPATTRC LSHPSAHVRA LSTSVLRNIL HIGSIN---S KSK------L EIYGIRGPSY QFFNIGAINW QTDIEKCLTW EVYSQLARGM
T. cacao_GI          1061  AVADGLSNLL KCRLPATTRC LSHPSAHVRA LSTSVLRNIL HAGSIK---P NSKQ-----V EINGIHGPSY QYFSVGVIDW HTDIEKCLTW EAHSQLARGM
C. sinensis_GI       1056  AIADGLSNLL KCRLPATIRC LSHPSAHVRA LSTSVLRDFL HTSSFK---S NIEQ-----V ERNGIHGSSL HYFNIDAINW QSDIEKCLTW EAHSRLATGM
M. esculenta_GI      1063  AVADGLSNLL KCRLPATIRC LSHPSAHVRA LSTSVLRSIL HTCSIK---P TANQ-----A DINDIRGPSY QLFKVDVIDW QADIEKCLTW EAHSRLATGM
R. communis_GI       1052  AVADGLSNLL KCRLPATIRC LSHPSAHVRA VSTSVLRGIL YTGSIK---R TSNR-----V DINGIRGPSY QYFNIDVTDW QTDIEKCLTW EAHSRLATGM
P. trichocarpa_GI    1062  AVADGLSNLL KCRLPATIRC LSHPSAHVRA LSTSVLRDIQ HTGSIK---P ASKL-----T HRNGIHGPSY QYLRSDVINW QADIEKCLTW EAHSRLATGM
S. purpurea_GI       1061  AVADGLSNLL KCRLSATIRC LSHPSAHVRA LSTSVLRDIQ HTGSIK---P VSNL-----T HRNGIHG-SH PYFRSDVINW QADIEKCLIW EAHSRLVAGM
V. vinifera_GI       1063  AVADGLSNLL KCRVPATIRC LSHPSAHVRA LSTSVLRDVL QSGSIK---P HIKQ-----G GRNGIHS--Y QYVNLGIIDW QADIEKCLTW EAHSRLATGM
S. lycopersicum_GI   1058  VIVDGLSNLL KCRLPATVRC ISHPSALVRA LSISLLRAIM QTGSIK---- TRAN----RA DVNGIHGPAY KYLNIGTINW QRDIEKCLTW EANSRIENGM
M. guttatus_GI       1054  AVADGLSNLL KCRLPATVRC VSHPSAHVRA LSTSVLRAVL HAGSES---- ISA-----KS QVNGFR--SQ PYISVGIRDC KAHVEKCLTW EAHSRLATGL
A. coerulea_GI       1062  AITDGLSNLL KCRLPATVRC LSHPSAHVRA LSTSVLRDIL FLGPIK---F NVKQ----EK RYSGVQGPSH RCLSSSIIDW NKDIEKCLTW EARSRHTTGV
S. bicolor_GI        1050  SVADGLCNLL KCRLSTTIRC LSHPSAHVRA LSMSVLRDIL NNGSMNPSKI IQGE-----Q QRNGIQNPSY RCLAAGIINW QADVERCIEW EAHSRRATGL
Z. mays_GI           1048  SVADGLSNLL KCRLSTTIRC LSHPSAHVRA LSMSVLRDIL DHGSVSPNKI SRGE-----Q QRNGIQSPSY RCVAAGILNW QADVERCIEW EAHSRRATGL
S. italica_GI        1049  SVADGLSNLL KCRLSPTIRC LSHPSAHVRA LSMSVLRDIL NNGHITPSKI IQGE-----Q QRNGIQNSSY RCLAASIN-W QADVERCIEW EAHSRRATGL
P. virgatum_GI       1050  SVADGLSNLL KCRLSPTIRC LSHPSAHVRA LSMSVLRDIL NNGHINPSKI IQGE-----Q QRNGIQSSSY RCLAASIN-W QADVQRCIEW EAHSRRATGL
O. sativa_GI         1049  SVADGLSNLL KCRLSTTIRC LSHPSAHVRA LSMSVLRDIL NSGQINSSKL IQG------E HRNGIQSPTY QCLAASIINW QADVERCIEW EAHSRRATGL
```

```
B. distachyon_GI      1055 AVADGLSNLL KCRLSTTIRC LSHPSAHVRA LSMSVLRDIL NSGPINSTKI IQG------E QRNGVQSPTY QCAAASMVNW QADMERCIEW EARSRRATGM
A. trichopoda_GI      1034 SVADGLSNLL KCRLPATIHC LSHPSAHVRA LSTSLLRDIL NIGSPK---S NFAE-----E DIRGIPVPPY RNISIGTIDW RSDLEKCLAW EARNRRATGM
P. abies_GI           1068 AMAEGLWNLL KYRLPATVQC LSHSSAHVRA LSTSVLRDIL HAESLN--FR YCKN----FS EKNHHSEHLY YGKDIVVQDW NKAVEQCLAW EAHNRQARGM
S. moellendorffii_GI  969  --SRGLLILL KERLPAIVRC LSHDSPRIRA SSASLLREIV STDVLR---- ---------- ---------A SYPGDKAGAW LEDVEQSIAW ETHYRRAEGL
```

```
                                  1210       1220
                           ....|....| ....|....| .
M. truncatula_GI      1160 PIKFLDTAAK ELGCAISV-- -
G. max_GI             1160 SINFLDIAAK ELGCTISM-- -
C. sativus_GI         1153 PIEVLHVAAK ELGCSISL-- -
M. domestica_GI       1153 QIKFLDTAAK ELGCSISL-- -
F. vesca_GI           1161 PIKFLDTAAK ELGCTISV-- -
A. thaliana_GI        1154 PTQFLDTAAR ELGCTISLSQ -
A. lyrata_GI          1155 PTQFLDTAAR ELGCTISLSQ -
C. rubella_GI         1152 PTQFLDTAAQ ELGCTISLSQ -
C. grandiflora_GI     1152 PTQFLDTAAQ ELGCTISLSQ -
B. rapa FPsc_GI       1151 PTQFLDTAAR ELGCTISMSS Q
E. salsugineum_GI     1151 PTQFLDTAAR ELGCTISLSS Q
G. raimondii_GI       1151 TIQFLDTAAK ELGCNISI-- -
T. cacao_GI           1153 PIRFLDTAAK ELGCSISI-- -
C. sinensis_GI        1148 PIQFLDIAAK ELGCTISI-- -
M. esculenta_GI       1155 AIQFLETAAK ELGCTVSI-- -
R. communis_GI        1144 PIQFLDTAAK ELGCTISI-- -
P. trichocarpa_GI     1154 PVHHLDTAAK ELGCTISI-- -
S. purpurea_GI        1152 SVHHLDTAAK ELGCTISI-- -
V. vinifera_GI        1153 TNQFLDVAAK ELGCTISI-- -
S. lycopersicum_GI    1150 CTQFLDMAAK ELGCTISI-- -
M. guttatus_GI        1143 PIQFVDTTAK ELGCTICI-- -
A. coerulea_GI        1155 STTFLDAAAK DLGCTILI-- -
S. bicolor_GI         1145 TLAFLSAAAK ELGCPLPC-- -
Z. mays_GI            1143 TLAFLSTAAK ELGCPLPC-- -
S. italica_GI         1143 TLAFLSAAAK ELGCPLPC-- -
P. virgatum_GI        1144 TLAFLSAAAK ELGCPLPC-- -
O. sativa_GI          1143 TLAFLTAAAK ELGCPLTC-- -
B. distachyon_GI      1149 TLAFLSAAAN ELGCPLPC-- -
A. trichopoda_GI      1126 TLTFLSAAAK ELGCAISV-- -
P. abies_GI           1162 SIALLALAAN ALGFSANV-- -
S. moellendorffii_GI  1045 SESFLASAAI ALGCKLPPS- -
```

Supplementary Fig. 1. A phylogenetic tree and alignments of GI orthologs.
(a) Evolutionary relationships of taxa used in the GI alignment. The evolutionary history was inferred using the Neighbor-Joining method [1]. The optimal tree with the sum of branch lengths = 2.88320250 is shown. The tree is drawn to scale, with branch lengths in the same units as those of the evolutionary distances used to infer the phylogenetic tree. The evolutionary distances were computed using the Poisson correction method and are in the units of the number of amino acid substitutions per site. The analysis involved 31 amino acid sequences. All ambiguous positions were removed for each sequence pair. There were a total of 1220 positions in the final dataset. Evolutionary analyses were conducted in MEGA6 [2]. The scale bar corresponds to an evolutionary distance of 0.05 substitution per amino acid position. (b) Multiple alignment of GI orthologs obtained from ClustalW implemented in MEGA. Identical and similar amino acid residues are indicated by black and gray shading, respectively.

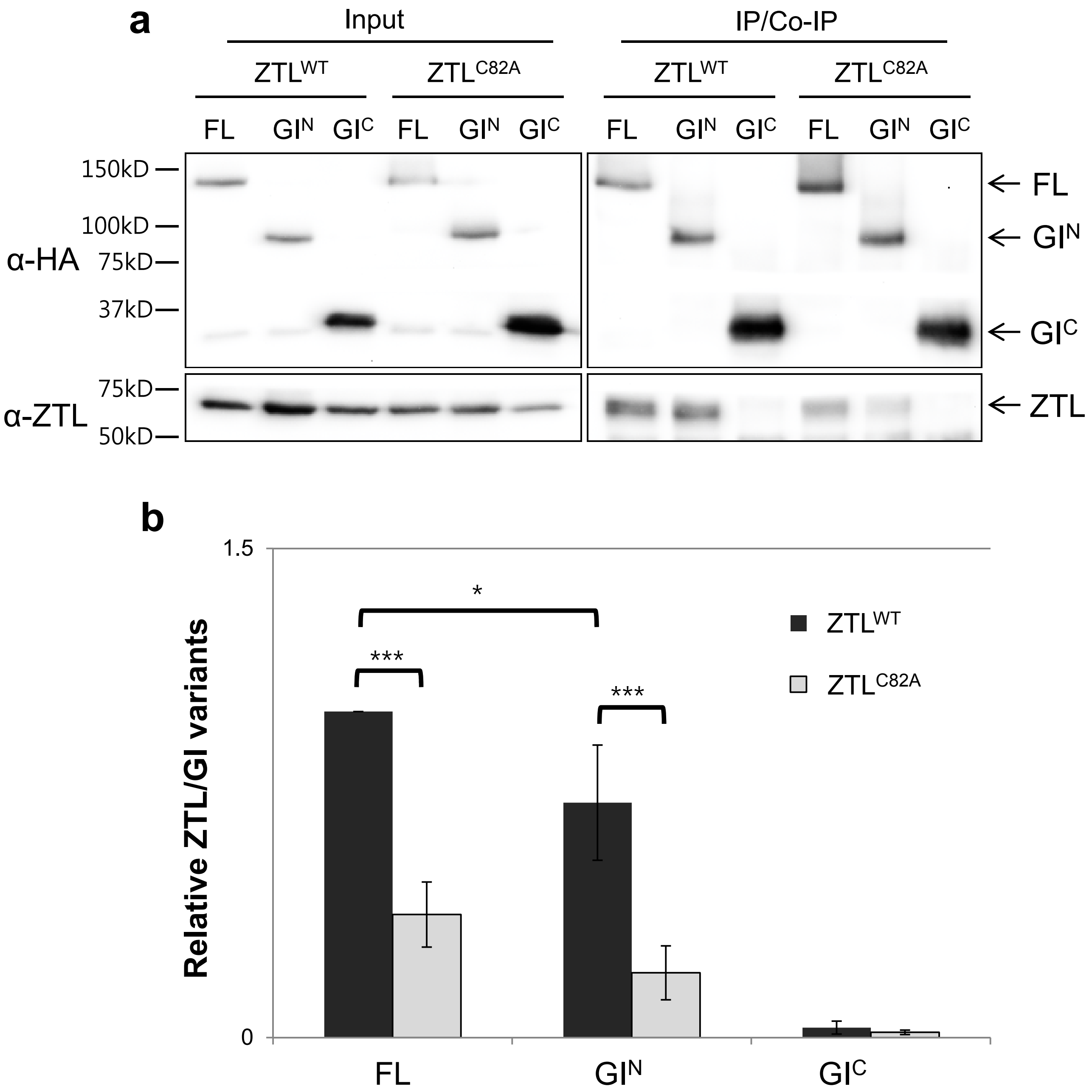

Supplementary Fig. 2. GI^{N} recapitulates full-length GI specificity of ZTL interaction *in planta*. (a) HA-tagged GI full length (FL), GI^{N} and GI^{C} were separately co-infiltrated with wild type (WT) ZTL or photochemically dead ZTL^{C82A} in *N. benthamiana*. Immunoprecipitation was performed with anti-HA antibody, followed by immunoblotting and probing for GI (anti-HA) and ZTL (anti-ZTL). **(b)** Quantification of densitometric ratio of co-immunoprecipitated ZTL to immunoprecipitated GI. Relative to WT ZTL, GI^{N} shows significantly poorer interaction with ZTL^{C82A} , similar to that of FL GI. Error bars indicate s.e. (n=9). For t-test: 0.01<*<0.05, ***<0.001.

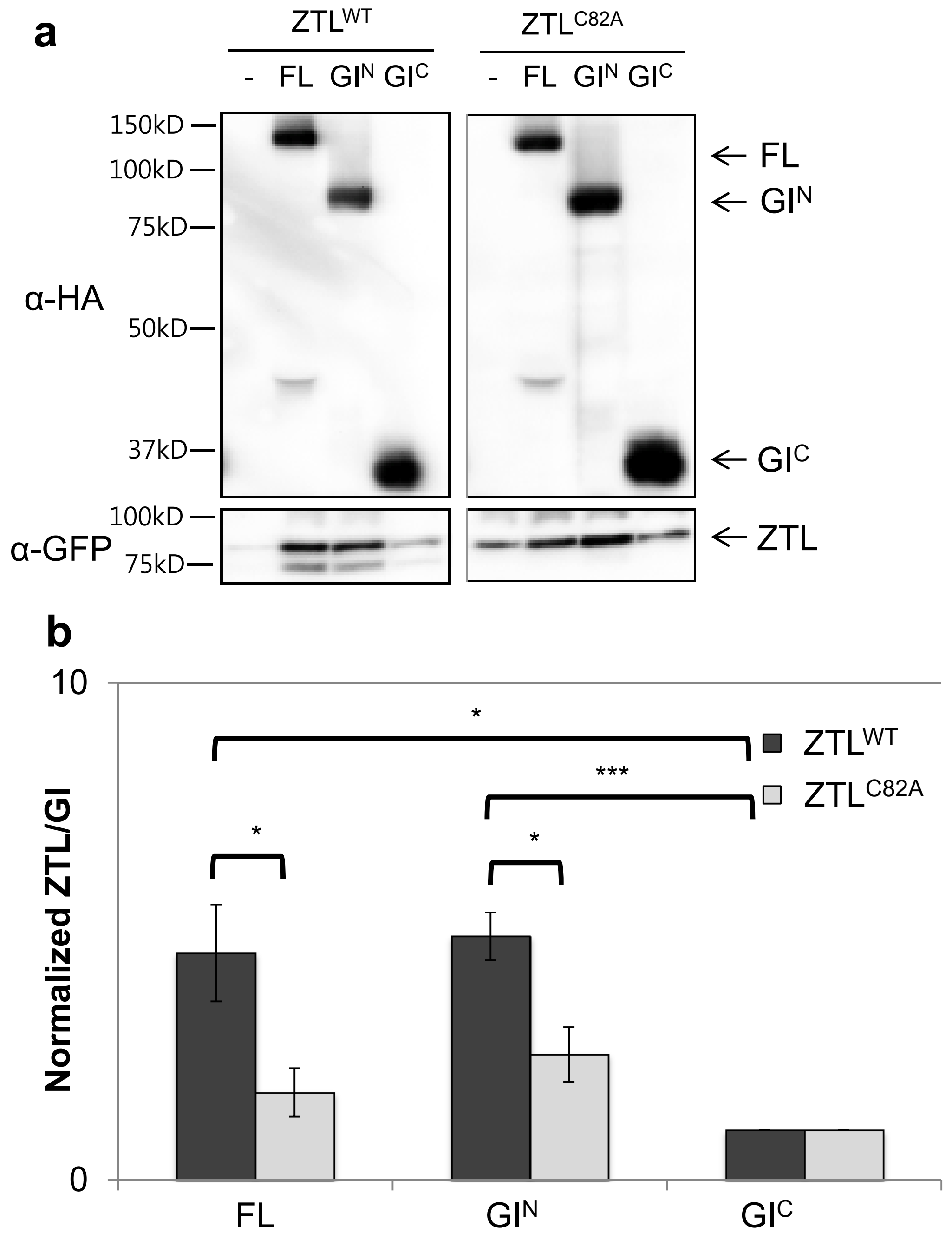

Supplementary Fig. 3. GI^N recapitulates full-length GI stabilization of ZTL *in planta*.
(a) HA-tagged GI full length (FL), GI^N, and GI^C were separately co-transformed with GFP-tagged wildtype (WT) ZTL or ZTL^{C82A} into *A. thaliana gi-201* protoplasts. Stabilization of ZTL is seen as increased band intensity when co-transformed with GI-FL or GI^N, but not with GI^C. Immunoblots were performed with anti-HA antibody and anti-GFP antibody. **(b)** Quantification of densitometric ratio of ZTL to GI variants. Values normalized to GI^C. Error bars indicate s.e. (n=6 for WT ZTL, and n=4 for C82A ZTL). For t-test: 0.01<*<0.05, ***<0.001.

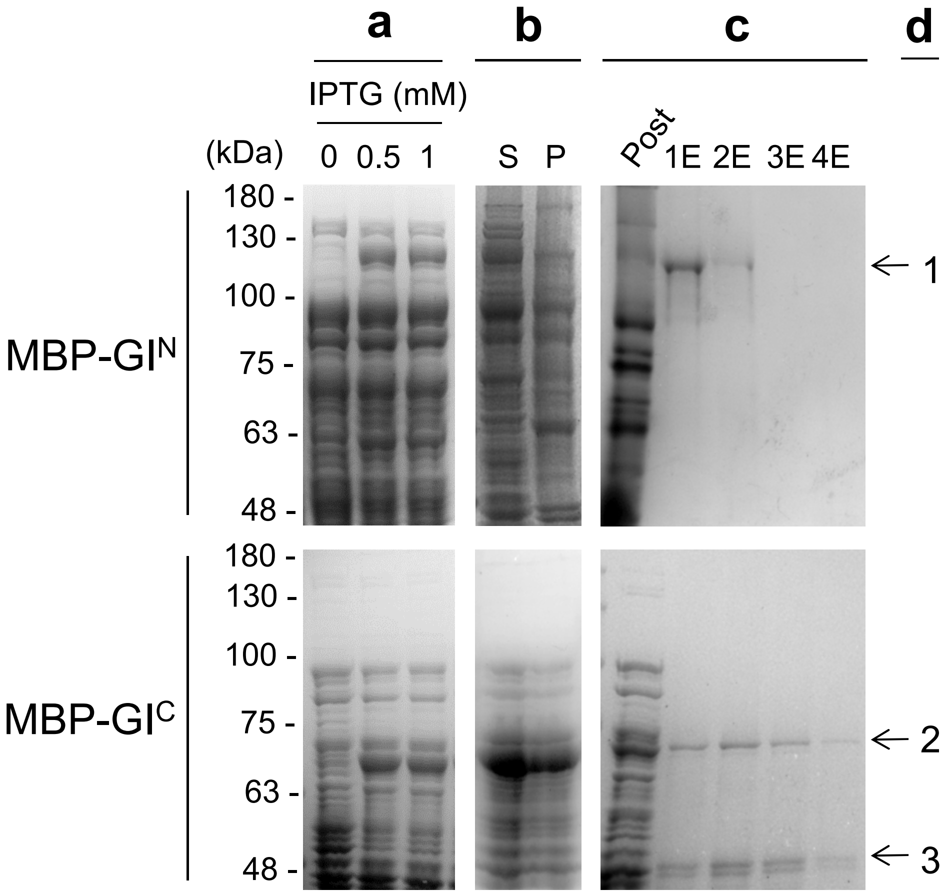

Supplementary Fig 4. Expression, purification and identification of MBP-GI^{N} and MBP-GI^{C}. **(a)** IPTG-induced expression of MBP-GI in *E. coli.* **(b).** Soluble fractionation by centrifugation. S, supernatant; P, pellet fraction. **(c)** Affinity chromatography using amylose resin. Post, post binding supernatant. 1E-4E, each eluates using 10 mM maltose elution buffer. (**d**) First eluate (1E) of each bands analyzed by MALDI-TOF/TOF MS.

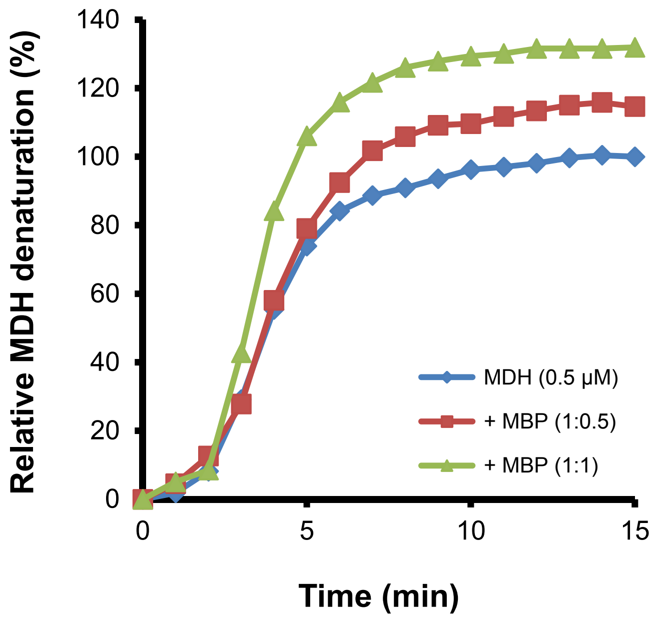

Supplementary Fig. 5. MBP does not protect MDH under heat denaturation.
The aggregation of MDH (0.5 μM) was monitored (turbidity at 340 nm) under thermal denaturing conditions for 15 min at 45 °C in the absence or presence of MBP at different molar ratios.

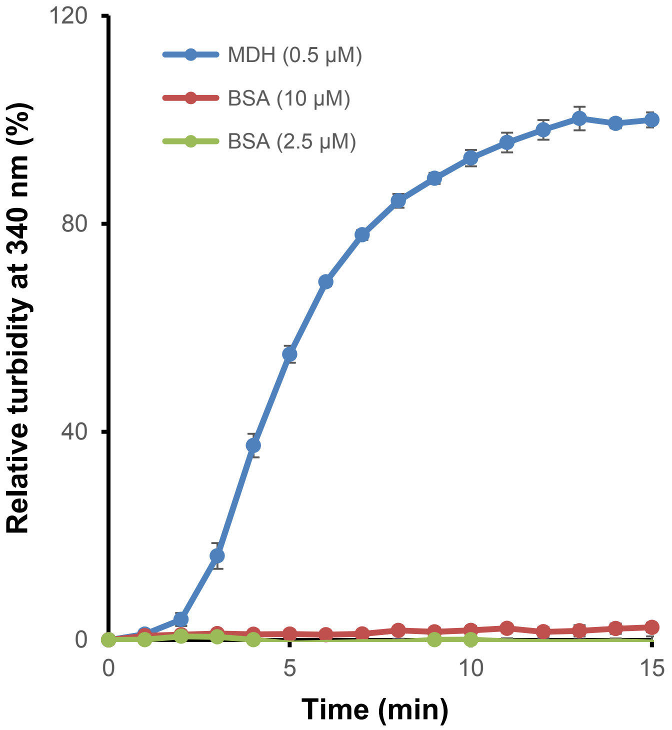

Supplementary Fig. 6. BSA alone is heat-stable compared to MDH under heat denaturation. Ten or 2.5 μM BSA, corresponding to Fig. 1a-c and Fig. 2a-c, was tested relative to the aggregation of MDH (0.5 μM) under thermal denaturing conditions for 15 min at 45 °C (turbidity at 340 nm). Data are means± s.e (*n*=3).

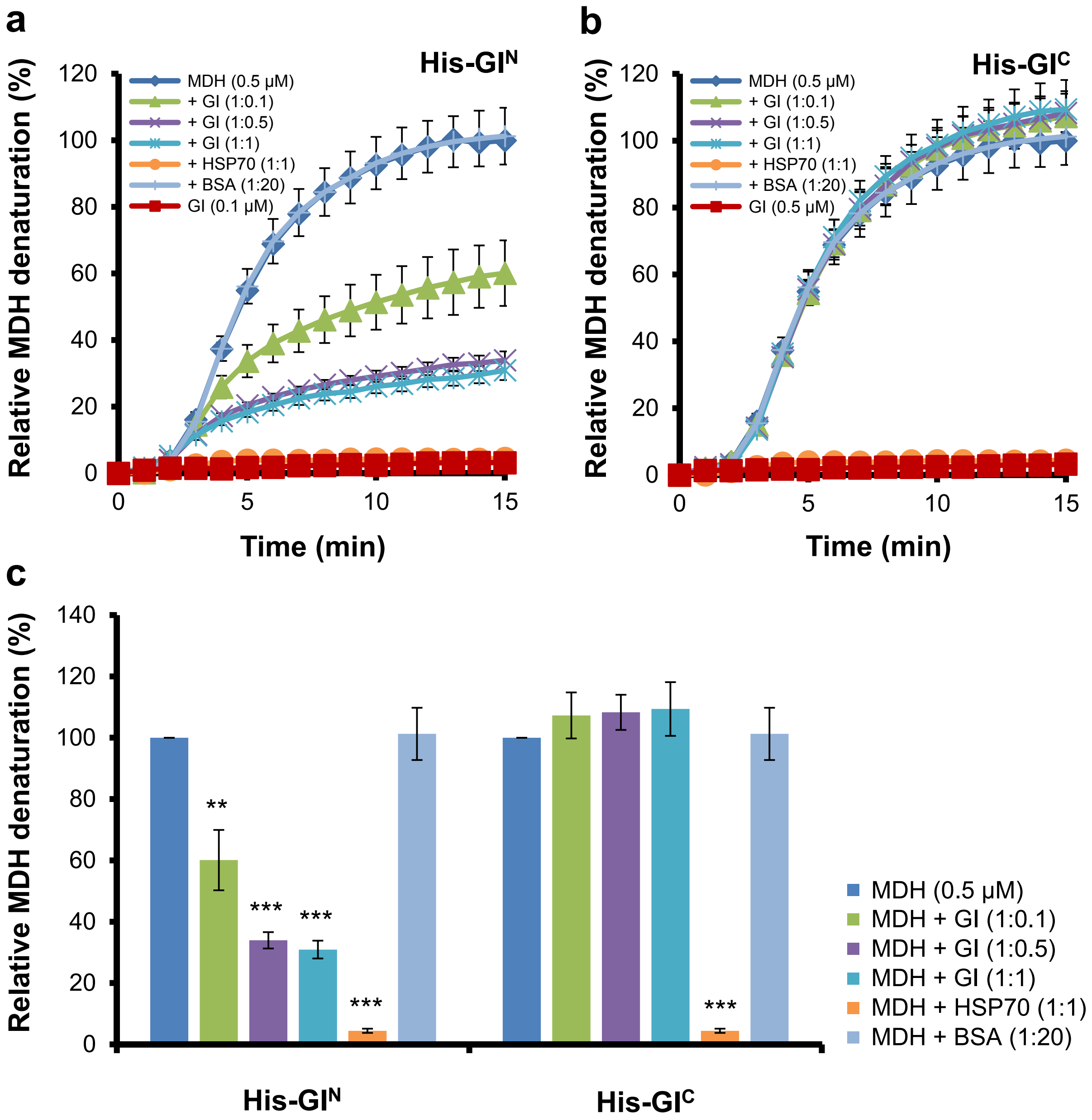

Supplementary Fig. 7. His-GI^N exhibits general holdase chaperone activity *in vitro*. Recombinant His-GI^N or His-GI^C was expressed and purified from *E. coli* BL21 (DE3)**.** The aggregation of MDH (0.5 µM) was monitored by measuring the turbidity at 340 nm under thermal denaturing conditions for 15 min at 45 °C. The turbidity of MDH alone at 15 min was set to 100%, and that from each treatment expressed relative to it. HSP70 and BSA used as positive and negative controls, respectively. **(a)** His-GI^N effectively protects heat-induced MDH aggregation as similar as MBP-GI^N. **(b)** His-GI^C does not display chaperone activity *in vitro*. **(c)** The mean denaturation rate at the treatment endpoint (15 min) of (a) and (b) relative to thermal-denaturation of MDH alone. * $P < 0.05$; ** $P < 0.01$; *** $P < 0.001$; two-tailed Student's t-test. Data are means ± s.e. (*n*=3).

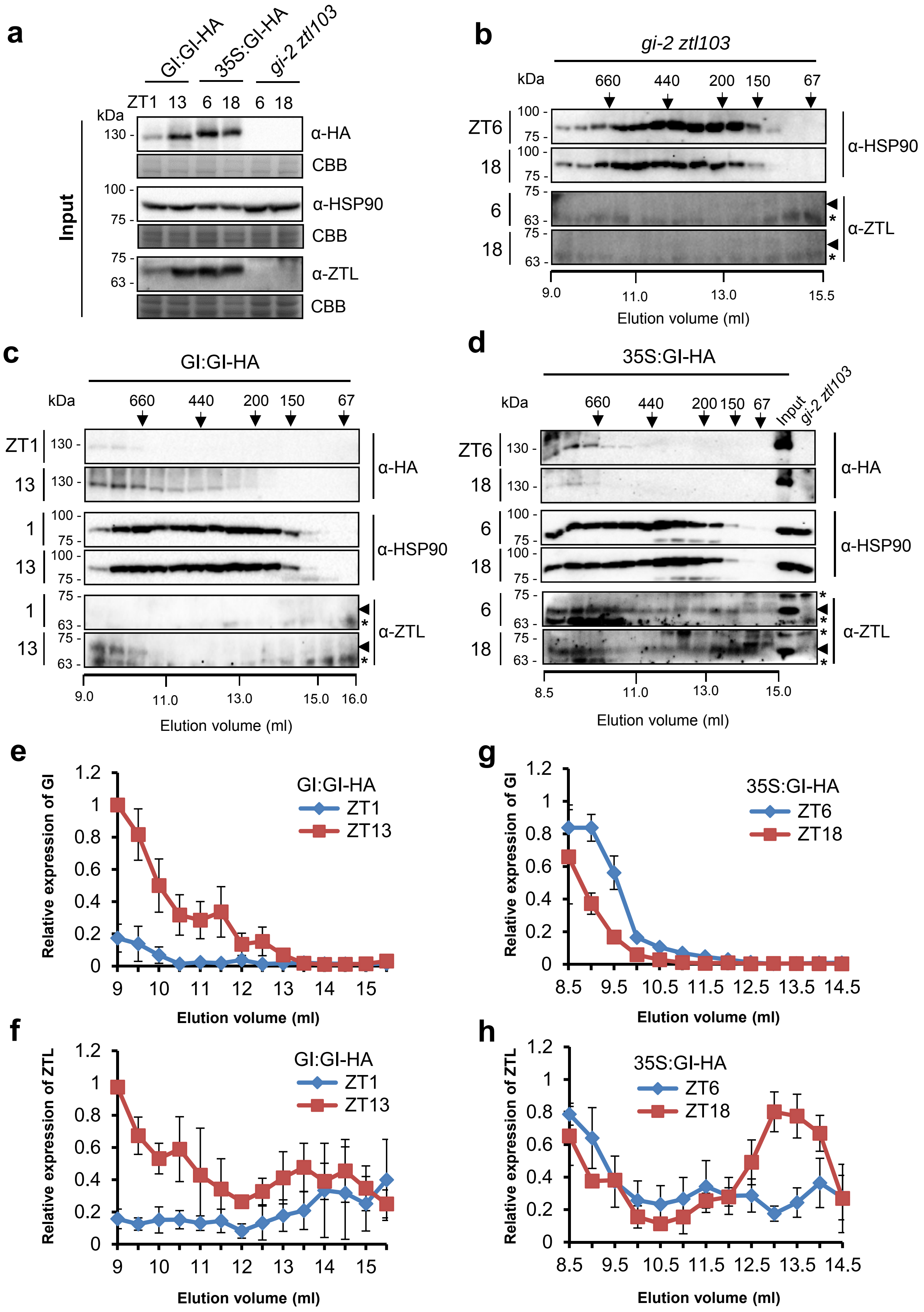

a
GI:GI-HA
35S:GI-HA
gi-2 ztl103
ZT1 13 6 18 6 18
kDa
Input
130 -
α-HA
CBB
100 -
75 -
α-HSP90
CBB
75 -
63 -
α-ZTL
CBB
b
gi-2 ztl103
kDa
660 440 200 150 67
ZT6
18
6
18
α-HSP90
α-ZTL
9.0 11.0 13.0 15.5
Elution volume (ml)
c
GI:GI-HA
kDa
660 440 200 150 67
ZT1
13
1
13
1
13
α-HA
α-HSP90
α-ZTL
9.0 11.0 13.0 15.0 16.0
Elution volume (ml)
d
35S:GI-HA
kDa
660 440 200 150 67
Input
gi-2 ztl103
ZT6
18
6
18
6
18
α-HA
α-HSP90
α-ZTL
8.5 11.0 13.0 15.0
Elution volume (ml)
e
Relative expression of GI
GI:GI-HA
ZT1
ZT13
Elution volume (ml)
f
Relative expression of ZTL
GI:GI-HA
ZT1
ZT13
Elution volume (ml)
g
Relative expression of GI
35S:GI-HA
ZT6
ZT18
Elution volume (ml)
h
Relative expression of ZTL
35S:GI-HA
ZT6
ZT18
Elution volume (ml)

Supplementary Fig. 8. GI , ZTL and HSP90 co-elution in large complexes is light-dependent. (a) Input and **(b-d)** protein migration profiles of GI-HA, ZTL and HSP90 proteins in **(b)** *gi-2 ztl-103* (ZT6 and ZT18), **(c)** *GI:GI-HA* (ZT1 and ZT13) and **(d)** *35S:GI-HA* (ZT6 and ZT18) seedlings grown under 12 h L/12 h D. **(a)** shows the expressions of GI-HA, HSP90 and ZTL in independent lines before gel-filtration. CBB: Coomassie blue-stained gel. **(b)** shows the migration patterns of HSP90 in the absence of GI and ZTL. **(c)** shows co-elution of HSP90, GI -HA and ZTL in the same high molecular weight elution fraction at times of maximum GI-HA expression, consistent with presence of all three in the same complex. **(d)** shows co-elution of HSP90, GI-HA and ZTL in the same high molecular weight elution fraction is enhanced during the photoperiod under constitutive expression of GI-HA, consistent with light-enhanced presence of GI and ZTL in the same complex. Band intensity of GI-HA and ZTL in **(e,f)** GI:GI-HA and **(g,h)** 35S:GI-HA was quantitated using Image Lab software (ver. 4.1, Bio-Rad) and all bands were normalized to the band of each protein showing highest intensity (set to 1).Total protein was extracted from 10-day old seedlings and eluted using a Superdex200 10/300 column. Eluted samples were TCA-precipitated, immunoblotted and probed with anti-HA (for GI-HA), anti-HSP90 and anti-ZTL antibodies. Molecular mass markers (blue dextran, 2000 kD a; thyroglobulin, 660 kDa; ferritin, 440 kDa; β-amylase, 200 kDa; alcohol dehydrogenase, 150 kDa; BSA, 67 kDa) were independently eluted using same equilibrated column. Arrowhead in **(c)** and **(d)** shows ZTL position. Data represent means ± s.e (*n*=3).

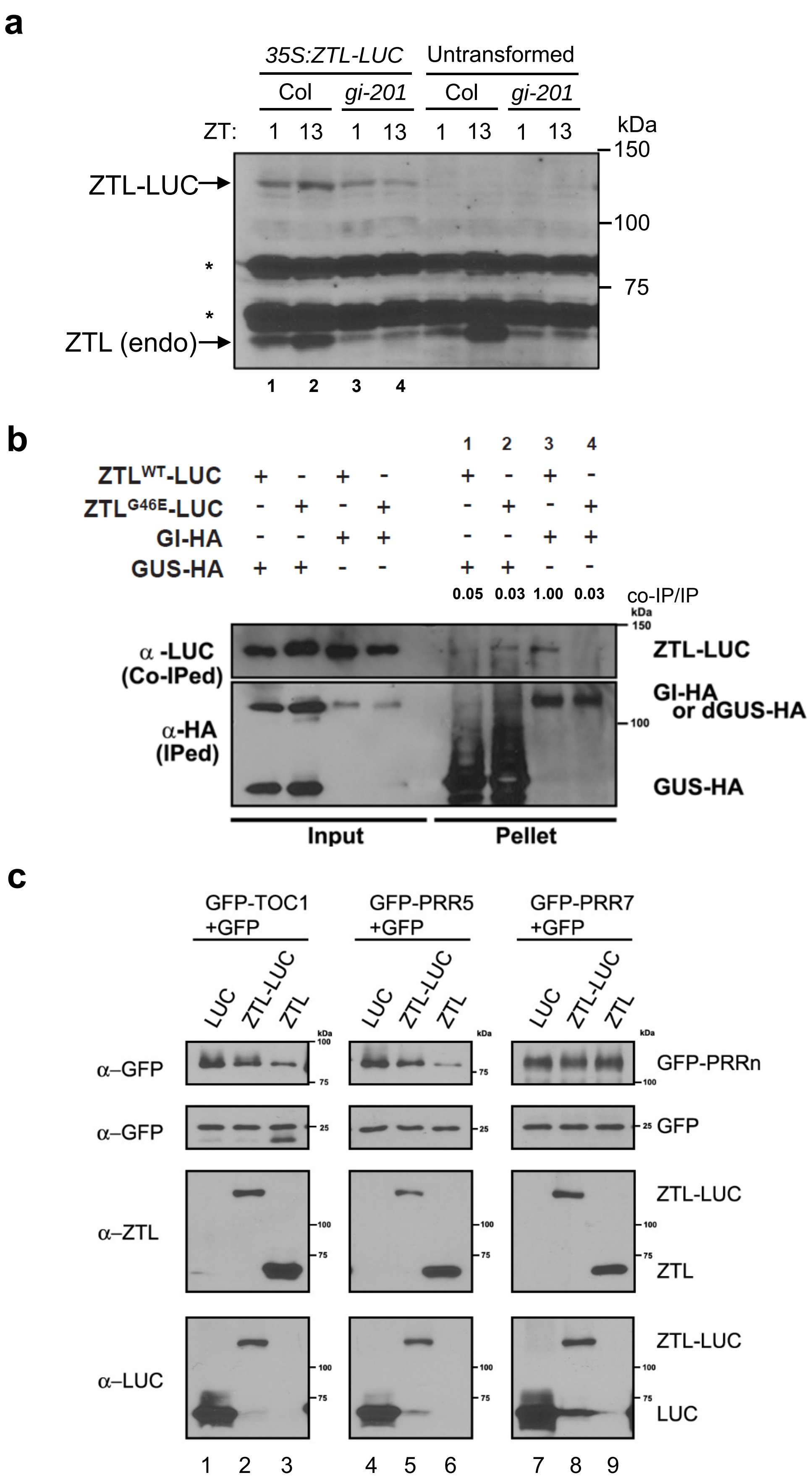
a
35S:ZTL-LUC
Untransformed
Col
gi-201
Col
gi-201
ZT: 1 13 1 13 1 13 1 13
kDa
150
100
75
ZTL-LUC
*
*
ZTL (endo)
1 2 3 4
b
1 2 3 4
ZTL^WT-LUC + - + - + - + -
ZTL^G46E-LUC - + - + - + - +
GI-HA - - + + - - + +
GUS-HA + + - - + + - -
0.05 0.03 1.00 0.03
co-IP/IP
kDa
150
α -LUC (Co-IPed)
ZTL-LUC
α-HA (IPed)
GI-HA or dGUS-HA
100
GUS-HA
Input
Pellet
c
GFP-TOC1 +GFP
GFP-PRR5 +GFP
GFP-PRR7 +GFP
LUC
ZTL-LUC
ZTL
kDa
α–GFP
GFP-PRRn
α–GFP
GFP
α–ZTL
ZTL-LUC
ZTL
α–LUC
ZTL-LUC
LUC
1 2 3 4 5 6 7 8 9

Supplementary Fig. 9. ZTL-LUC properties are similar to those of endogenous ZTL.
(a) Protein abundance of ZTL-LUC reflects endogenous ZTL levels. *35S:ZTL-LUC* in Col and *gi-201*, and untransformed Col and *gi-201* seedlings were grown under 12L/12D and harvested at ZT1 and ZT13. SDS-PAGE separated protein extracts were immunoblotted with anti-ZTL antibody. Arrows indicate ZTL-LUC and endogenous ZTL. ZTL-LUC proteins migrated between 100 and 150 kDa and endogenous ZTL below 75kDa. * indicates non-specific proteins. The *35S:ZTL-LUC* line used in all subsequent experiments was chosen as the one that best approximated the level of endogenous ZTL. **(b)** ZTL-LUC and GI protein interaction is allele specific. *35S:ZTL^{WT}-LUC* and *35S-ZTL^{G46E}-LUC* were co-transformed with *CsVMV:GI-HA* or *CsVMV:GUS-HA* into Arabidopsis mesophyll protoplasts. Protein expression from protoplasts was determined by immunoblotting. Soluble fraction (input) and eluates (pellet) after IP with anti-HA antibody are shown. The co-IP/IP ratios of ZTL-LUC to GI-HA or GUS-HA , normalized to the ratio of the GI-HA/ZTL WT value, are shown above the blots for each of the four combinations. **(c)** Abundance of GFP-TOC1 and GFP-PRR5 proteins are reduced in response to *35S:ZTL-LUC* co-expression. GFP-PRRn (TOC1, PRR5, or PRR7) and GFP were co-expressed with *35S:LUC*, *35S:ZTL-LUC*, or *35S:ZTL* in *N. benthamiana* and protein levels were determined by immuno-blotting with anti-GFP, anti-ZTL and anti-LUC antibody. In **(c)** GFP was used as a reference protein for expression. In **(b)** the dGUS-HA band is a presumed dimer of GUS-HA that migrates at a similar position as GI-HA. Data are representative of two trials.

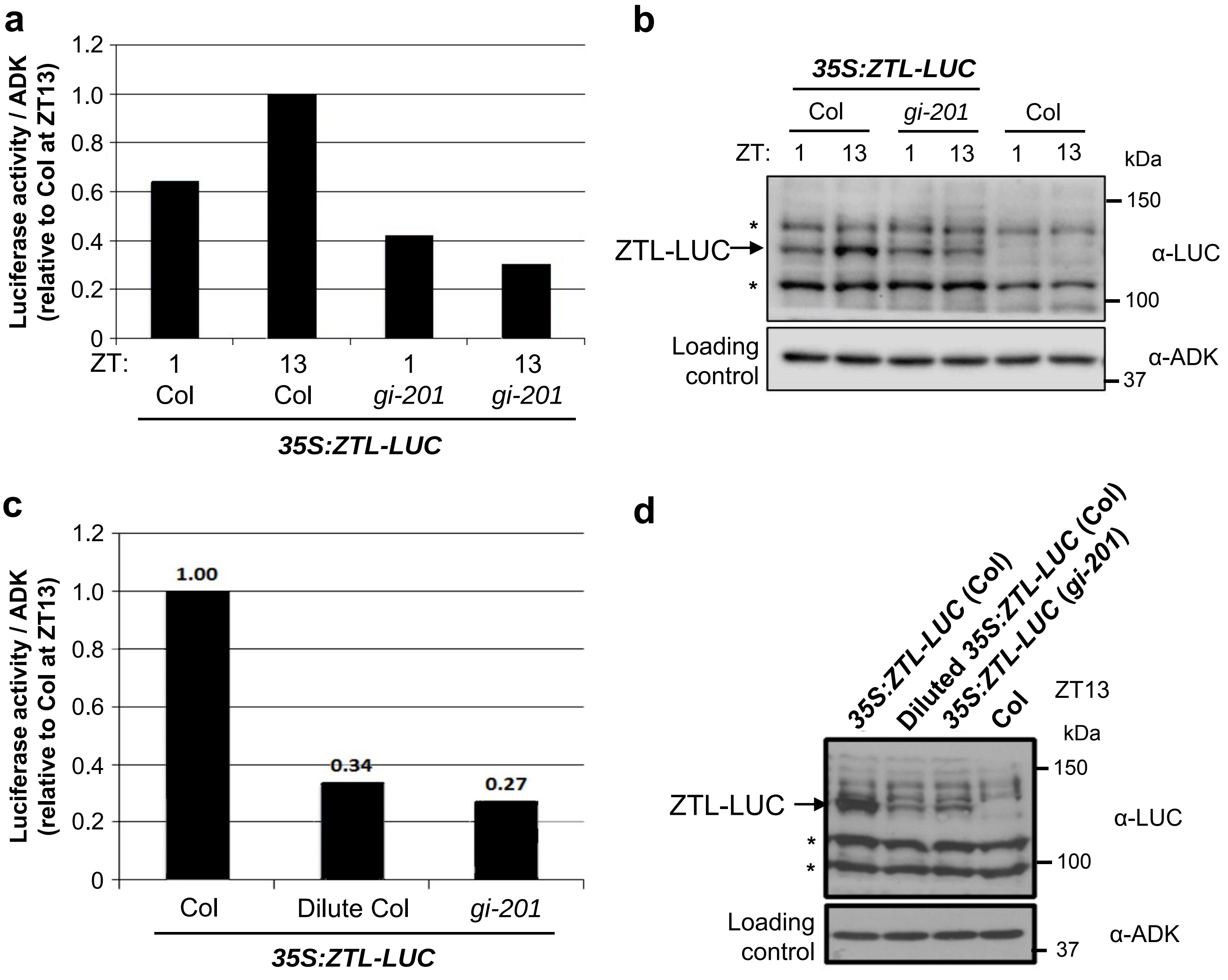

Supplementary Fig. 10. Luciferase activity and protein levels of ZTL-LUC in Col and *gi-201*. (a) Col and *gi-201* plants harboring *35S:ZTL-LUC* were grown in 12L/12D and harvested at ZT1 and ZT13. Total protein was extracted and assayed for luciferase activity. **(b)** The same extracts were separated by SDS-PAGE and immunoblotted with anti-LUC and anti-adenosine kinase (ADK) antibody. Luciferase activity in **(a)** is adjusted relative to ADK protein level and normalized to luciferase activity in Col (*35S:ZTL-LUC*) harvested at ZT13. Arrow indicates ZTL-LUC. * indicates non-specific cross-reactivity. WT Col used to identify ZTL-LUC band. ADK protein used as loading control. Data are representative of four trials. Luciferase activity **(c)** and protein amount **(d)** of ZTL-LUC in Col and *gi-201* at ZT13. Luciferase activity and protein amount were measured as in **(a)** and **(b)** but with dilution of total protein extracts from *35S:ZTL-LUC* in Col at ZT 13 with protein extracts from untransformed Col by a ratio of 1 to 3. This was done to bring ZTL-LUC protein in the WT background to levels similar to ZTL-LUC in *gi-201* to allow for more accurate quantitation at this time point. This approach was used for the final data presented in Fig. 3. Data are representative of eight trials.

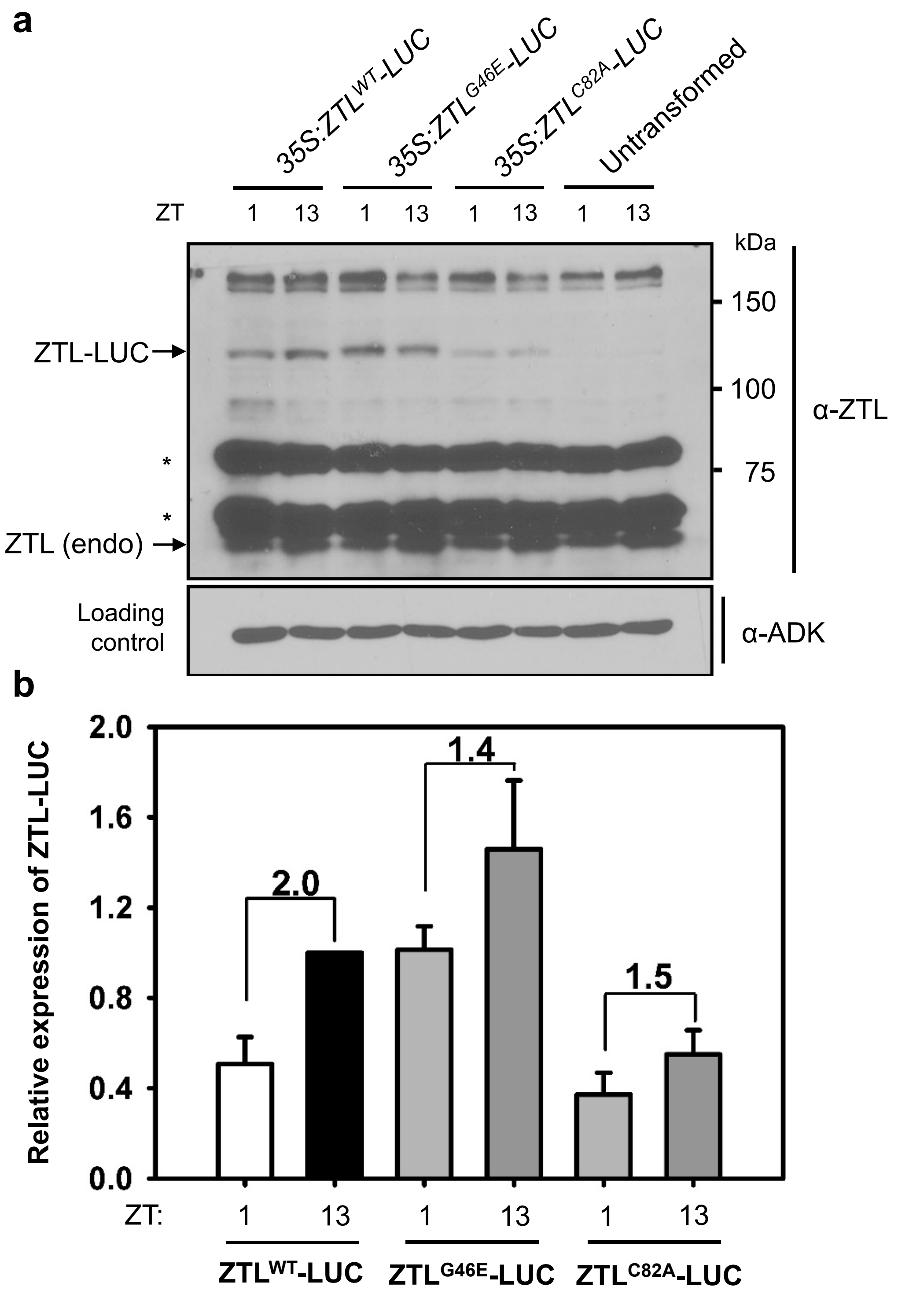

Supplementary Fig. 11. Expression levels of ZTL^{WT}-LUC, ZTL^{G46E}-LUC and ZTL^{C82A}-LUC. **(a)** *35S:ZTL(^{WT}, ^{G46E}, or ^{C82A})-LUC* and untransformed plants seedlings were grown under 12L/12D, harvested at ZT1 and ZT13 and immunoblotted protein extracts were probed with anti-ZTL antibody. Arrows indicate ZTL-LUC and endogenous ZTL. * indicates non-specific proteins. Data are representative of three trials. ZTL^{G46E}-LUC migrates more slowly than ZTL^{WT}-LUC . **(b)** Quantitation of relative expression ZTL-LUC proteins from **(a)**. ZTL-LUC levels in each sample were normalized to ADK and further normalized to ZTL^{WT}-LUC at ZT13 within each trial. Data are means ± s.e. of three independent trials. Numbers indicate the ratio of ZTL-LUC proteins expressed at ZT 13 to at ZT1 for each ZTL variant.

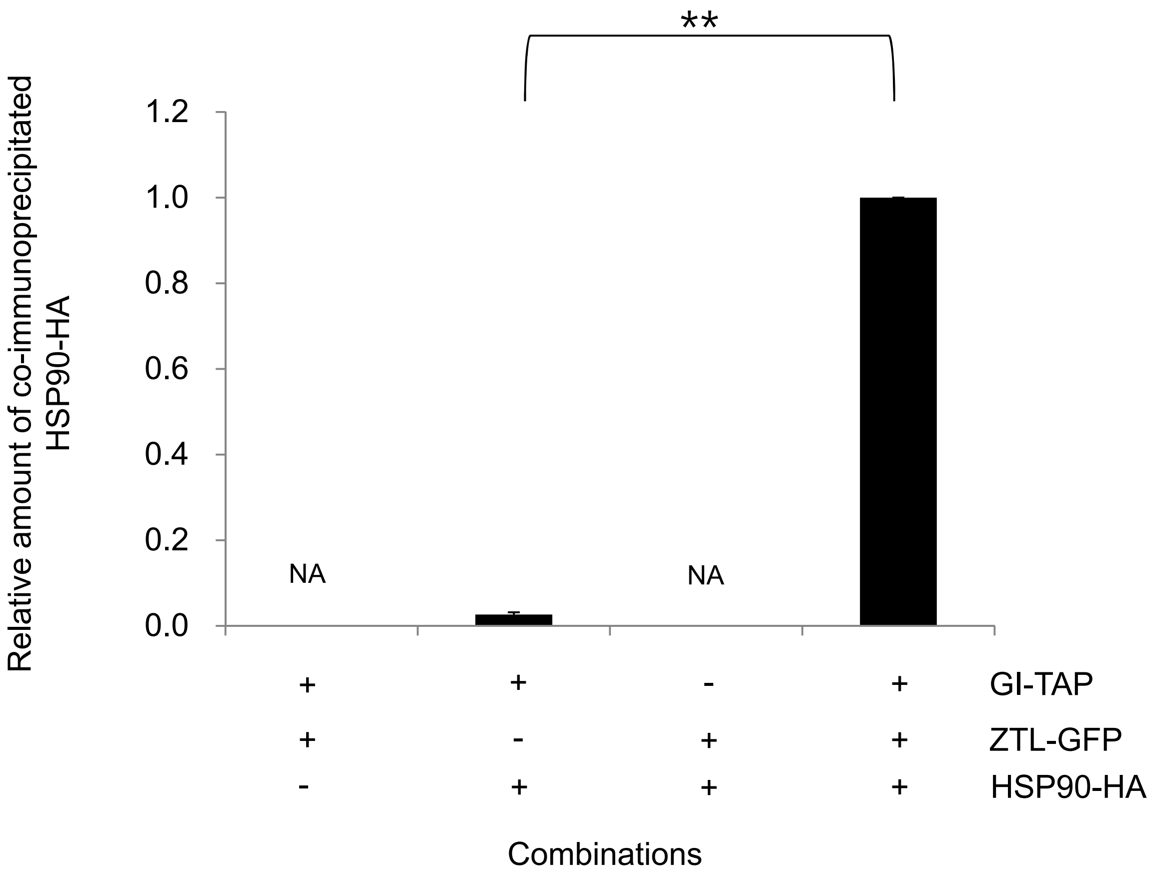

Supplementary Fig. 12. Graphical summary of the serial detections of co-immunoprecipitated HSP90-HA from the 2nd supernatants (2nd Sup.) of Figure 4c. ** $P<0.001$ calculated from one-way ANOVA. Quantitations based on long exposures of 3 independent experiments. NA = "not acquired" since the initial HSP90-HA in the 2nd Sup. is not detectable in these instances.

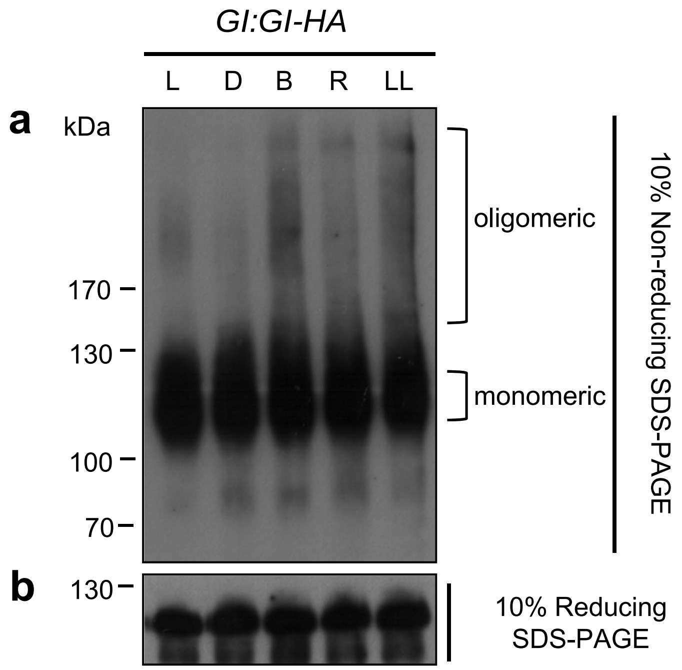

Supplementary Fig. 13. Light-dependent oligomeric complex of GI. Ten-day-old *GI:GI-HA* plants grown in 12L/12D were harvested at ZT6 (L) and ZT18 (D). The plants were also harvested at ZT18 under blue (B), red (R) and constant light (LL). Total protein was extracted and separated by **(a)** 10 % non-reducing SDS-PAGE and **(b)** 10 % reducing SDS-PAGE. Immunoblot analysis was performed using anti-HA antibody.

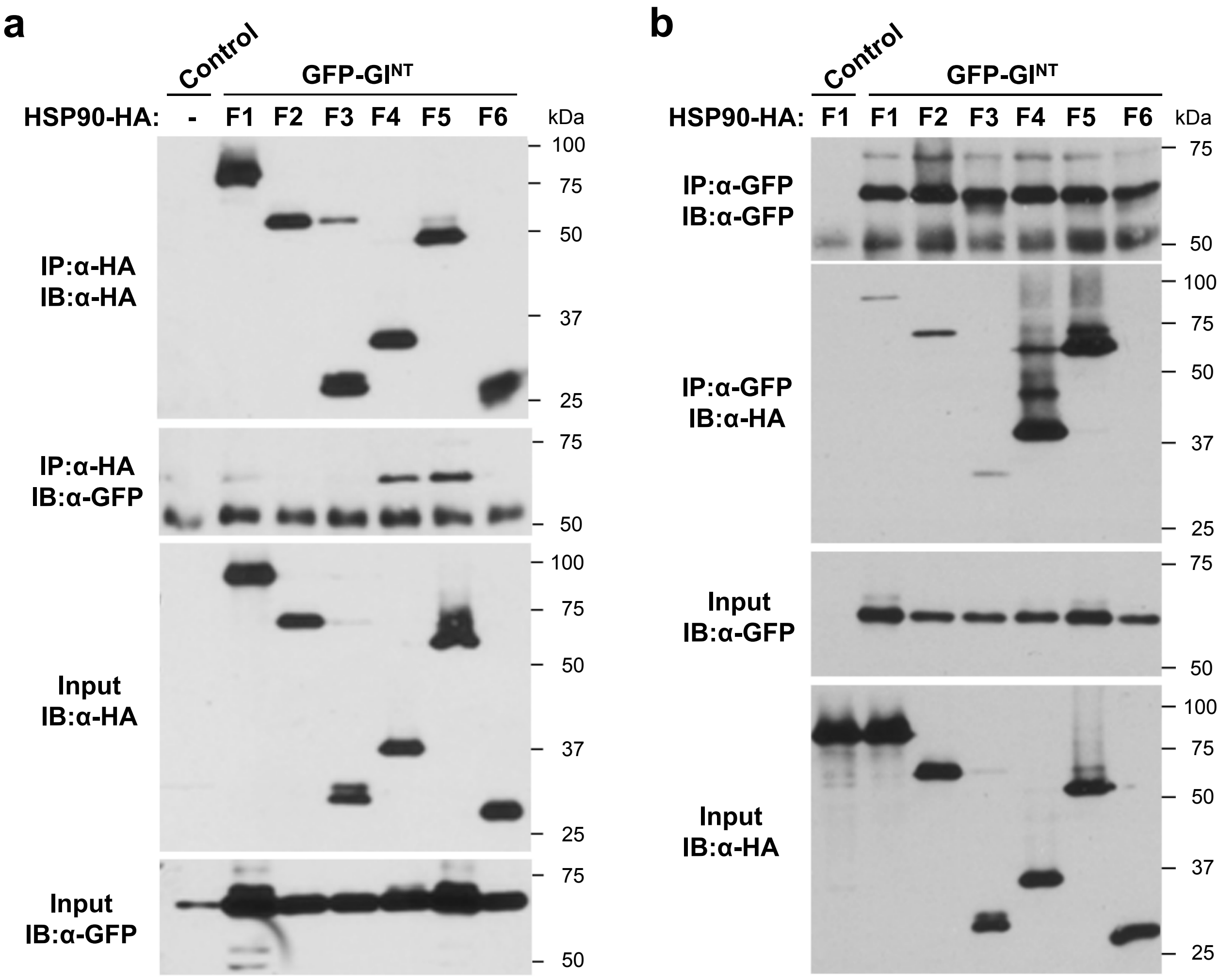

Supplementary Fig. 14. N-terminus of GI interacts with HSP90 and its deletion *in vivo*. HSP90 deletion interactions with GI^{NT} (aa 1-391). The co-IP assays were performed either with HA antibody **(a)** or GFP antibody **(b)** as the primary antibody. In both cases, the strongest interactions between GI^{NT} and HSP90 are found in HSP90 F4 (HSP90 264-524aa; MD) and HSP90 F5 (HSP90 264-706aa; MD+DD), indicating the middle domain of HSP90 is sufficient to interact with GI^{NT}, but the presence of the NBD weakens this interaction (as in F1-F3). Agrobacteria harboring GFP-GI^{NT} and HSP90 or respective deletions (3xHA-tagged) were co-infiltrated into *N. benthamiana* leaves. Anti-GFP immunoprecipitations (IP) were followed by detection of co-immunoprecipitated HSP90-HA (co-IP). Representative of three trials with similar results. HSP90 domain structure and respective deletion scheme as in Fig. 4. IP: immunoprecipitation antibody; IB: immunoblot antibody.

Supplementary Fig. 15

Note: The following pages (Supplementary Figure 15) show original images of immunoblots and gels used in various main and supplementary figures when those images were cropped. They are shown in the context of the annotation of the original figure.

Fig. 4a

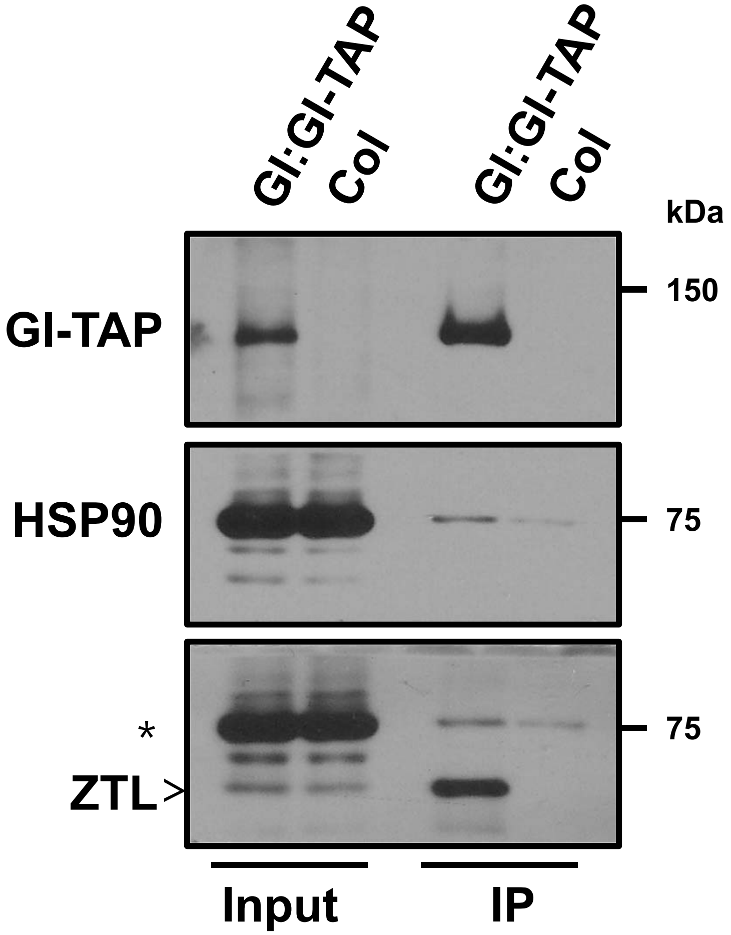

Fig. 4b

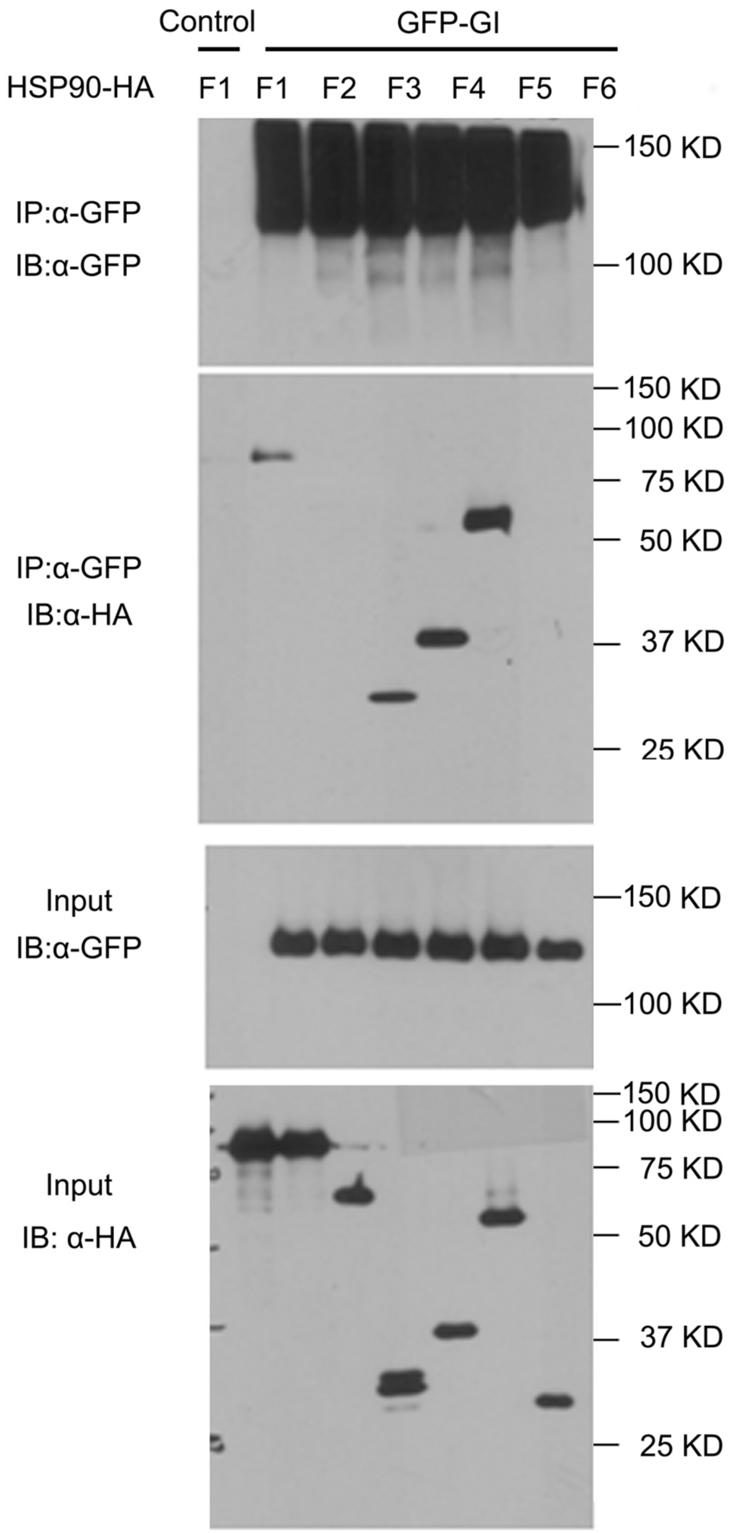

Control
GFP-GI
HSP90-HA F1 F1 F2 F3 F4 F5 F6
IP:α-GFP
IB:α-GFP
150 KD
100 KD
IP:α-GFP
IB:α-HA
150 KD
100 KD
75 KD
50 KD
37 KD
25 KD
Input
IB:α-GFP
150 KD
100 KD
Input
IB: α-HA
150 KD
100 KD
75 KD
50 KD
37 KD
25 KD

Fig. 4c

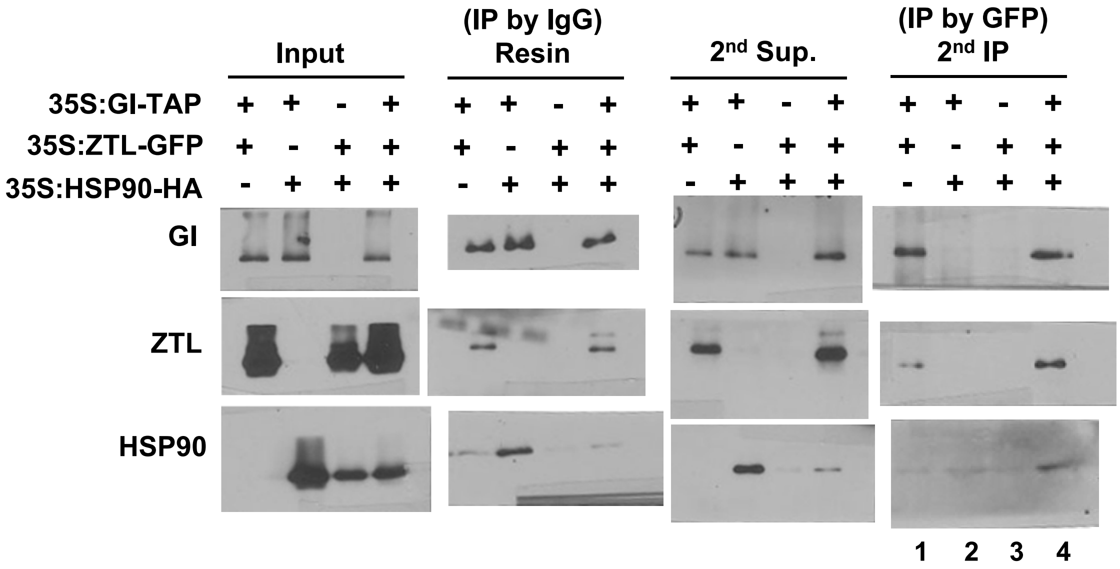
Input
(IP by IgG) Resin
2nd Sup.
(IP by GFP) 2nd IP
35S:GI-TAP + + - +
35S:ZTL-GFP + - + +
35S:HSP90-HA - + + +
GI
ZTL
HSP90
1 2 3 4

Supplementary. Fig. 2a

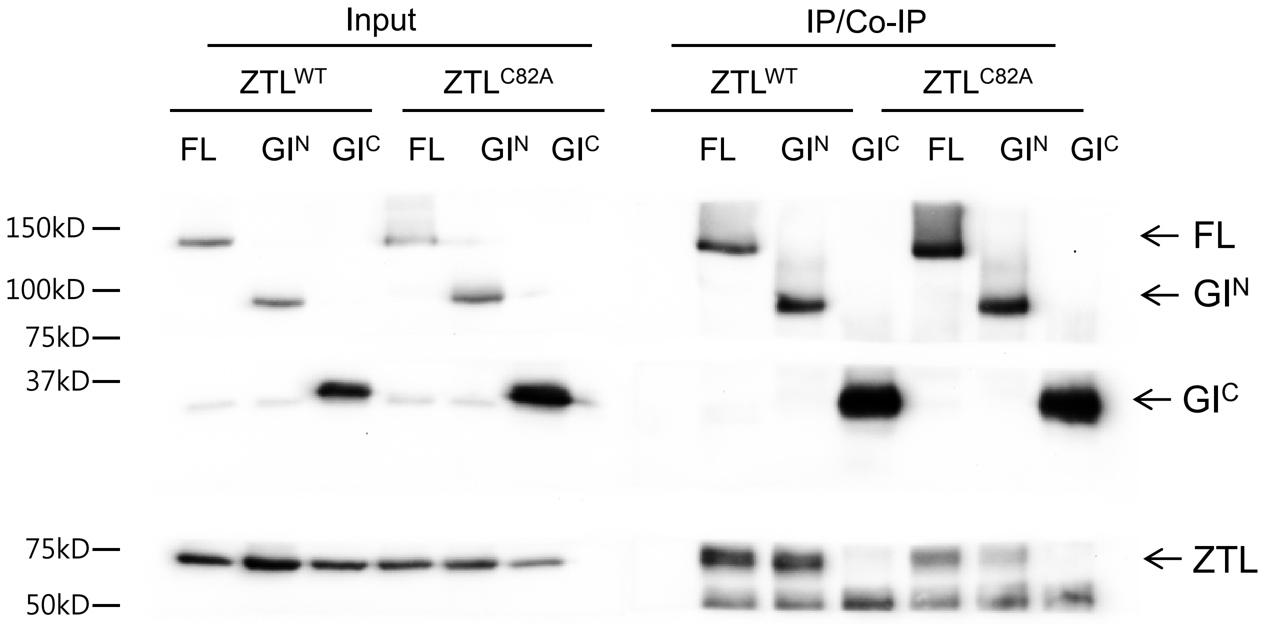

Supplementary. Fig. 3a

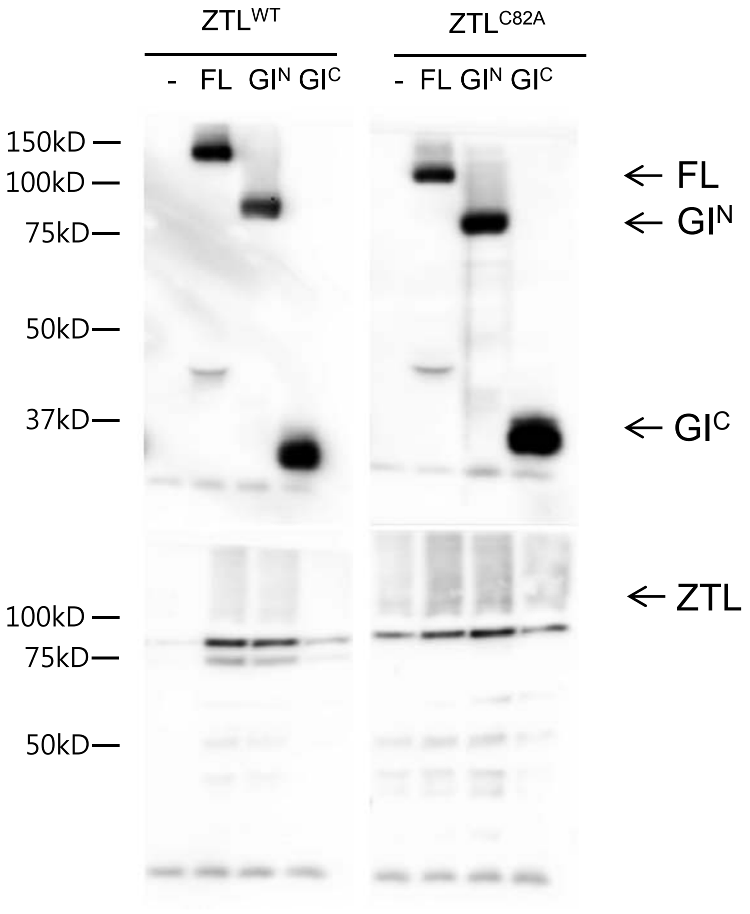

Supplementary. Fig. 8

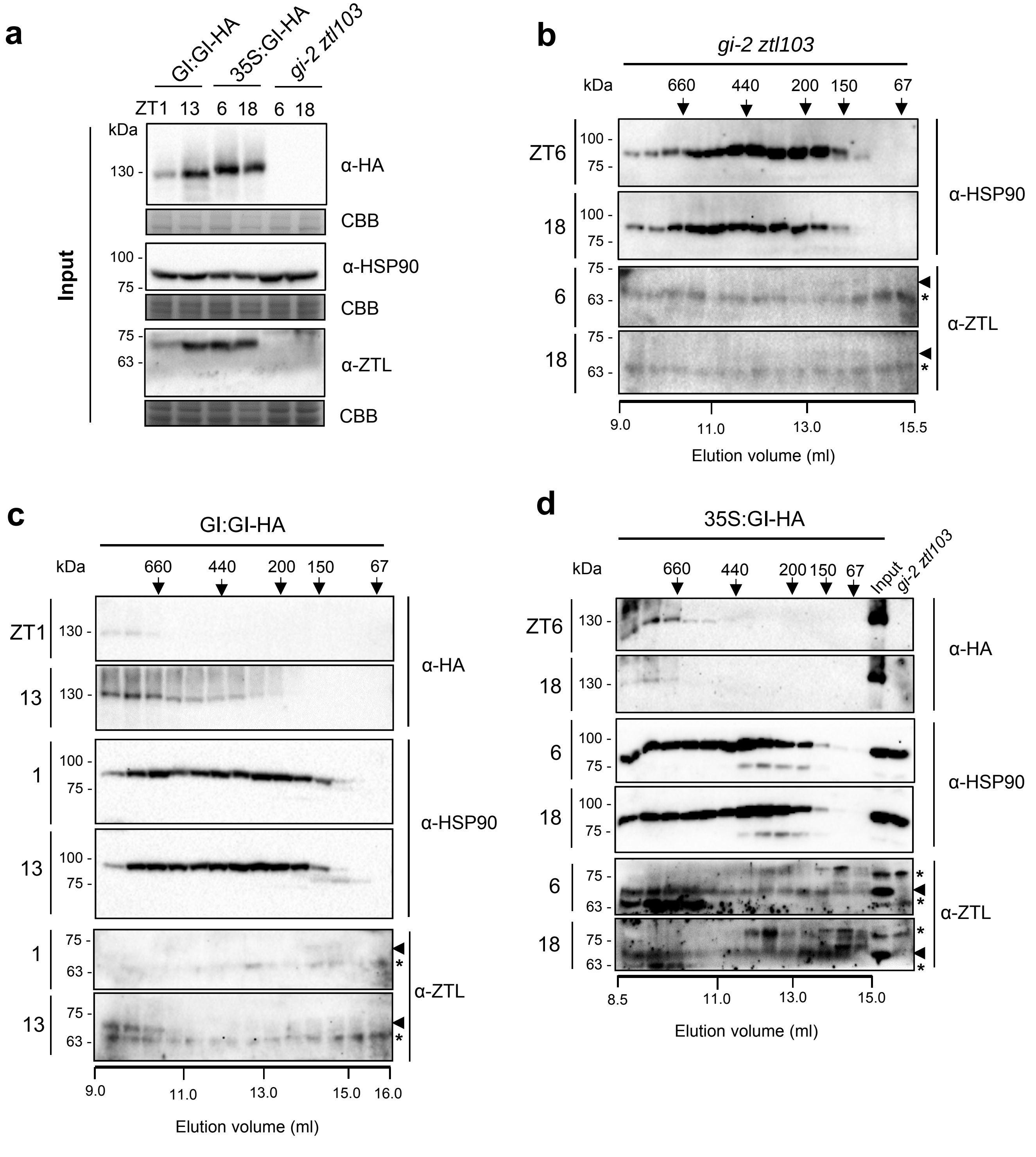

Suppl. Fig. 9a

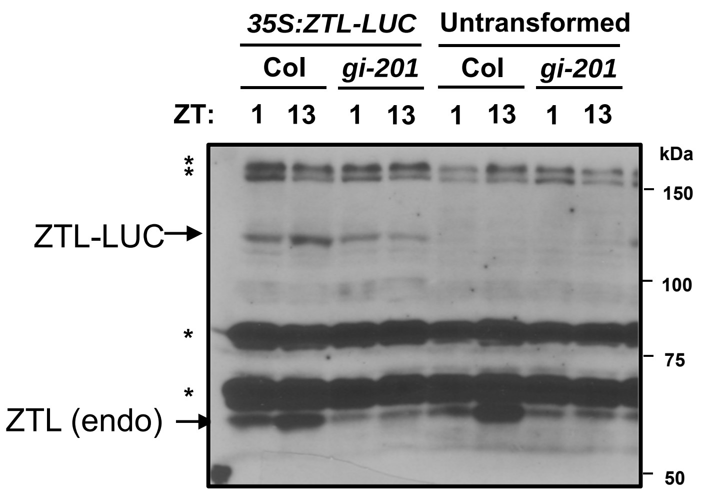

Suppl. Fig. 9b

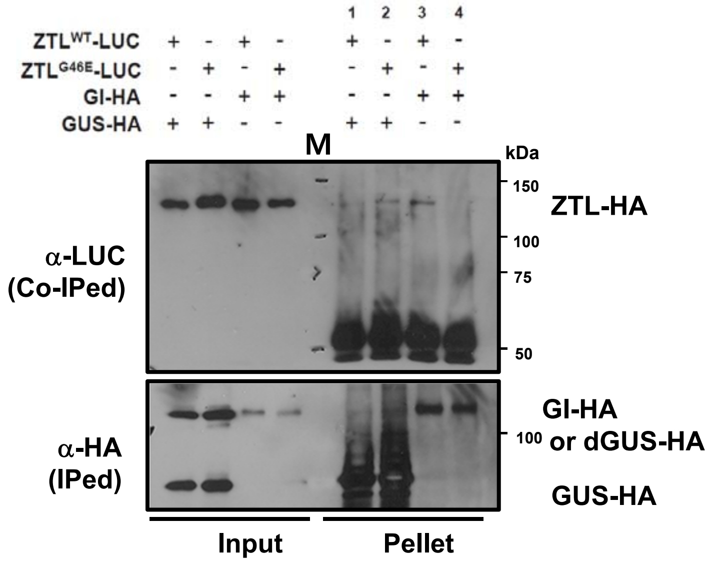

Supplementary. Fig. 9c

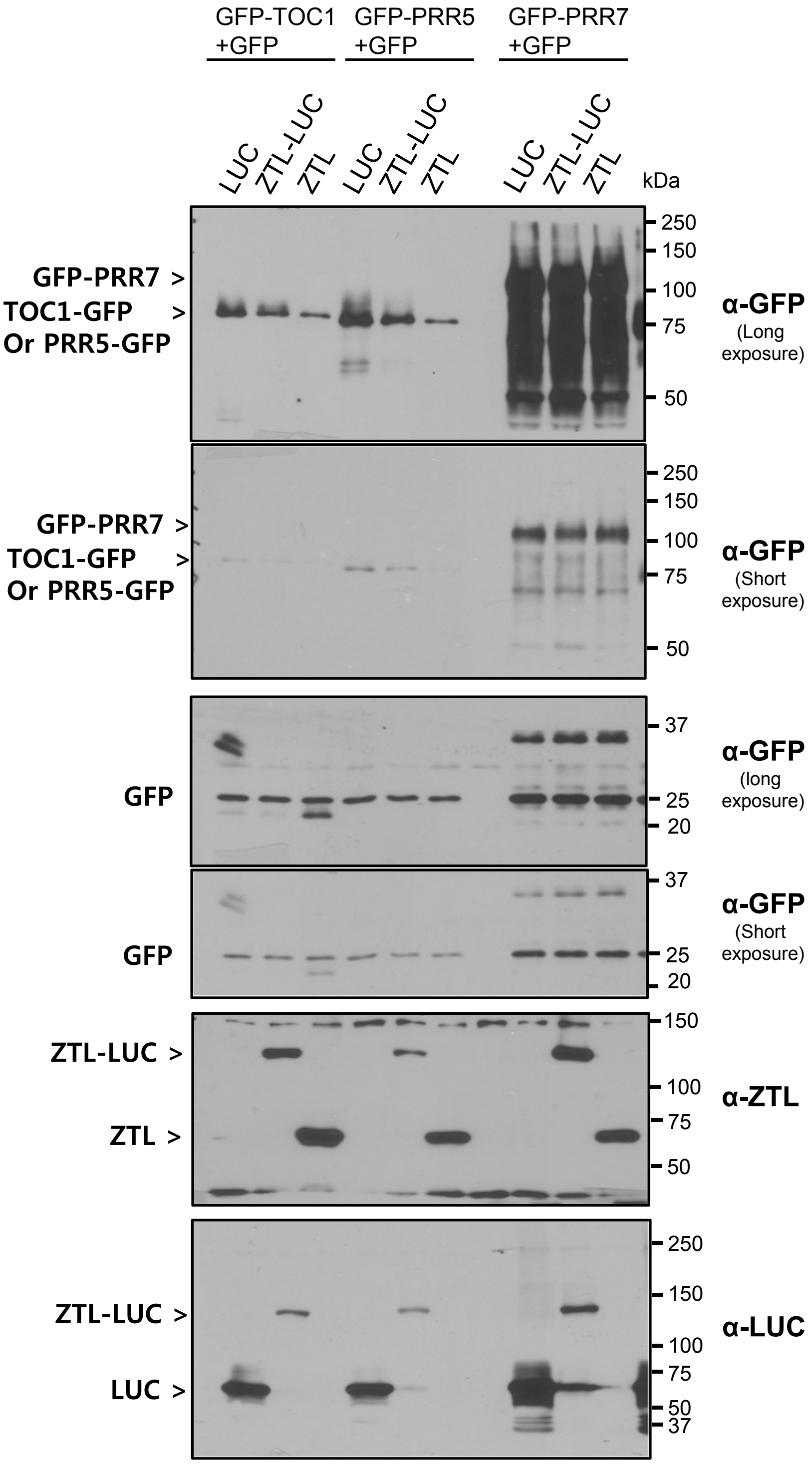

Supplementary. Fig 10b

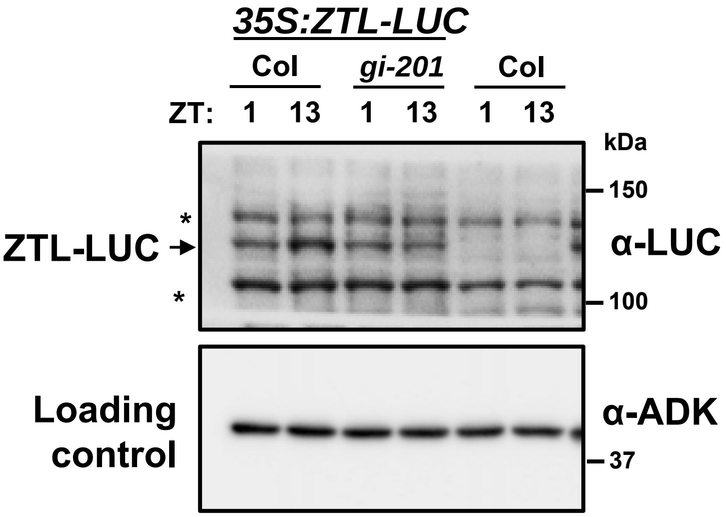

Supplementary. Fig 10d

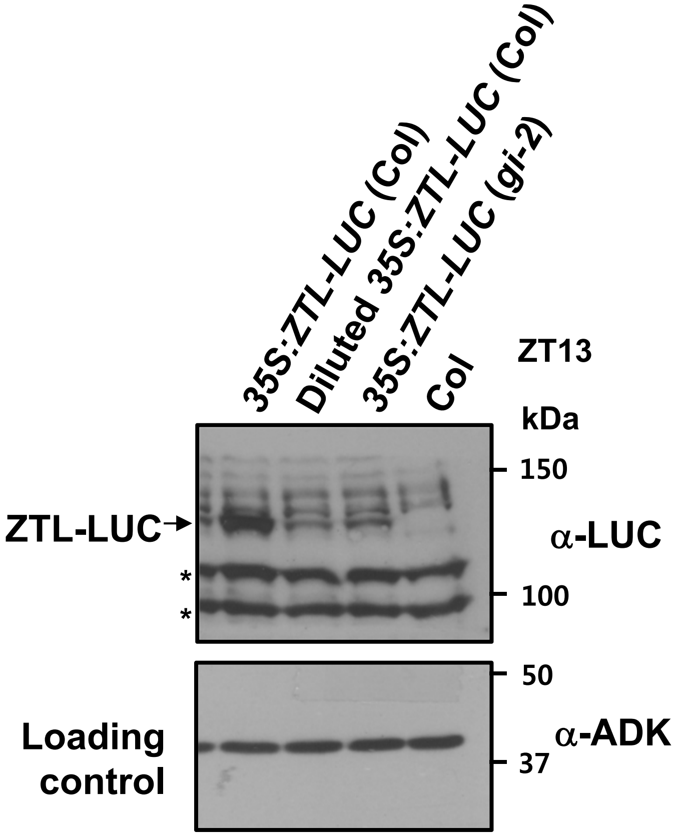

Supplementary. Fig 11

a

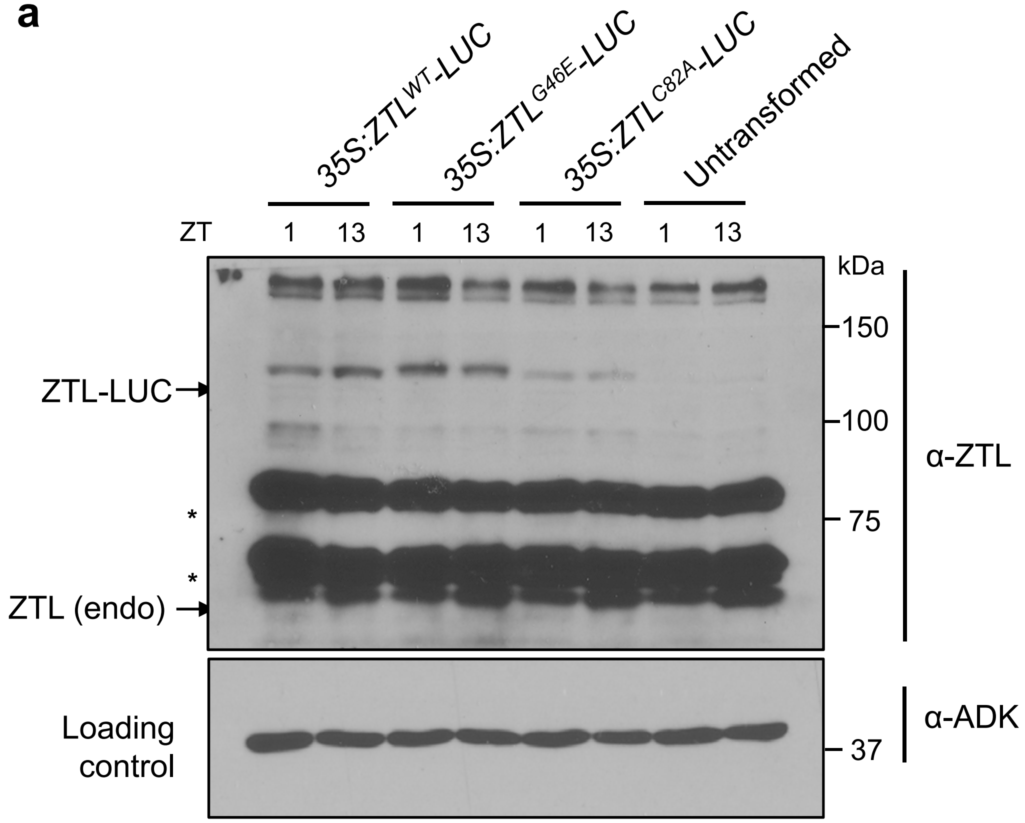

Supplementary. Fig. 14

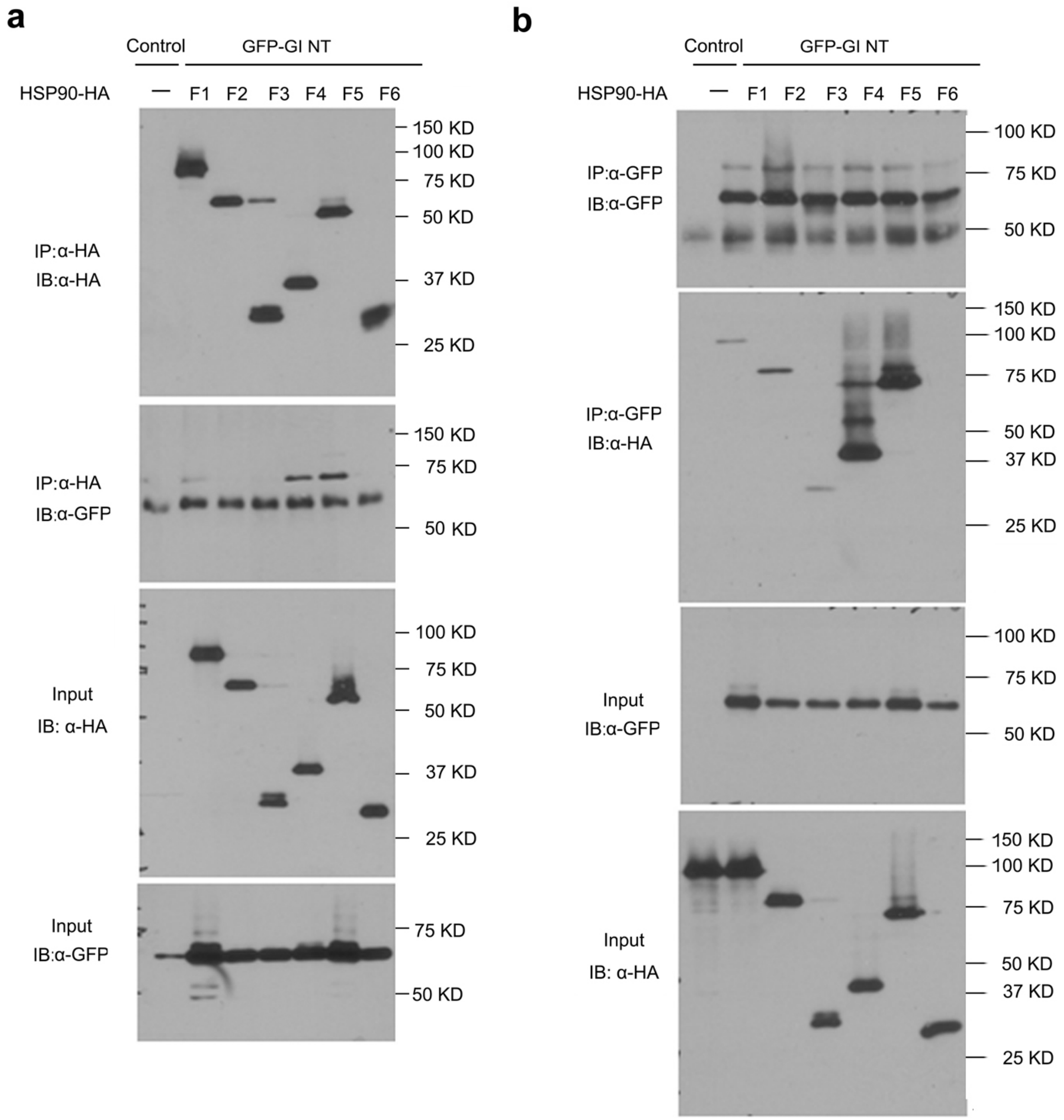

Supplementary Fig. 15. Uncropped images of immunoblots and gels.

Gels and blots used in various main and supplementary figures when those images were cropped are shown. They are presented in the context of the annotation of the original figure**.**

Supplementary Table1. Identification of the expressed proteins by MALDI-TOF/TOF-MS.

No[a)]	Accession	Description (Mr/pI[b)])	Covered protein %	Matched peptide #	Matched peptide sequence
1	At1G22770	Protein GIGANTEA (*Arabidopsis thaliana*) (129163/6.6)	10% (82/858AA's)	6	RWIDGLQFSSLLWPPPRD<br>RYYAIATPSATQRL<br>RNWMHLHFLRA<br>RAGVAADAAAALLFRI<br>KVLEYLPRG<br>KIFVATVETILSRT
2	At1G22770	Protein GIGANTEA (*Arabidopsis thaliana*) (129163/6.6)	25% (62/253AA's)	3	KAAAAVVLQAERE<br>RNHDRPESLVILASASDLLLRA<br>RATDGMLVDGEACTLPQLELLEATARA
3	P0AEY0 (P02928)	Maltose-binding periplasmic protein (*Escherichia coli* O157:H7) (43360/5.53)	30% (118/396AA's)	7	KFPQVAATGDGPDIIFWAHDRF<br>RFGGYAQSGLLAEITPDKA<br>KLYPFTWDAVRY<br>KLIAYPIAVEALSLIYNKD<br>KAGLTFLVDLIKN<br>KEFLENYLLTDEGLEAVNKD<br>KSYEEELAKDPRI

a) Numbers correspond to the bands marked on Supplementary Fig 4.
b) Theoretical MW (Da) and pI values.

Supplementary Table 2. Primer sequences used in this study.

Name	Primer sequence (5’ to 3’)	Purpose
GI^{N}	Forward: 5’-GGGGACAAGTTTGTACAAAAAAGCAGGCTCCATGGCTAGTTCATCTTCA-3’<br>Reverse: 5’-GGGGACCACTTTGTACAAGAAAGCTGGGTCTTATTCATCTGAATGTCTAGCATA-3’	Gateway cloning
GI^{C}	Forward: 5’-GGGGACAAGTTTGTACAAAAAAGCAGGCTCCATGATTGCTGCTCCTGAAATC-3’<br>Reverse: 5’-GGGGACCACTTTGTACAAGAAAGCTGGGTCTTATTGGGACAAGGATATAG-3’	Gateway cloning
GI^{NT}	Forward: 5’-ACTGGATCCGATGGCTAGTTCATCTTCATC-3’<br>Reverse: 5’-CTAGATATCTTAAGCGGCTGCAGCATCAGCAG-3’	Gateway cloning
ZTL^{C82A}	Forward: 5'-GTTCTCGGAGGAAATGCCCGCTTCTTGCAATGT-3'<br>Reverse: 5'-ACATTGCAAGAAGCGGGCATTTCCTCCGAGAAC-3'	Mutagenesis
HSP90.1 F1	Forward:5'-TTTGGATCCGGATGGCGGATGTTCAGATGGCTG-3'<br>Reverse: 5'-TTTGCGGCCGCTTAGTCGACTTCCTCCATCTTGCT-3'	Gateway cloning
HSP90.1 F2	Forward:5'-TTTGGATCCGGATGGCGGATGTTCAGATGGCTG-3'<br>Reverse:5'-TTTGCGGCCGCTTATTCTTTAGTCGCAGAAACAAG-3'	Gateway cloning
HSP90.1 F3	Forward:5'-TTTGGATCCGGATGGCGGATGTTCAGATGGCTG-3'<br>Reverse:5'-TTTGCGGCCGCTTAAGGGTAACTGATGAACTCAGA-3'	Gateway cloning
HSP90.1 F4	Forward:5'-TTTGGATCCGGTGGGAACTCATCAACAAGCAG-3'<br>Reverse:5'-TTTGCGGCCGCTTATTCTTTAGTCGCAGAAACAAG-3'	Gateway cloning
HSP90.1 F5	Forward:5'-TTTGGATCCGGTGGGAACTCATCAACAAGCAG-3'<br>Reverse: 5'-TTTGCGGCCGCTTAGTCGACTTCCTCCATCTTGCT-3'	Gateway cloning
HSP90.1 F6	Forward:5'-TTTGGATCCGGTTCGAGAATCTGTGCAAGACG-3'<br>Reverse: 5'-TTTGCGGCCGCTTAGTCGACTTCCTCCATCTTGCT-3'	Gateway cloning

References

1. Saitou, N., Nei, M. The neighbor-joining method: a new method for reconstructing phylogenetic trees. *Molecular Biology and Evolution* **4,** 406 (1987).

2. Tamura, K.*et al.* MEGA6: Molecular Evolutionary Genetics Analysis version 6.0. *Molecular Biology and Evolution* **30,** 2725 (2013).
